# Pleiotropic constraints promote the evolution of cooperation in cellular groups

**DOI:** 10.1371/journal.pbio.3001626

**Published:** 2022-06-03

**Authors:** Michael A. Bentley, Christian A. Yates, Jotun Hein, Gail M. Preston, Kevin R. Foster

**Affiliations:** 1 Department of Zoology, University of Oxford, Oxford, United Kingdom; 2 Department of Biochemistry, University of Oxford, Oxford, United Kingdom; 3 Department of Mathematical Sciences, University of Bath, Bath, United Kingdom; 4 Department of Statistics, University of Oxford, Oxford, United Kingdom; 5 Department of Plant Sciences, University of Oxford, Oxford, United Kingdom; University of Bern, SWITZERLAND

## Abstract

The evolution of cooperation in cellular groups is threatened by lineages of cheaters that proliferate at the expense of the group. These cell lineages occur within microbial communities, and multicellular organisms in the form of tumours and cancer. In contrast to an earlier study, here we show how the evolution of pleiotropic genetic architectures—which link the expression of cooperative and private traits—can protect against cheater lineages and allow cooperation to evolve. We develop an age-structured model of cellular groups and show that cooperation breaks down more slowly within groups that tie expression to a private trait than in groups that do not. We then show that this results in group selection for pleiotropy, which strongly promotes cooperation by limiting the emergence of cheater lineages. These results predict that pleiotropy will rapidly evolve, so long as groups persist long enough for cheater lineages to threaten cooperation. Our results hold when pleiotropic links can be undermined by mutations, when pleiotropy is itself costly, and in mixed-genotype groups such as those that occur in microbes. Finally, we consider features of multicellular organisms—a germ line and delayed reproductive maturity—and show that pleiotropy is again predicted to be important for maintaining cooperation. The study of cancer in multicellular organisms provides the best evidence for pleiotropic constraints, where abberant cell proliferation is linked to apoptosis, senescence, and terminal differentiation. Alongside development from a single cell, we propose that the evolution of pleiotropic constraints has been critical for cooperation in many cellular groups.

## Abstract 2

The evolution of cooperation in cellular groups is threatened by lineages of cheaters that proliferate at the expense of the group. In this study, an age-structured model of cellular groups shows that pleiotropy promotes the evolution of cooperation and may have been important for the origins of multicellularity.

## Introduction

There is widespread cooperation in cellular groups where cells invest in costly traits that benefit all cells in the vicinity, such as bacteria that secrete an extracellular enzyme to digests nutrients or the more complex coordinated phenotypes of multicellular organisms. Cooperative traits can require that cells forego their own reproductive interests in favour of the reproductive interests of the group as a whole [1,2]. This effect, in turn, can lead to the evolution of noncooperative lineages—sometimes known as “cheaters”—that make use of collective benefits without investing in them and threaten cooperative function [3].

**Fig 1:**
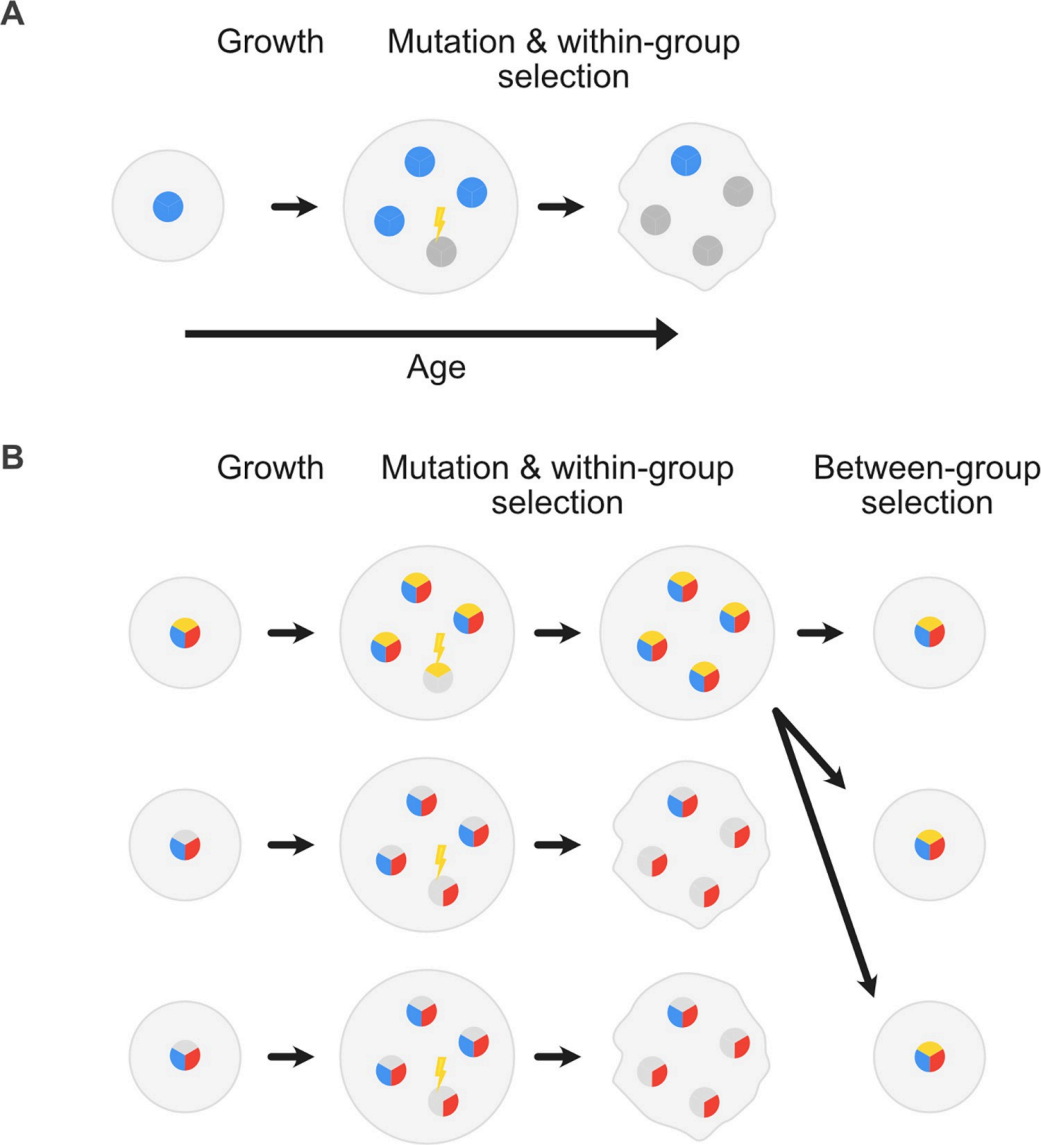
The problem of cooperation and the evolution of pleiotropy. (A) Mutation–selection dynamics can undermine clonal multicellular groups. Mutation of cooperative cells (blue) can generate noncooperative cells (dark grey) that do not pay the costs of cooperation but reap its rewards. These cheater lineages can spread within multicellular groups because they divide more rapidly than wild-type cells. Invasion of spontaneous mutant lineages can lead to a breakdown in group function (distorted shape). (B) Pleiotropy promotes cooperation in our model because it leads to between-group selection on the rate at which cooperation breaks down within groups as they age. Beginning with groups founded by 1 pleiotropic and 2 nonpleiotropic lineages, we see that groups founded by the nonpleiotropy lineages loose function and are eventually replaced by the pleiotropic lineage. Cells are coloured in 3 parts according to whether they display cooperation (blue), a privately beneficial trait (red), and pleiotropy (yellow), whereby the cooperative and private traits are linked. Mutations can make some traits inactive (grey). When cooperation is lost in a pleiotropic cell lineage (top row), the cells also lose their private trait, which stops them from proliferating. Group function is thereby preserved. When cooperation is lost in a nonpleiotropic lineage (second row), cheater lineages emerge that spread and group function is lost. As a result, pleiotropic groups thrive relative to nonpleiotropic ones and seed more groups, giving rise to between-group selection for pleiotropy.

The potential for cheater lineages is well documented in microbes. Mutants lacking a range of cooperative traits have been shown to outcompete wild-type cells [4–10] and occur in the field and clinic [11–13]. Such observations beg the question of how cooperation persists over evolutionary time. A key explanation is that many cellular groups, both in microbes and multicellular organsims, are recently derived from a single cell (clonal) [1,2,14,15]. In the terminology of sociobiology, this leads to high relatedness and kin selection, which is a major driver of cooperation across many systems [16,17]. The argument is that, when cell groups are clonal, interactions between cooperative and cheater genotypes are prevented, which allows cooperative genotypes to prosper as cheater genotypes lose the shared benefits of cooperation. While kin selection is undoubtedly important [1,2,14,15], this explanation neglects a key feature of the biology of cellular groups: Even in a group founded from a single cell, cooperation can still break down due to the emergence of mutant noncooperators from within [18–20] (Fig 1). Indeed, with nonzero mutation rates, the question is when, not if, these lineages will emerge.

Given the potential for cheater lineages, it has been suggested that genetic architecture can evolve to help stabilise cooperation. When the expression of a cooperative trait is linked to a private trait that helps a cell to survive or divide, mutations that ablate cooperation can also ablate the private trait and, thereby, stop the evolution of cheater lineages. This pleiotropic linkage of cooperative traits and private (personally benefitial) traits has been identified experimentally to be a mechanism that helps to promote cooperation in multiple microbial species [21–25]. In addition, an agent-based model of microbes found that links between metabolic and secretion genes helped to stabilise cooperation [26], and a theoretical study suggested that pleiotropy can promote niche construction, which is related to cooperation [27]. These studies raise the possibility that the evolution of pleiotropy might be a general mechanism to promote cooperation in cellular groups [28].

However, a recent theoretical paper argued broadly against the idea that pleiotropy is an explanation for the evolution of cooperation [29]. In particular, the authors argued that pleiotropy only evolves under conditions when kin selection is already operating to stabilise cooperation (Fig 2 in [29]), with, at best, a very minor impact on the evolved level of cooperation (i.e., seen in S14 and S16 Figs but not Fig 2 in [29]). As such, they concluded “*Pleiotropy does not help stabilise cooperation over evolutionary time—cooperation is only favoured in the region where Hamilton’s rule is satisfied because of indirect fitness benefits*.*”*

As we disuss in detail in the Supporting information (S1 Text), a limitation of this model is that it did not explicitly capture group-level birth and death events or allow groups to develop for long enough to see the importance of pleiotropy for stabilising the evolution of cooperation. The time allowed for groups to develop is important because the problems with cheater lineages only becomes apparent as groups age (Fig 1). Pleiotropy only becomes subject to significant between-group selection, therefore, in longer-lived groups when cheater mutants have time to threaten the group. We show in the Supporting information that increasing the length of time that groups live for increases the levels of cooperation that evolve via pleiotropy in the model of [29]. However, problematic assumptions such as unbounded explonential growth prevented us from exploring this effect further (S1 Text). We, therefore, decided to develop a novel age-structured multilevel selection model for the evolution of cooperation in cellular groups, including both microbes and multicellular organisms. Our model predicts that pleiotropy is a powerful way to promote the evolution of cellular cooperation.

## Results

We are interested in understanding how multicellular groups founded by cells with pleiotropic constraints function as compared to groups founded by otherwise similar cells that lack these constraints. We follow the effects of pleiotropic links between cooperative traits (that benefit the whole group) and private traits (that benefit the individual cell that carries them) on multilevel selection dynamics using an age-structured modelling approach (see Methods). A group in our model is intended to capture a group of microbes or a proto-multicellular organism, which lacks the division between germ and soma. Groups start from a single cell and display logistic growth up to a carrying capacity *K*, which defines the size of the group at maturity. A second parameter, *λ*, determines the expected life span of a group. This is important because it impacts on the amount of cell turnover that is expected after a group reaches reproductive maturity. Such cell turnover can be major contributor to the number of cell divisions within a multicellular group. For example, high rates of cell turnover occur in bacteria, which commonly live attached to surfaces in structures known as biofilms where dispersing cells are replaced by dividing cells below them [1]. It is also common In multicellular organisms: Members of the genus *Hydra* (Fig 1) can live for several years, while their epithelial cells are estimated to turnover every few days [30].

The fact that groups start from a single cell in our model ensures high relatedness and strong kin selection, which is consistent with microbes that grow in clonal patches [1] and the biology of multicellular organisms [20]. However, we later reduce this within-group relatedness to study its effects on pleiotropy and cooperation. To study the effects of pleiotropy on cooperation, our modelling has to capture the stochastic effects of mutations. For this reason, the heart of the model is a stochastic simulation that captures populations of cells as they grow, and potentially mutate to other genotypes, within a group. However, as we discuss later, we also need to capture the evolutionary effects of cooperation at the group level, which is done with partial differential equations (PDEs) that allow us to capture a large (infinite) number of competing groups. We hope that this novel approach—stochastic simulations embedded in PDEs—will prove useful to understand a wide range of traits under multilevel selection (Methods).

### Pleiotropy slows the breakdown of cooperation within cellular groups

**Fig 2:**
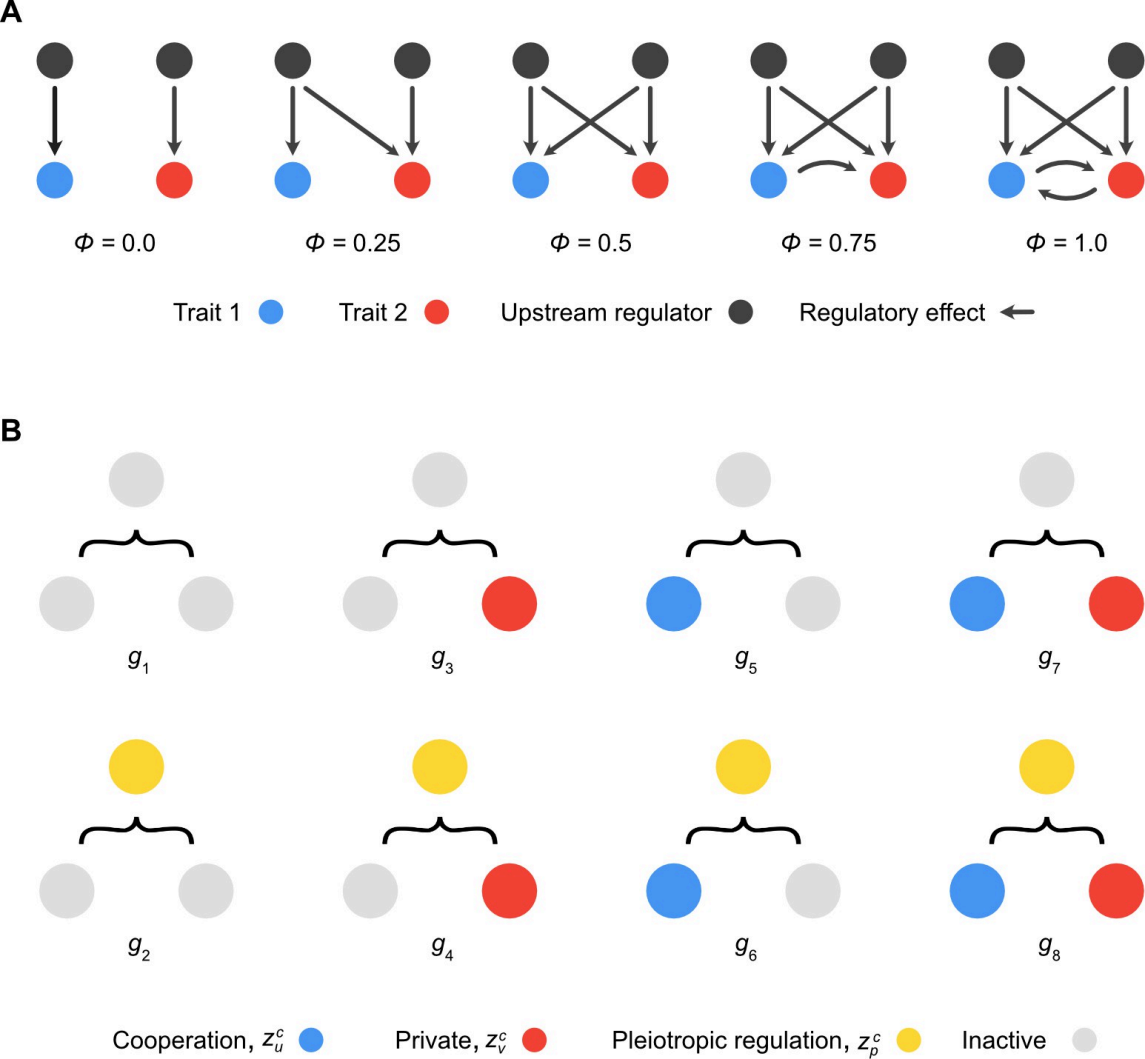
Modelling pleiotropy. (A) One way to measure the strength of pleiotropy in real-world regulatory networks is to compute the ratio of those mutations in the network that simultaneously impact 2 terminal phenotypic traits to the number of genes. We call this measure *ϕ*. In principle, this value can be calculated for any network regulating 2 traits. We show illustrative networks that would generate varying levels of pleiotropy across the range of values of *ϕ*. (B) Genotype–phenotype map for our mathematical model of pleiotropy. To keep things simple, we model 3 traits: a terminal cooperative trait, $z_{u}^{c}$ (blue circles), a terminal private trait, $z_{v}^{c}$ (red circles), and a pleiotropic regulation trait, $z_{p}^{c}$ (yellow circles). All traits in our model can be either active (coloured as above) or inactive (grey circles). This means there are 8 possible genotypes, each labelled *g*_*i*_, where *i*∈{1,2,…,8}. Although pleiotropy can be active or inactive for a given genotype, we use a parameter *ϕ*∈[0,1] to tune the strength of its effect. This allows us to use our simple genotype–phenotype map to model biological scenarios when pleiotropy is expected to be weak as well as strong. Furthermore, we assume that pleiotropy only has a functional effect when both other traits are active. Thus, although active pleiotropy is present in genotypes *g*_2_, *g*_4_, *g*_6_, and *g*_8_, we assume it only affects genotype *g*_8_, where it influences the probability, *ϕ*, that a *g*_8_ cell experiences a pleiotropic mutation given that a loss-of-function mutation has deactivated one of its other traits.

Regulatory networks, and the maps from genotype to phenotype, are often complex [31]. Evolutionary models of cooperation typically overlook this complexity and instead study optimal trait values, an approach known as the phenotypic gambit [32]. Here, we treat genetic architecture as a trait, like any other, that can itself evolve in response to natural selection [26,29]. We do this with a simplified model of pleiotropy. The definition of pleiotropy can vary between disciplines and authors [29,33–35], and here we mean the commonly used definition: Pleiotropy is when a single locus affects 2 or more traits [24]. Specifically, our model captures how mutation at a given locus affects 1 cooperative and 1 private trait (Fig 2). While there are a vast range of possible regulatory networks that might influence any 2 traits of interest, the impacts of pleiotropy can be captured by a single value *ϕ*, which is the probability that a mutation in a network with an active, cooperative, and private trait will give rise to a pleiotropic effect (Fig 2A).

There are, therefore, 3 traits in our model: a cooperative trait, a private trait, and a pleiotropy trait, which gives rise to 8 possible genotypes (Fig 2B). The goal of our model is to explore which of these 8 genotypes is favoured by natural selection in the long term due to competition among cells within and between groups. For a given cell, each of the traits is in either an active or inactive state. At the heart of the model is a tension between selection for cooperation within and between groups, i.e., the cooperative trait decreases a cell’s relative division rate within a group but brings benefits to the group function as a whole. This trait might, for example, represent a secreted enzyme that helps nutrient acquisition in a microbial group, or the suppression of cell proliferation in a simple multicellular organism to ensure proper functioning [18]. By contrast, the private trait simply increases a cell’s survival rate within its group. This trait might represent an enzyme involved in central metabolism, for example. With pleiotropy, mutations that ablate the cooperative trait increase the probability that the private trait is also lost and vice versa [29], where *ϕ* determines this probability (Fig 2A). A mutation matrix specifies the transition probabilities between all 8 genotypes in the model as a function of the strength of pleiotropy (see Methods).

Under a null model in which pleiotropy does nothing, mutations affect each trait independently, where mutations that cause loss-of-function in a trait occur with rate *μ*, and gain-of-function trait mutations occur with rate *νμ*, where *ν*<1. This value reflects the fact that it is typically easier to break trait functionality than to restore or create it, and we typically take *ν* = 0.01 to capture the strength of this bias. We use *μ* = 0.0001 per generation for the base mutation rate in most analyses, which describes the probability that a trait is mutated—and function is lost—per cell division. This value is expected to vary widely between systems and traits and is intended only as an illustration. We later perform parameter sweeps of both *μ* and *ν* across several orders of magnitude.

**Fig 3:**
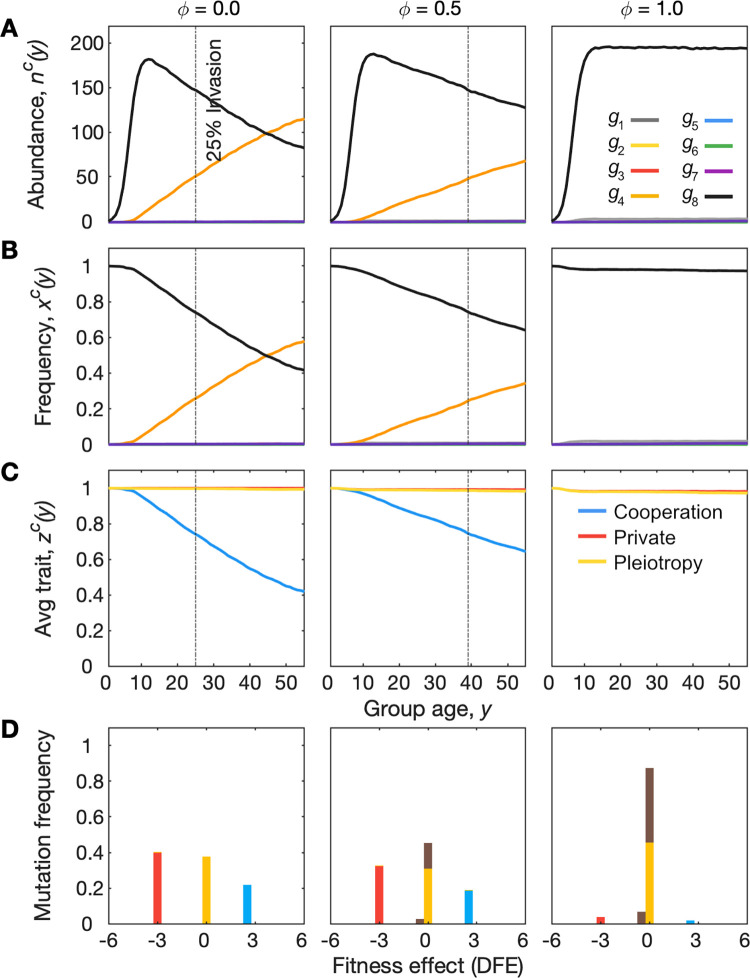
The evolution of cooperation within groups depends on the strength of pleiotropy *ϕ*. Within-group mutation selection dynamics are shown for a group founded by a cell with genotype *g*_8_, which actively expresses a cooperative trait, $z_{u}^{c}=1$, a private trait, $z_{v}^{c}=1$, and a pleiotropy trait, $z_{p}^{c}=1$. Growth of the group as its age, *y*, increases, is logistic, with a carrying capacity *K* = 200 at which point cells continue to divide, die, and turnover (see Methods). Dynamics are shown from left to right for 3 different strengths of pleiotropy, *ϕ*, where pleiotropy is absent/has no effect in the left-hand side column (*ϕ* = 0). For comparison, the vertical dashed line in (A-C) shows the point at which noncooperative lineages shown in orange make up 25% of the group. Pleiotropy leads to this point being delayed (*ϕ* = 0.5) or prevented (*ϕ* = 1). (A) Changes in genotype abundances, *n*^*c*^(*y*). (B) Changes in genotype relative frequencies, *x*^*c*^(*y*). (C) Changes in the average levels of cooperation, private trait expression, and pleiotropy, ${\bar{z}}^{c}(y)$. (D) Distribution of fitness effects: Shown is the effect on within-group fitness of the different types of loss-of-function mutations that occur, which are coloured by their phenotypic effect, where blue is loss of cooperative trait, red is loss of the private trait, yellow is loss of pleiotropy, and brown is loss of both cooperation and private trait (due to pleiotropy). When pleiotropy is weak or absent, loss-of-function mutations to cooperative traits increase the fitness of cells within the group, and loss-of-function mutations to private traits decrease the fitness of cells within the group. When the strength of pleiotropy is increased, mutations to either trait tend to have pleiotropic effects, which cancel one another out, meaning mutant cell lineages no longer gain an advantage within the group. Formally, the fitness effect is $DFE=ln(b_{mut}^{c}/b_{wt}^{c})-ln(d_{mut}^{c}/d_{wt}^{c})$, where $b_{mut}^{c}$ and $b_{wt}^{c}$ are the within-group birth rates of the mutant descendant and ancestral wild type, respectively, and $d_{mut}^{c}$ and $d_{wt}^{c}$ are the death rates of the mutant descendant and ancestral wild type, respectively. Parameters: *s*^*c*^ = *s*^*g*^ = 0.95; *K* = 200; *μ* = 0.0001; *ν* = 0.01. The code required to generate this figure can be found at https://github.com/euler-mab/pleiotropy and https://zenodo.org/record/6367788#.YjSBVurP2Uk.

We begin by following the evolutionary dynamics within a group. In our first model, each group is founded by a single cell, which gives 8 possible group types corresponding to the 8 cell genotypes (Fig 2B). While groups all start their life with clonal expansion of their founder, mutation–selection processes mean that their genotypic composition may change through time as they age. We can describe this process for each of the 8 group types. The dynamics for groups founded by genotypes 1 to 7 are shown in the Supporting information (S1–S7 Figs), and we focus here on genotype 8 groups (Fig 2B), hereafter referred to as “pleiotropic cooperators,” because they capture the effects of pleiotropy on cooperation (Fig 3). Groups with these genotypes initially grow towards their carrying capacity by clonal expansion but, depending on the strength of pleiotropy, have the potential to be invaded by cheater lineages that lack the cooperative trait but express the private trait (genotype 4; see Fig 2A). Importantly, we see that the extent and rate of invasion of the cheater lineage is diminished as the strength of pleiotropy, *ϕ*, is increased. Cheater lineages make up 25% of the group by approximately day 25 in groups without pleiotropy, by day 40 in groups with intermediate pleiotropy, and never (not before 50 days) in groups with strong pleiotropy (Fig 3B).

The resistance to invasion by cheater lineages occurs because pleiotropy reduces the frequency with which mutations give rise to a cheater phenotype. As a result, pleiotropy is able to increase the level of cooperation in groups (Fig 3C). The distribution of fitness effects (DFE) of loss-of-function mutations helps to show why pleiotropy is an effective mechanism for limiting cheater cell lineages within a given group (Fig 3D), something also clear from the dos Santos study [29]. When the strength of pleiotropy is relatively weak, mutations to the cooperative trait frequently give rise to mutant descendants that have a competitive advantage over the cooperative cells within the group. By contrast, when the strength of pleiotropy is relatively strong, mutations tend to have have neutral or deleterious effects on cells because a loss of cooperation also comes with a loss of the private trait. In the model, we assume that the effects of expressing the cooperative and private trait on within-group fitness are equal and opposite in magnitude, which is what leads to neutrality when both are lost. Some examples suggest that the loss of a private trait may have a stronger negative effect, such as cell death via apoptosis [36–38]. Such examples may lead to a negative change in within-group fitness when both traits are lost. We do not consider this case explicitly here, but it is only expected to strengthen the ability of pleiotropy to remove potential cheater lineages and thereby improve group function.

### Pleiotropy evolves to suppress cheater lineages and promote cooperation

Our within-group model supports the established, and intuitive, argument that pleiotropic links between a cooperative and private trait will help to maintain cooperation [21–25]. However, this model simply assumes that this pleiotropy exists, rather than explaining how it evolved. One origin of pleiotropy is a result of natural selection on traits unrelated to cooperation [25,39]. Pleiotropy is extremely common in all genotype to phenotype maps, whether or not cooperative traits are involved. As a result, cooperation may become pleiotropically linked to private traits simply through the way that regulatory networks normally evolve. A more intriguing alternative is that pleiotropy evolves to promote cooperation [25,31]. However, as discussed above, a recent theoretical treatment of this idea argued that, despite the abilitiy of pleiotropy to decrease the cheater load within groups, it will not generally evolve to promote cooperation over evolutionary time [29]. Specifically, this earlier study found that “*(1) pleiotropy does not stabilise cooperation*, *unless the cooperative and private traits are linked via a genetic architecture that cannot evolve (mutational constraint); (2) if the genetic architecture is constrained in this way*, *then pleiotropy favours any type of trait and not especially cooperation; (3) if the genetic architecture can evolve*, *then pleiotropy does not favour cooperation; and (4) there are several alternative explanations for why traits may be linked*, *and causality can even be predicted in the opposite direction*, *with cooperation favouring pleiotropy*.”

The authors did find that pleiotropy will often help reduce the prevalence of cheaters within certain groups, in line with experimental evidence in microbes [21–23]. However, they also found that between-group selection was not strong enough to notably increase cooperation across the whole population over evolutionary time (Figs S14 and S16 in [29] show a small increase in evolved cooperation with pleiotropy). We discuss this study in detail in the Supporting information (S1 Text), where we conclude that a different type of model will benefit the study of the evolution of cooperation and pleiotropy.

We developed our model, therefore, to study whether pleiotropy will evolve as a mechanism to promote cooperation within multicellular groups over evolutionary time. To do this, we extend the model to capture how groups perform, and compete, across a wider population. In each group, within-group evolution occurs as just discussed (Fig 3), which, in turn, affects a group phenotypic trait, which we call the group’s “function.” For example, this might represent the ability of a bacterial strain to produce a protective biofilm, or the ability of a multicellular organism to coordinate its development. The key is that the group function is assumed to breakdown when within-group selection dynamics take hold.

We capture group function at age *y* by $z_{f}^{g}(y)={\bar{z}}_{u}^{c}(y){\bar{z}}_{v}^{c}(y)$, where ${\bar{z}}_{u}^{c}(y)$ is the average amount of cooperation in the group, and ${\bar{z}}_{v}^{c}(y)$ is the average expression of the private trait (see Methods for more details). Thus, we assume that cells expressing both cooperative and private traits contribute fully to group function, whereas cells with either trait missing do not. Specifically, cells lacking only cooperation behave as cheater lineages, as just discussed, while cells lacking the private trait function poorly. As a result, groups founded by these cells (genotypes 1 to 6; Fig 2B) have a greater probability of extinction than higher functioning groups (genotypes 7 and 8; Fig 2B). But within-group evolutionary dynamics also means that group function can degrade in groups founded by cooperative cells (Fig 3).

We study the evolution of these processes with a system of PDEs, which captures an infinitely large age-structured population of groups. We start with a population dominated by individuals lacking all traits (genotype 1) and allow them to evolve by numerically integrating within-group and between-group dynamics over time until a stable age distribution is reached. We then ask if pleiotropy evolves as a function of 3 key parameters: the strength of pleiotropy, *ϕ*, expected group life span, *λ*, and group size at maturity, *K*.

A common approach to model multilevel selection in evolutionary biology is the haystack model [40], which is the approach used in [29]. In the haystack model, group selection is modelled implicitly as an emergent process resulting from fitness differences between individuals within groups; there is no explicit consideration of group-level survival and reproduction events. Instead, all group phenotypes are described in terms of the set of individual phenotypes. In contrast, the PDE approach we use models group-level phenotypes and considers their direct impact on group-level survival and reproduction events. This approach allows us to more explicitly capture microbial groups and multicellular organisms, which have group-level traits that are important for group fitness but are threatened by within group competition.

**Fig 4:**
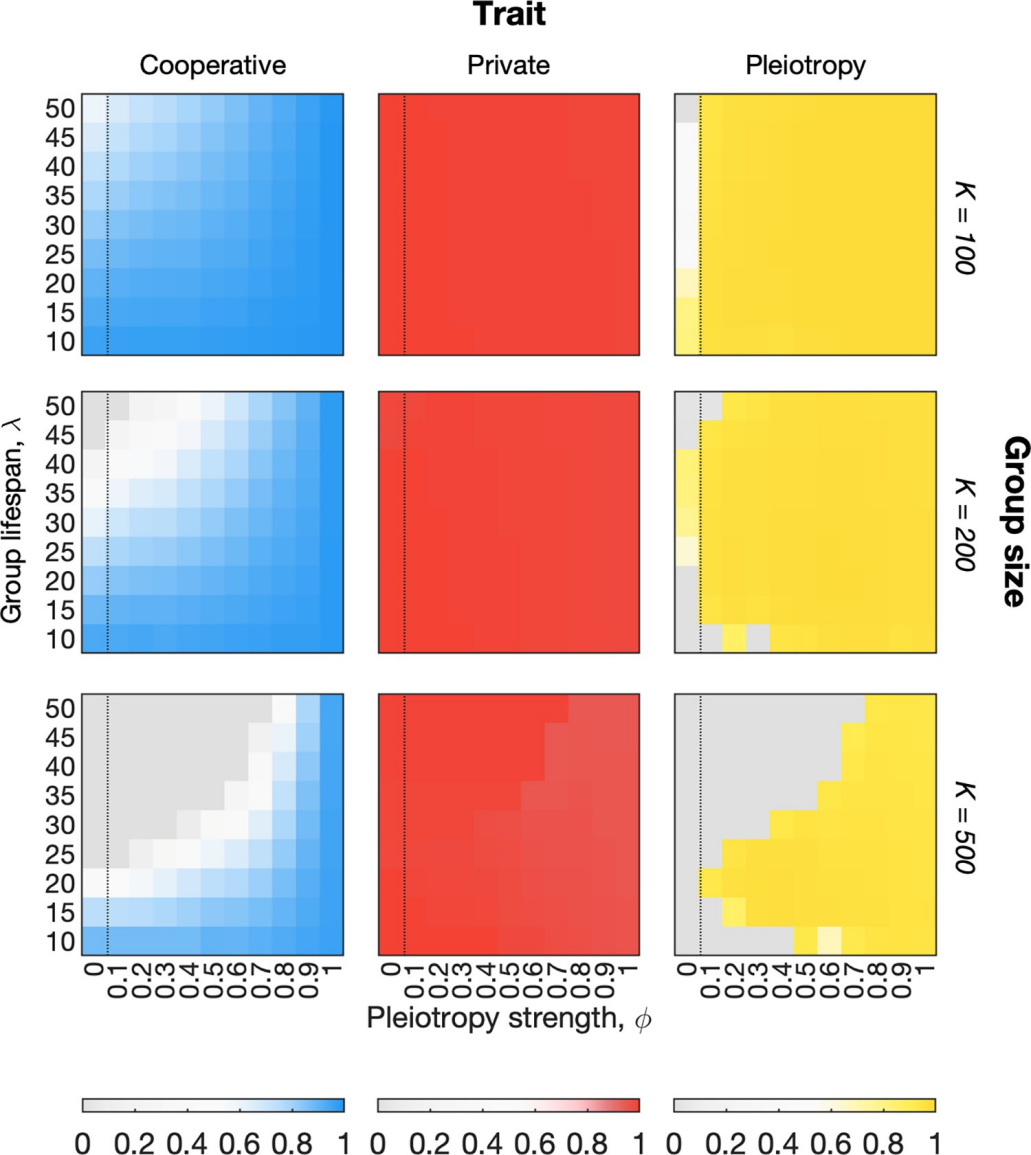
The evolution of pleiotropy promotes cooperation. Heatmaps show average trait values among the global population of cells (across all groups) at steady state in our model. Results are shown for 3 group sizes (increasing from top to bottom). Dotted line on each heatmap indicates separation between point where pleiotropy has no effect (control case *ϕ* = 0) to regions where it has a nonzero influence on the dynamics. Cooperation evolves for a wide range of parameters but is destabilised for longer group life spans, *λ*, and larger group sizes, *K*, due to the emergence of cancerous cell lineages. This effect is strongest without pleiotropy (*ϕ* = 0). When pleiotropy has an effect (*ϕ*>0), natural selection favours its evolution resulting in higher levels of cooperation, i.e., the blue intensity increases from left to right in the cooperation plot. As expected, pleiotropy is most favoured when it is more effective, i.e., the strength of pleiotropy, *ϕ*, is higher. Note that the level of expression of the pleiotropic trait can be nonzero even when it has no effect due to stochastic effects. In these situations, however, the expression of the pleiotropic trait does not influence cooperative evolution. We show below that pleiotropy will also evolve with a cost, which greatly reduces this stochasticity (S11 Fig). Parameters: *s*^*c*^ = *s*^*g*^ = 0.95; *K* = 200; *μ* = 0.0001; *ν* = 0.01. The code required to generate this figure can be found at https://github.com/euler-mab/pleiotropy and https://zenodo.org/record/6367788#.YjSBVurP2Uk.

Our analysis reveals a wide range of parameters where pleiotropy evolves and, in doing so, promotes cooperation over evolutionary time (Fig 4). In particular, when the effects of pleiotropy are absent in the model (*ϕ* = 0), the levels of cooperation that evolve are often markedly decreased (Fig 4). These results includes cases where cooperation evolves to a lower level without pleiotropy but not to 0, which is also seen to a modest extent in the supplementary figures of dos Santos and colleagues [29]. We also see many cases where cooperation is negligible without pleiotropy, but present with pleiotropy, predicting that pleiotropy can enable the evolution of cooperation in regions where kin selection alone does not maintain it (Fig 4, S8 Fig). Moreover, we do not see the evolution of pleiotropy in a control model of 2 private traits (Methods; S9 Fig). These 2 contrasts—removing pleiotropy and removing cooperation—demonstrate that cooperation can depend on the evolution of pleiotropy and vice versa.

Our model predicts that pleiotropy is more important for cooperation as groups become larger and longer lived. This is because, if groups are small or short lived, there is less opportunity for cheater lineages to arise and interfere with group functioning (Fig 3). As expected, the strength of pleiotropy—how protective pleiotropy is against invasion of mutant cheater genotypes—is an important factor in determining when pleiotropy evolves (variation in the x-direction in Fig 4). Nevertheless, we find that pleiotropy evolves and increases cooperation even for low levels of protection (*ϕ*<0.5, blue plots, Fig 4). In some cases, pleiotropy evolves in the model when it has no phenotypic effect via genetic drift (Fig 4, *ϕ* = 0). As expected, this effect is strongest when group sizes are small and short lived because this is when natual selection is also generally weakest. Below, we introduce a cost to pleiotropy that largely removes this effect and shows it does not contribute to our findings.

Another assumption of potential importance is how detrimental the invasion of cheater lineages is to overall group function. If the invasion of even a few mutant cheaters can damage group function, then mechanisms that resist that invasion are likely to be more strongly favoured by between-group selection. To be conservative, we do not assume a high detrimental impact where only a few cheater cells are fatal for group functioning but instead consider a linear function where some multicellular function persists in the face of very large numbers of noncooperator cells. Specifically, recall that group function at age *y* is given by $z_{f}^{g}(y)={\bar{z}}_{u}^{c}(y){\bar{z}}_{v}^{c}(y)$, a function that declines linearly with the invasion of noncooperative mutants (all else being equal). However, even without a high detrimental impact associated with a small numbers of cheaters, we find that pleiotropy is important for cooperation in groups that generate only 5,000 to 10,000 cells across their life span (i.e., 5 × 10^3^ to 10^4^ cell divisions in total). This prediction is borne out in Fig 4 where the total number of cell divisions in the model corresponds roughly to group size multiplied by the number of generations in the life span (i.e., y-axis value multiplied by group size in Fig 4; see Methods). As discussed, these are only rough estimates as they depend on mutation rate and other assumptions that will vary between systems. However, given that many cellular groups undergo many more cell divisions than this estimate, these results predict that the evolution of pleiotropy has the potential for widespread impacts.

**Fig 5:**
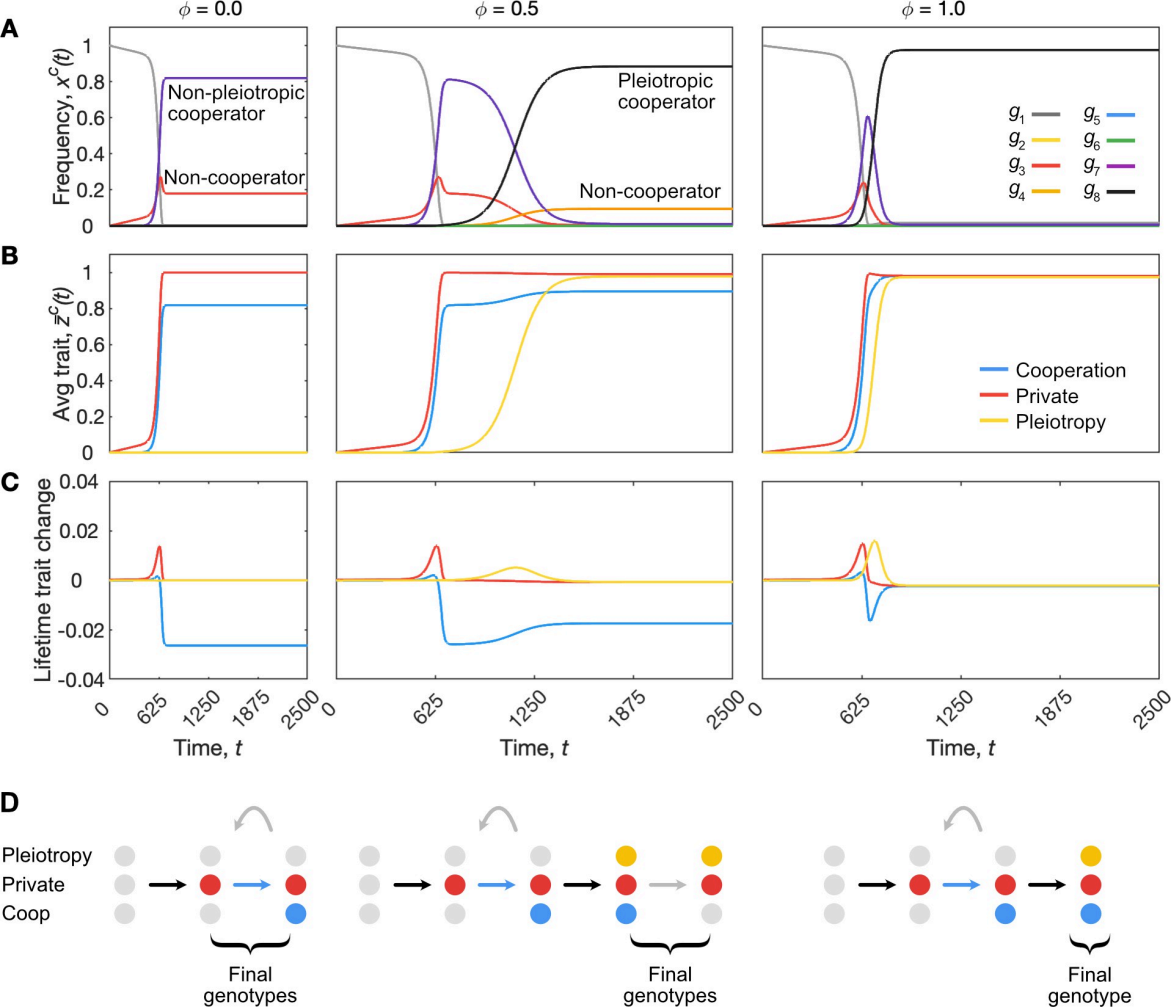
Long-term evolutionary dynamics of cooperation and pleiotropy. Dynamics are shown for the global population of cells over time, *t*, which encompasses many generations of cell groups. These dynamics encompass both within-group and between-group selection dynamics. We show 3 strengths of pleiotropy, *ϕ*, to capture 3 scenarios where stronger pleiotropy is associated with more rapid and complete evolution of both pleiotropy and cooperation. (A) Changes in global genotype relative frequencies, *x*^*c*^(*t*). (B) Changes in the global average levels of cooperation, private trait expression, and pleiotropy, ${\bar{z}}^{c}(t)$. (C) Average change in traits over a group lifetime, measured as the difference between the average trait values among groups aged *y* to those expected from their founding cell at birth. (D) Order in which genotypes invade in the global population, where a blue arrow indicates a gain of cooperation (via between-group selection), a grey arrow indicates a loss of cooperation (via within-group selection) and a black arrow indicates no change in cooperation. Parameters: *s*^*c*^ = *s*^*g*^ = 0.95; *K* = 200; *μ* = 0.0001; *ν* = 0.01; *λ* = 20. The code required to generate this figure can be found at https://github.com/euler-mab/pleiotropy and https://zenodo.org/record/6367788#.YjSBVurP2Uk.

Our model also allows us to follow the order in which the traits evolve in the population (Fig 5). Without pleiotropy (*ϕ* = 0), cooperative genotypes can rapidly evolve, but so too do noncooperative genotypes (red lines in Fig 5A). As a result, these cheater lineages end up making up a substantial proportion of the population such that cooperation and group functioning are limited (Fig 5B, left panel). By contrast, when pleiotropy can influence the distribution of mutational effects, we see that it evolves extremely rapidly after the origin of cooperation itself. Indeed, for the higher strengths of pleiotropy, it evolves alongside the initial origin of cooperation in the population (Fig 5B, middle panel).

Rather than being a secondary adaptation restricted to derived multicellular groups, therefore, our model predicts that pleiotropy can be important at the origin of cooperation within multicellular groups. One can also assess the effects of pleiotropy in terms of trait-based mutational load: the average trait values of a group at age *y* relative to their trait value at birth (Fig 5C). The mutational load is greatest for the cooperative trait, with groups tending to express reduced cooperation aged *y* than they do at their origin. However, this mutational load of cooperation is reduced with the evolution of stronger pleiotropy (Fig 5C). In this way, pleiotropy does not just act to reduce the emergence of cheaters in a given group (Fig 3), it acts across the whole population and can greatly increase the evolved level of cooperation (Figs 4 and 5).

### Pleiotropy evolves to stabilise cooperation across a wide range of conditions

We have so far assumed that groups are formed from a single cell. While this is realistic for the majority of multicellular organisms, other cellular groups, particularly microbial groups, commonly contain multiple genotypes that meet and mix. If large numbers of different genotypes meet and mix—and relatedness is close to 0—the evolution of pleiotropy and indeed cooperation does not occur in our model. Under these conditions, there is no between-group genetic variation and the outcome of competition is determined solely by within group dynamics (S10 Fig). Here, so long as genotypes that lack the cooperative phenotype can arise at some point, they will take over and pleiotropy serves no function. However, relatedness can often be relatively high in microbial groups due to spatial structure, where a patchwork of groups form, each dominated by a single genotype [1]. We can study the effects of an intermediate level of relatedness in our model by assuming groups are founded by 2 cells (chosen uniformly at random from their parent group), such that there are now up to 32 different group genotypes in the population. This case has an important difference to the single-cell bottleneck case where cheater cells always start a new group alone with little chance of survival. With 2 cells, cheater cells now have the chance of founding groups alongside cooperators that they can exploit, thus greatly improving their prospects. Despite the added complexity, we see again that the evolution of pleiotropy is often favoured and able to promote the evolution of cooperation as it evolves (S11 Fig). While the importance of pleiotropy in our model rests upon some relatedness between cells, therefore, it does not rest upon a single cell origin.

Our conclusions are also robust to changing other assumptions and parameters. One key consideration is that there may be a cost to pleiotropy if, for example, the regulation of 1 trait is compromised by its linkage to another [41]. However, we find that pleiotropy still evolves if it carries such costs to a group’s functioning (S12 Fig), which is further testament to its ultimate importance for improving group function. Another important parameter is the benefit of cooperation (strength of group selection). Reducing the benefit of cooperation in our model reduces the scope for the evolution of cooperation but, importantly, where cooperation can evolve there are broad parameter ranges where pleiotropy evolves to increase cooperation (S13 Fig). Notably, the evolution of pleiotropy is even seen when natural selection for cooperation is very weak, as may have occurred at the inception of multicellular life.

Varying the relative probability of gain-of-function mutations has little impact on outcomes (S14 Fig). However, as expected, the baseline mutation rate is important. Increasing this mutation rate causes the more rapid breakdown of cooperation, which requires stronger pleiotropic effects for cooperation to be maintained. However, so long as strong pleiotropic links are possible, we see that they rapidly evolve and again stabilise cooperation (S15 Fig). For reduced mutation rates, cheater lineages arise less often and so, even in the absence of pleiotropy, cooperation can be maintained more easily. All else being equal, therefore, pleiotropy will now only evolve in larger or longer-lived cellular groups. For example, halving the mutation rate (*μ* = 0.00005) roughly doubles the number cell divisions where pleiotropy becomes critical for cooperation (compare Fig 4 with S16 Fig). However, even if we lower the mutation rate an order of magnitude (*μ* = 0.00001), we still observe the widespread evolution of pleiotropy in groups of only 10,000 to 20,000 cell divisions (S16 and S17 Figs show this effect, with and without a cost to pleiotropy, respectively). In summary, we observe that the evolution of pleiotropy promotes cooperation for relatively small multicellular groups across a wide range of parameters.

### Pleiotropy is predicted to be important in a simple model of multicellular organisms

Our modelling assumptions are most suited to multicellular groups of microbes and probably some of the ancestral organisms that gave rise to multicellularity in the algae, plants, animals, and fungi. With the evolution of derived multicellularity came many complexities, which our models do not capture. Central among these is the importance of the germ and soma separation for modern multicellular life. We, therefore, next ask whether this biology influences the evolution of pleiotropy and multicellular cooperation.

**Fig 6:**
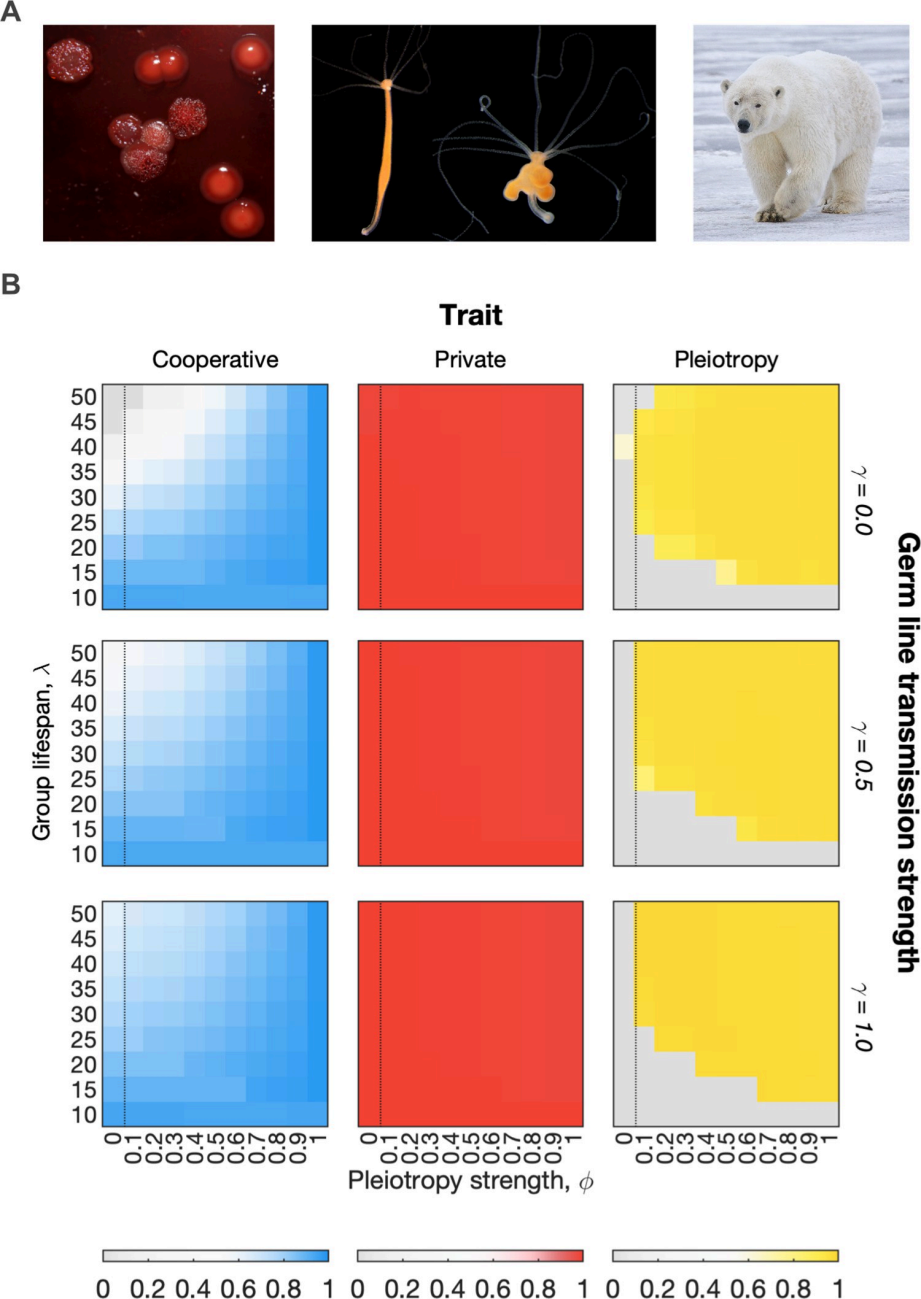
The evolution of pleiotropy in multicellular groups with a germ line. (A) Cheater lineages that threaten cooperation evolve in multicellular species with a germ line, as well as in simpler multicellular groups. In multicellular organisms, these lineages manifest themselves as tumours and cancers that emerge by mutation. From left to right, bacteria, simple and complex animals. Bacteria: Spontaneous *lasR* mutant emerge during evolution of the pathogen *Pseudomonas aeruginosa*. The mutants do not contribute to the production of public goods molecules that their wild-type counterparts do, which enables them to gain a short-term competitive advantage (*Image credit*: *Sheyda Azimi and Steve Diggle*). Simple animal: Spontaneous tumour formation in the basal metazoan Hydra, a tumour-bearing *Hydra oligactis* polyp (right) is shown next to a healthy animal (left). *Credit*: *Alexander Klimovich*, *Kiel University*. Complex animal: Cancer is widespread in long-lived animals, especially zoo animals where other selection pressures are minimised. Nanuq, a 29-year-old polar bear, died from liver cancer in The Columbus Zoo, Ohio, in 2017 (image shows a different polar bear; *credit*: *Alan D*. *Wilson*). (B) To study the impact of pleiotropy on multicellular groups with a germ line, we varied *γ*, a measure of the strength of the transmission of the germline, modelled as the likelihood that a group propagates the genotype of its founding cell at the age at which it reproduces versus a cell selected at random. Heatmaps show average trait values among the global population of cells (across all groups) at steady state in our model. Results are shown for 3 germ line strength parameters (increasing the strength of the germline transmission between ancestor and descendent groups from top to bottom). Increasing the strength of the germline has a positive effect on the evolution of cooperation, but pleiotropy continues to be favoured in regions of the parameter space in which cooperation is vulnerable to breakdown. The dotted line marks the boundary between pleiotropy having no effect (control case) and pleiotropy having an effect on the outcome of mutations. Parameters: *s*^*c*^ = *s*^*g*^ = 0.95; *K* = 200; *μ* = 0.0001; *ν* = 0.01; *ζ* = 0.02. The code required to generate this figure can be found at https://github.com/euler-mab/pleiotropy and https://zenodo.org/record/6367788#.YjSBVurP2Uk.

Here, we assume that multicellular groups can influence which genotype they transmit during reproduction via a germ line that undergoes fewer cell divisions, and mutations, than the soma. Specifically, with probability *γ*, an ancestral group seeds a new group with a cell of the same genotype to its founding cell, and with probability 1−*γ*, a cell is chosen uniformly at random from the group to seed a new descendant (as before). As discussed above, we also assume a cost to pleiotropy to be conservative. The model predicts, as expected, that a germ line is generally benefitial for the evolution of cooperation, as it reduces the chance that noncooperative genotypes will start new groups. Nevertheless, with a germ line, we still see the widespread evolution of pleiotropy (Fig 6).

**Fig 7:**
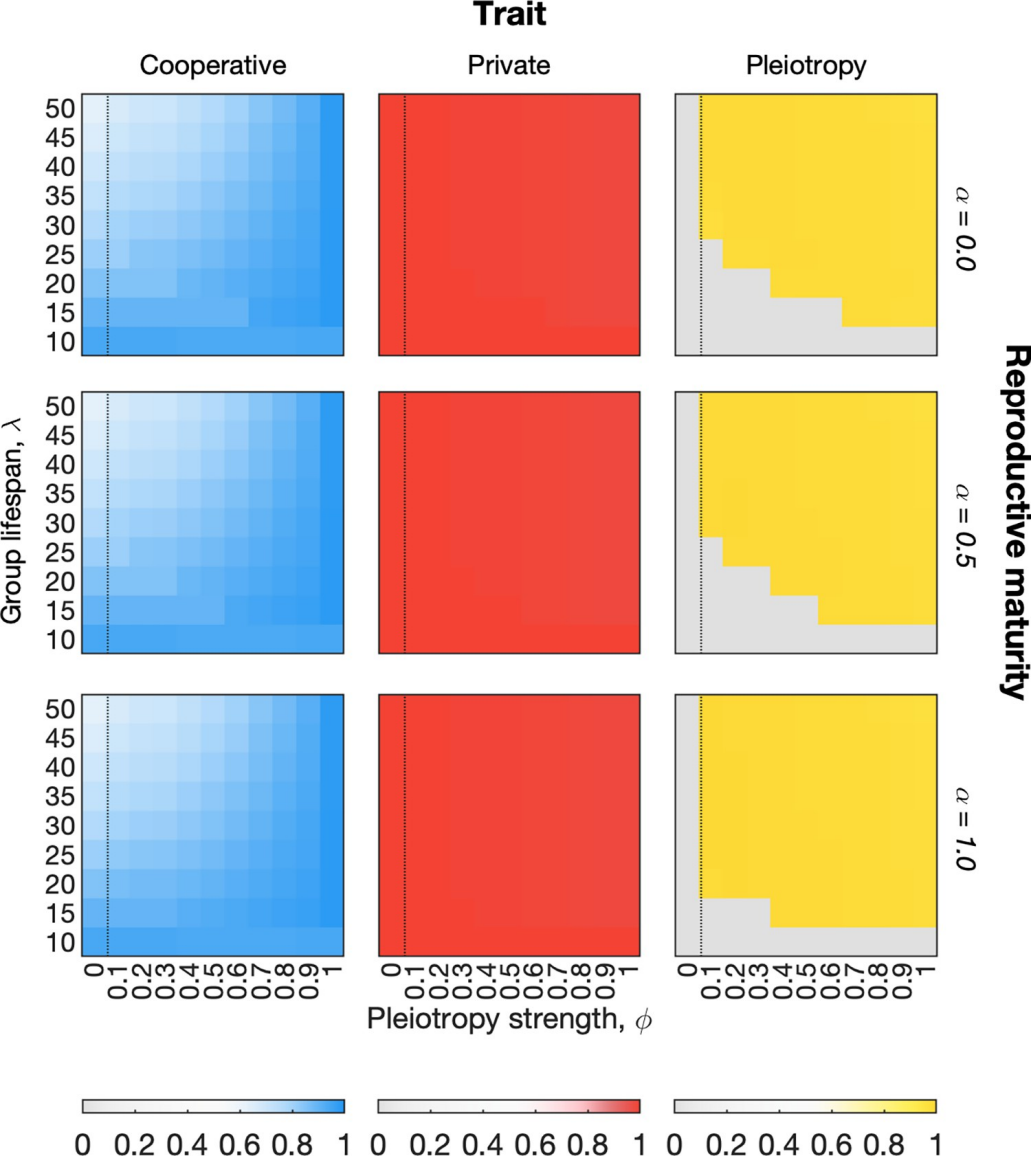
The evolution of pleiotropy in multicellular groups with a germ line and delayed reproductive maturity. We varied *α*, the fraction of the expected life span *λ* groups must have aged to before they can reproduce. We also assumed a germ line, *γ* = 1, and that the evolution of pleiotropy carries a cost. Heatmaps show average trait values among the global population of cells (across all groups) at steady state in our model. Results are shown for 3 reproductive maturity parameters (increasing the age at which maturity is reached from top to bottom). Increasing the age of reproductive maturity favours the evolution of pleiotropy. The dotted line marks the boundary between pleiotropy having no effect (control case) and pleiotropy having an effect on the outcome of mutations. Parameters: *s*^*c*^ = *s*^*g*^ = 0.95; *K* = 200; *μ* = 0.0001; *ν* = 0.01; *ζ* = 0.02; *γ* = 1. The code required to generate this figure can be found at https://github.com/euler-mab/pleiotropy and https://zenodo.org/record/6367788#.YjSBVurP2Uk.

A second important characteristic of derived multicellular organisms, like humans, is the need to reach a certain age before reproduction is possible. Up until now, reproduction among our multicellular groups has been age independent. We therefore introduce another parameter, *α*, which determines the fraction of the expected life span that groups must reach before they can reproduce. For example, if the expected life span is *λ* = 50 and *α* = 0.5, then groups can only reproduce after age *y* = 25. On its own, adding this requirement reduces the levels of cooperation in the population, because it means that organisms reproduce when they have the highest levels of cheater mutations (S18 Fig). However, the negative effects of late life reproduction on cooperation are reduced if we assume that these organisms also have a germ line (Fig 7). Moreover, this effect rests upon the ability of an organism to evolve effective pleiotropic constraints (*ϕ* > 0). We find, therefore, that organisms with a germ line and delayed reproduction will evolve pleiotropy to protect against cheater lineages and ensure high-level functioning when they reproduce (Fig 7C).

## Discussion

Our models predict that pleiotropy can greatly increase the levels of cooperation within multicellular groups (Fig 4 and S11–S18 Figs). Natural selection favours pleiotropy because it reduces the probability that cheater lineages arise within a cellular group or multicellular organism, which would otherwise damage functioning (Fig 3). We expect this process to be important whenever groups become large or old enough for the emergence and spread of cheater lineages to cause significant harm to multicellular function (Fig 4). These conditions are expected in relatively small and simple multicellular groups. We find pleiotropy can evolve to suppress cheater lineages in groups that undergo as few as 10^4^ cell divisions.

This 10^4^ cell divisions estimate depends on parameters, most notably mutation rate. Mutation rates vary widely, but to give an idea of natural rates, a simple animal like *Hydra vulgaris* would be expected to have between roughly 1 and 100 mutations per genome per cell division, based upon its approximately 1 Gbp genome and recent estimates of somatic mutation rates taken from mammals (10^−9^–10^−7^ mutations per basepair per cell division) [42,43]. If we divide these mutation rates by the number of genes in *H*. *vulgaris* (20,000), this gives rates between one-half and 50 times our standard value of *μ* (0.00005 to 0.005). For a trait affected by a single gene, this range may still overestimate the rate of loss-of-function mutations, because not all mutations in a gene will hit a coding region nor lead to a loss of function if they do. However, the traits we are interested in here—for example, regulated cell proliferation—are often affected by large networks of genes, such that mutations in many genes have the potential to influence the trait, for example, drive unregulated cell proliferation. Our standard value, therefore, appears to be reasonable, but in species with lower mutations rates, a larger number of cell divisions are required before pleiotropy is needed to maintain cooperation (S16–S17 Figs). However, even if 10^4^ cell divisions is an underestimate by 1 or even several orders of magnitude, many multicellular groups will remain above the threshold at which pleiotropy is predicted to be important. There are an estimated 10^5^ cells in hydra [44], 10^5^ neurons in the *Drosophila* brain [45], 10^10^ cells in a bacterial colony [46], and 10^13^ cells in humans [47], where these large standing cell numbers can still greatly underestimate total cell divisions because of cell turnover [30]. If relatively few cell divisions are needed for the evolution of pleiotropy, this suggests that pleiotropy may have had an important role in cheater suppression at the origins of multicellularity. Further consistent with this hypothesis, the evolutionary dynamics in the model predict that pleiotropy will evolve rapidly, close to simulatenously, with the first evolution of cooperation (Fig 5).

We have identied a scenario where pleiotropy evolves because of its positive effects on cooperation, but pleiotropy can also arise for other reasons [25,35,39,48]. Pleiotropy is widespread for all types of traits—cooperative and noncooperative—and can readily arise, for example, as a way to coregulate multiple traits [39,48]. Such regulatory structures might help to stabilise a cooperative trait or help cooperation to first arise when there is positive frequency-dependent selection [49]. A challenge for the future is to distinguish between examples where the effects of pleiotropy on cooperation arose independently of cooperation, and cases where cooperation drove the evolution of pleiotropy. Our within-group model underlines that, no matter why pleiotropy first evolves, it has the potential to promote cooperation (Fig 3). Moreover, when individuals are related, this can generate between-group selection for pleiotropy and the stabilisation of cooperation in the long term (Figs 4 and 5).

There are other evolutionary processes than those we have modelled here that may lead to cooperation being associated with pleiotropy. Whenever cooperation is linked to the ability of a species to compete and persist in an ecosystem [50], for example, species-level selection may enrich for species that have evolved pleiotropic links that promote cooperation over those that do not [51]. Another potential route to pleiotropy is when 1 individual evolves to enforce cooperation in another. For enforcement to be effective, the enforcer needs to find a way to constrain the recipient in some way from escaping the enforcement. This need can result, therefore, in a pleiotropic link in a recipient where the cessation of cooperation is linked to a personal cost from enforcement [25,28]. This case is notable in that it does not rest on there also being positive relatedness between interacting individuals (cf., S10 Fig).

Enforcement may be important in microbial examples of pleiotropy, including the case of *Dictyostelium discoideum* [24] where enforcement of 1 cell type by another appears to be important for cooperation [52]. However, our modelling also suggests that pleiotropy may evolve in microbes to limit the spread of cheater lineages. Mutation rates can be relatively low in microbes [53], and the arrival and mixing of multiple genotypes within a microbial group has the potential to both undermine the evolution of cooperation and the potential for pleiotropy with it [29]. However, cooperative traits are known to be widespread in microbes, particularly in the well-studied bacteria [54]. Moreover, groups of bacteria reach vast numbers and the evolution of noncooperator lineages has been observed [21,55,56]. Whenever these lineages cause significant harm to cooperative function, therefore, there is the potential for pleiotropy to evolve to prevent their emergence.

We predict that the evolution of pleiotropy will be particularly important for multicellular organisms. Development from a single cell (Fig 4 versus S11 Fig), combined with relatively high somatic mutation rates (S15–S17 Figs) and the potential for a very large numbers of cell divisions (Fig 4 and S16 and S17 Figs), are all predicted to favour pleiotropy as a means to promote cooperation. Consistent with this prediction, many multicellular organisms suffer from cancer, which has long been viewed as analogous to the evolution of cheater lineages within cellular groups [29,31,57–61]. Moreover, the study of cancer has identified powerful mechanisms to supress potential cheater lineages, which function by linking oncogenic traits (loss of cooperation) to secondary traits that limit viability (loss of private trait) [31,38,62,63]. For example, loss of function mutations in the retinoblastoma tumour suppressor gene are linked to the activation of programmed cell death (apoptosis) [36,37], while activation of ras genes—key regulators of cell proliferation—are associated with cellular senescence and cell cycle arrest [64]. Other mechanisms push cells with oncogenic mutations to differentiate from a proliferative cell type (stem cell) to one that does not divide [63]. The links between cooperation and cell viability can be cell intrinsic but they also occur via the actions of immune cells and othe cell types [62,65], which can promote apoptosis in a mutant cell [66,67] or even physically force it out of a proliferative tissue [68]. The biology of multicellularity, therefore, appears to be characterised by a large number of pleiotropic connections between abberant cell proliferation that threatens cooperation and the loss of traits that cells need to survive and divide.

The effects of pleiotropy in our models is testament to the importance of considering molecular mechanisms in the study of cooperation, particularly in cellular systems [31]. In microbes, for example, mechanisms such as prudent regulation of cooperative traits, quorum sensing, and green beard genes can all serve to promote cooperation [69–71]. High relatedness is also clearly important for cooperation across a range of cooperative systems, and is particularly important for cooperation in cellular groups. Experiments have demonstrated how relatedness promotes cooperation in microbes, and development from a single cell was likely to have been central to the evolution of complex multicellularity [14,15]. However, the large numbers of cell divisions that occur in many cellular groups means that single-cell ancestry is far from sufficient to maintain cooperation. Indeed, our models predict that even relatively small and simple cellular groups will suffer if cheater lineages are left unchecked. This suggests that, in addition to the single-cell bottleneck, the evolution of pleiotropic constraints may have been important for the origins of multicellularity and the subsequent major transition in evolution that led to the complex multicellular life that we see today.

## Methods

### A general model of multilevel selection using an age-structured model

Capturing the full dynamics of a multilevel selection process is difficult because the potential for selection within and between groups creates an enormous space of possible outcomes. For these reasons, many early models of multilevel selection, such as the haystack model [40], attempted to simplify the problem by neglecting to model group-level events explicitly. While this simplifies the model, the approach misses the fact that group-level events and individual-level events can happen on different timescales, something that is particularly important for capturing the biology of most multicellular groups.

To capture the 2 levels of selection explicitly, we study the dynamics of natural selection in an age-structured population of multicellular groups and in subpopulations of cells within those groups. Changes in the distribution of groups of different ages and types evolves with time *t* and age *y*. Changes in the distribution of cells of different types within each group evolves with age *y*. Using a similar approach to [72], we use a system of PDEs to model changes in the age-structured population of different types of groups over time. We model changes in abundances of different cell types within the different group types as they age using stochastic simulations. These simulations then allow us to characterise and describe how groups of different types differ in their development as they age. Within-group differences in development further provide the basis for differences in reproductive success between groups founded by different types of cells. All the code used to perform our numerical analyses is open source and can be found in GitHub (https://github.com/euler-mab/pleiotropy) and Zenodo (https://zenodo.org/record/6367788#.YjSBVurP2Uk).

We begin by deriving and describing the structure of our model in general terms, before turning to the specifics of how we use it to study the evolution of cooperation and pleiotropy.

### Cell and group types

We assume there is finite set of cell types, *i*,*j*∈*I*, and a finite set of group types, *k*,*l*∈*K*. We refer to cells of type *i* as *i*-type cells and multicellular groups of type *k* as *k*-type groups. To distinguish between other group-level and cell-level variables in our model, we use superscripts *g* and *c*, respectively. The type of a group is assumed to be defined by the type(s) of its founding cells. In our standard model, we assume that a group is founded by a single cell. In this case, the set of cell genotypes and group genotypes is the same (i.e., *I* = *K*). In a later model, we assume that a group is founded by 2 cells. In this case, the set of group types contains all unordered pairs of cell types, and the index notation becomes more cumbersome, although all the same principles for the model hold true. We therefore focus our description here on the simpler model. Let us now describe how the abundances of cells and groups changes over time.

### Between-group dynamics

We begin with the group population. In the limit as the population of groups gets large, we assume that relative density of *k*-type groups aged *y* can be modelled as a continuous quantity, $n_{k}^{g}(t,y)\in [0,1]$. In the absence of births and deaths of whole groups, all groups in the population simply age. What this means is that if there were a population of 10 groups at time *t* aged *y* = 1, then at time *t*+10, there will be 0 groups aged *y* = 1, and 10 groups aged *y* = 11.

Consequently, the relative density of *k*-type groups aged *y* at time *t* changes over time according to a system of PDEs satisfying a conservation law of the form

$$\frac{\partial n_{k}^{g}}{\partial t}(t,y)+\frac{\partial n_{k}^{g}}{\partial y}(t,y)=0,$$ (1)

where there is no change in the overall density of groups in the population. This law can be derived as follows. First, note that within a particular age range [*y*_1_, *y*_2_], the total abundance of *k*-type groups aged *y* at time *t* is given by

$$\int_{y_{1}}^{y_{2}}n_{k}^{g}(t,y)dy.$$ (2)

If we assume that there are no births or deaths of groups within the age range, then the abundance of individuals in the age range [*y*_1_, *y*_2_] can only change because of a process of ageing. Groups of a younger age may enter this age range at the lower age *y*_1_, and those within the age range may get older than the upper age bound *y*_2_. If we suppose that groups age at a constant rate *v*, then the rate of change of *k*-type groups aged *y* at time *t* is just $vn_{k}^{g}(y,t)$. The rate of change of the total abundance of individuals at time *t* in the age range [*y*_1_, *y*_2_] is given by

$$\frac{d}{dt}\int_{y_{1}}^{y_{2}}n_{k}^{g}(t,y)dy=v\left(n_{k}^{g}({t,y}_{1})-n_{k}^{g}({t,y}_{2})\right),$$ (3)

where it is equal to the flux of the ageing population over the boundaries of the age range. Let us now integrate both sides of this equation to get an expression for the abundance of *k*-type groups in [*y*_1_, *y*_2_] at time *t*_2_>*t*_1_ in terms of the abundance of *k*-type groups aged *y* at time *t*_1_ and the total flux at each boundary during this time period. We have

$$\int_{y_{1}}^{y_{2}}(n_{k}^{g}(t_{2},y)-n_{k}^{g}(t_{1},y))dy=-v\int_{t_{1}}^{t_{2}}(n_{k}^{g}({t,y}_{2})-n_{k}^{g}(t,y_{1}))dt.$$ (4)

Assuming $n_{k}^{g}(t,y)$ is differentiable, then we can use the fundamental theorem of calculus to rewrite the integrands on both sides as

$$n_{k}^{g}(t_{2},y)-n_{k}^{g}(t_{1},y)=\frac{d}{dt}\int_{t_{1}}^{t_{2}}n_{k}^{g}(t,y)dt=\int_{t_{1}}^{t_{2}}\frac{\partial }{\partial t}n_{k}^{g}(t,y)dt,$$ (5)

and

$$n_{k}^{g}(t,y_{2})-n_{k}^{g}({t,y}_{1})=\frac{d}{dy}\int_{y_{1}}^{y_{2}}n_{k}^{g}(t,y)dy=\int_{y_{1}}^{y_{2}}\frac{\partial }{\partial y}n_{k}^{g}(t,y)dy,$$ (6)

respectively, where we have used Leibniz integral rule to take the derivative operators inside the integrals on the right-hand side. Substituting back in to Eq (4) and rearranging, we have

$$\int_{y_{1}}^{y_{2}}\int_{t_{1}}^{t_{2}}(\frac{\partial }{\partial t}n_{k}^{g}(t,y)+v\frac{\partial }{\partial y}n_{k}^{g}(t,y))dt\;dy=0.$$ (7)

Since this integral is 0 for any arbitrary age range and any time interval, we must conclude that the integrand itself is exactly 0:

$$\frac{\partial }{\partial t}n_{k}^{g}(t,y)+v\frac{\partial }{\partial y}n_{k}^{g}(t,y)=0.$$ (8)

We can always normalise the rate of ageing to a constant *v* = 1, meaning that this equation simplifies to the conservation law given by Eq (1) we started with above.

These equations are similar to a PDE derived by Burt Simon (see [72]), which was also used to study group selection. An important distinction is that our approach allows one to study group-selection using a system of *n* PDEs, rather than 1 PDE evolving on an *n*-dimensional surface. The system of PDEs can simplify the numerical problem of solving group-selection dynamics dramatically but is generally only tractable for biological systems in which groups can be assigned to categories in some simple way. Here, for example, we categorise groups by the genotype of their founding cell. If groups were formed from multiple founding cells, then the number of group types would increase dramatically, making the numerical solution of these equations much more difficult.

To incorporate assumptions about the birth and death of *k*-type groups in our PDE model, along with their production by other *l*-type groups due to mutation, we can simply extend Eq (1) to include source and sink terms. The production of *k*-type groups aged *y* due to the reproduction of all other *l*-type groups can be represented by a term

$$\int_{0}^{\infty }\sum_{l\in \{I\}}n_{l}^{g}(t,y^{\prime })b_{l}^{g}(t,y^{\prime })h_{lk}^{g}(y^{\prime },y)dy^{\prime }$$

where $b_{l}^{g}(t,y^{\prime })\in [0,\infty \}$ is the rate at which *l*-type groups aged *y*′ reproduce at time *t*, and $h_{lk}^{g}(y^{\prime },y)\in [0,1]$ is the conditional transition probability that a *k*-type group aged *y* is produced, given that an *l*-type group aged *y*′ just reproduced (in practice, we assume the age of a newly produced group is always *y* = 0). The death of groups can simply be modelled by a term

$$-n_{k}^{g}(t,y)d_{k}^{g}(t,y)$$

where $d_{k}^{g}(t,y)\in [0,\infty \}$ is simply the rate at which *k*-type group aged *y* die at time *t*. Incorporating these terms into Eq (1) gives

$$\frac{\partial n_{k}^{g}}{\partial t}(t,y)=\int_{0}^{\infty }\sum_{l\in \{I\}}n_{l}^{g}(t,y^{\prime })b_{l}^{g}(t,y^{\prime })h_{lk}^{g}(y^{\prime },y)dy^{\prime }-n_{k}^{g}(t,y)d_{k}^{g}(t,y)-\frac{\partial n_{k}^{g}}{\partial y}(t,y).$$ (9)

The extent to which there is between-group competition in our model therefore depends on variation between different types of groups in birth rates, $b_{k}^{g}(t,y)$, death rates, $d_{k}^{g}(t,y)$, and mutation transition probabilities, $h_{kl}^{g}(y,y\prime )$. These functions all depend on the within-group dynamics within each type of group.

### Within-group dynamics

Now let us describe what happens within groups as they age. We assume all groups are founded by a small number of cells (1 cell in most of our analyses) and undergo logistic growth as they age. The abundance of *i*-type cells within a *k*-type group aged *y* is denoted $n_{ki}^{c}(y)$. We assume that external forces have no impact on the change in abundance of cells of different types within a group. Thus, the rate of change in the abundance of *i*-type cells within a *k*-type group aged *y* depends only on age *y*, and not on external time *t*.

Changes in the abundance of different cell types within a particular *k*-type group are stochastic, but we assume that all groups of type *k* generally develop in the same way as they age, irrespective of the environment they were born into. In practice, we therefore characterise the expected development of a *k*-type group by calculating the average behaviour of 10,000 replicates of the stochastic dynamics of cells within the group. We use stochastic simulations rather than deterministic solutions to characterise the within-group dynamics because we are interested in the different rates at which mutant cell lineages invade within a *finite* subpopulation. If we used ordinary differential equations to characterise the same behaviour, cheater mutants would emerge and spread within each group deterministically very early on in the lifetime of groups due to the assumption of infinite population sizes. In contrast, our averaging approach shows that in finite systems, mutant cell lineages invade at vastly different rates in different types of groups. This is crucial for showing why pleiotropy is important in group selection. Groups founded by pleiotropic cooperators can outcompete groups founded by nonpleiotropic cooperators because pleiotropy slows the rate of invasion of mutant cell cheaters, making groups more competitive.

The stochastic dynamics within groups are characterised by a birth–death process with mutation. The birth rate of *i*-type cells in a *k*-type group aged *y* is given by $b_{kj}^{c}\in [0,\infty \}$. The death rate of *i*-type cells in a *k*-type group aged *y* is given by $d_{kj}^{c}\in [0,\infty \}$. Finally, the conditional transition probability that an *i*-type cell is produced, given that a *j*-type cell just reproduced (akin to a mutation transition probability), is denoted $h_{ji}^{c}\in [0,1]$. With these rates defined, we can simulate the stochastic evolutionary dynamics of any population. We generate sample paths using Gillespie’s Direct method [73].

### Solving the equations

So far we have described how selection and mutation acts on groups and cells within groups. How are these equations linked? The answer is that the group birth rate, $b_{l}^{g}(t,y^{\prime })$, death rate, $d_{l}^{g}(t,y^{\prime })$, and transition probability, $h_{lk}^{g}(y^{\prime },y),$ all depend on the outcome of the within-group cell dynamics. Thus, to solve the system of PDEs describing the dynamics of selection and mutation between groups, we first need to resolve the dynamics of selection and mutation between cells within each type of group.

We resolve the within-group dynamics of cells using custom-built stochastic simulations written in Matlab (R2019b). We then numerically solve the between-group dynamics given by Eq (9) using a custom-built finite-volume PDE solver written in Matlab (R2019b). We use a “superbee” flux limiter to control the rate of change of group relative density in regions where the group density function is not smooth [74].

### Modelling the evolution of pleiotropy and cooperation

Now that we have described a general model of multilevel selection in an age-structured population, we turn to describing how we use that model to study the evolution of cooperation and pleiotropy. Specifically, we focus on describing the birth, death, and mutation rates for cells and multicellular groups in our model. For reference, all of the parameters used in the model and their default values and ranges are shown in Table 1.

**Table 1:** List of parameters used in the model.

Parameter [= default value]	Description
*s*^*c*^ = 0.95	Strength of within-group selection.
*s*^*g*^ = 0.95	Strength of between-group selection.
*λ* = 20	Expected life span of groups (units of time). Note: The expected life span of a cell is roughly 1 unit of time.
*K* = 200	Carrying capacity of groups (size at maturity).
*μ* = 0.0001	Loss-of-function mutation rate of traits.
*ν* = 0.01	Relative rate of gain-of-function mutations to loss-of-function mutations.
*ϕ* = 0.0	Strength of pleiotropy.
*ζ* = 0.0	Cost of pleiotropy (% reduction of group function).
*α* = 0.0	Age of reproductive maturity.
*γ* = 0.0	Strength of germ line transmission.

### Cells

Cells in our model express 3 phenotypic traits of interest, which can either be in an active or inactive state. There are therefore 8 possible cell genotypes in our model (Fig 2B). We consider a cooperative trait $z_{u}^{c}\in \{0,1\}$, which is beneficial for the reproductive success of multicellular groups as a whole, but costly for the reproductive success of cells within the lifetime of a group, a private trait, $z_{v}^{c}\in \{0,1\}$, which is beneficial for the reproductive success of a cell expressing it within a group and beneficial for multicellular groups as a whole, and a “pleiotropy” trait, $z_{p}^{c}\in \{0,1\}$, which influences the types of cell mutations that can occur. Ordering cell traits as row vectors, [$z_{u}^{c},\;z_{v}^{c},\;z_{p}^{c}$], the genotype–phenotype map for cells can be represented by a matrix

$$Z^{c}=[\begin{array}{ccc} 0 & 0 & 0\\ 0 & 0 & 1\\ 0 & 1 & 0\\ 0 & 1 & 1\\ 1 & 0 & 0\\ 1 & 0 & 1\\ 1 & 1 & 0\\ 1 & 1 & 1 \end{array}].$$ (10)

Each of the 8 rows corresponds to a genotype, and each of the 3 columns corresponds to a trait, ordered public (cooperative), private, and pleiotropy. Cells with different phenotypes may vary in their ability to survive and reproduce within groups. The within-group birth and death rates of *i*-type cells in a *k*-type group aged *y* are given by

$$b_{ki}^{c}(y)=\frac{1-s^{c}z_{ui}^{c}}{1-s^{c}{\bar{z}}_{uk}^{c}(y)},$$ (11)

and

$$d_{ki}^{c}(y)=\frac{\left(1-s^{c}z_{vi}^{c}\right)}{\left(1-s^{c}{\bar{z}}_{vk}^{c}(y)\right)}\frac{\left(N_{k}^{c}(y)-1\right)}{K},$$ (12)

respectively, where *s*^*c*^ is the strength of selection on cell traits, ${\bar{z}}_{uk}^{c}(y)$ and ${\bar{z}}_{vk}^{c}(y)$ are the average expression of the cooperative and private traits in a *k*-type group aged *y*, respectively, and *N*_*k*_(*y*) is the size of a *k*-type group aged *y*. The component $N_{k}^{c}(y)-1$ in the death-rate prevents a group from dying because of stochastic extinction of all its cells, because its death rate is $d_{ki}^{c}(y)=0$ when the size of the group is $N_{k}^{c}(y)=1$.

Note that the expression of the cooperative trait $z_{ui}^{c}$ places a cost on *i*-type cell division relative to other cells in the group, the expression of the private trait, $z_{vi}^{c}$, gives *i*-type cells a survival advantage relative to other cells in the group, and $z_{pi}^{c}$ has no impact on cell birth rates or death rates at all. We are assuming here that the cooperative trait positively impacts the birth rate of cells and the loss of the private trait positively impacts the death rate of cells. These assumptions are based on the observations that extracellular growth factors and enzymes in cellular groups (cooperative traits) often promote growth [1], while cells lacking a functional metabolism (a private trait) often die [75].

We generally expect within-group selection to disfavour expression of the cooperative trait $z_{ui}^{c}$, favour the expression of the private noncooperative trait $z_{vi}^{c}$, and be neutral with respect to the pleiotropic trait $z_{pi}^{c}$. Similar to dos Santos and colleagues, we assume that selection is of equal strength with respect to the cooperative and private traits because we did not want to introduce biases by privileging 1 trait over another, but rather focus on the importance of pleiotropy [29]. In a variant of our model, we switch the cooperative trait to another private trait, $z_{wi}^{c}\in \{0,1\}$. To do this, we replace our usual birth rate function with

$$b_{ki}^{c}(y)=\frac{1-{s^{c}+s}^{c}z_{wi}^{c}}{1-s^{c}+s^{c}{\bar{z}}_{wk}^{c}(y)},$$ (13)

where the expression of the private trait $z_{wi}^{c}$ on *i*-type cell division is positive relative to nonexpressing cells in the group.

Cell mutations can occur during cell division. The conditional transition probability that an *i*-type ancestral cell produces a *j*-type descendant cell, given that it has reproduced is given by

$$h_{ij}^{c}=\phi p_{ij}^{c}+(1-\phi )q_{ij}^{c},$$ (14)

where *ϕ*∈[0, 1] is a parameter scaling the strength of pleiotropy, and $p_{ij}^{c}$ and $q_{ij}^{c}$ are elements of 2 different mutation matrices, the first of which represents a model of pleiotropic mutations, and the second of which represents a null model where pleiotropy has no effect. The parameter *ϕ* thus scales the likelihood that mutation rates are sampled from a mutation matrix in which pleioptropy has an effect versus a mutation matrix in which pleiotropy is absent. When *ϕ* is relatively small, mutations to traits are almost always independent events, and pleiotropy is therefore relatively weak or absent, but when *ϕ* is close to unity, mutations are often nonindependent events, where mutation of 1 trait influences the state of another trait.

The specifics of our asssumptions about the effects of pleiotropy are as follows. Under our null model, we have a mutation matrix ${Q^{c}=[q}_{ij}^{c}]$, where we simply assume that mutations affect each trait independently. We assume that loss-of-function mutations occur with rate *μ* and that gain-of-function mutations occur with rate *νμ*, where *ν*≤1. The mutation matrix for this model is then given by

$$Q^{c}=\left[\begin{array}{cccccccc} {\left(1-\nu \mu \right)}^{3} & {\left(1-\nu \mu \right)}^{2}\nu \mu & {\left(1-\nu \mu \right)}^{2}\nu \mu & (1-\nu \mu )\nu^{2}\mu^{2} & {\left(1-\nu \mu \right)}^{2}\nu \mu & (1-\nu \mu )\nu^{2}\mu^{2} & (1-\nu \mu )\nu^{2}\mu^{2} & \nu^{3}\mu^{3}\\ {\left(1-\nu \mu \right)}^{2}\mu & {\left(1-\nu \mu \right)}^{2}(1-\mu ) & (1-\nu \mu )\nu \mu^{2} & (1-\nu \mu )(1-\mu )\nu \mu & (1-\nu \mu )\nu \mu^{2} & (1-\nu \mu )(1-\mu )\nu \mu & \nu^{2}\mu^{3} & (1-\mu )\nu^{2}\mu^{2}\\ {\left(1-\nu \mu \right)}^{2}\mu & (1-\nu \mu )\nu \mu^{2} & {\left(1-\nu \mu \right)}^{2}(1-\mu ) & (1-\nu \mu )(1-\mu )\nu \mu & (1-\nu \mu )\nu \mu^{2} & \nu^{2}\mu^{3} & (1-\mu )(1-\nu \mu )\nu \mu & (1-\mu )\nu^{2}\mu^{2}\\ (1-\nu \mu )\mu^{2} & (1-\nu \mu )(1-\mu )\mu & (1-\nu \mu )(1-\mu )\mu & (1-\nu \mu ){\left(1-\mu \right)}^{2} & \nu \mu^{3} & (1-\mu )\nu \mu^{2} & (1-\mu )\nu \mu^{2} & {\left(1-\mu \right)}^{2}\nu \mu \\ {\left(1-\nu \mu \right)}^{2}\mu & (1-\nu \mu )\nu \mu^{2} & (1-\nu \mu )\nu \mu^{2} & \nu^{2}\mu^{3} & {\left(1-\nu \mu \right)}^{2}(1-\mu ) & (1-\nu \mu )(1-\mu )\nu \mu & (1-\nu \mu )(1-\mu )\nu \mu & (1-\mu )\nu^{2}\mu^{2}\\ (1-\nu \mu )\mu^{2} & (1-\nu \mu )(1-\mu )\mu & \nu \mu^{3} & (1-\mu )\nu \mu^{2} & (1-\nu \mu )(1-\mu )\mu & (1-\nu \mu ){\left(1-\mu \right)}^{2} & (1-\mu )\nu \mu^{2} & {\left(1-\mu \right)}^{2}\nu \mu \\ (1-\nu \mu )\mu^{2} & \nu \mu^{3} & (1-\nu \mu )(1-\mu )\mu & (1-\mu )\nu \mu^{2} & (1-\nu \mu )(1-\mu )\mu & (1-\mu )\nu \mu^{2} & (1-\nu \mu ){\left(1-\mu \right)}^{2} & {\left(1-\mu \right)}^{2}\nu \mu \\ \mu^{3} & (1-\mu )\mu^{2} & (1-\mu )\mu^{2} & {\left(1-\mu \right)}^{2}\mu & (1-\mu )\mu^{2} & {\left(1-\mu \right)}^{2}\mu & {\left(1-\mu \right)}^{2}\mu & {\left(1-\mu \right)}^{3} \end{array}\right]$$ (15)

Under our model of pleiotropy, we have a mutation matrix $P^{c}=[p_{ij}^{c}]$, where we assume that a loss-of-function mutation to either $z_{u}^{c}$ or $z_{v}^{c}$ drives a corresponding change in the other trait (i.e., that loss-of-function mutations have pleiotropic effects). However, we assume that pleiotropy is only active in the cell genotype in which all 3 traits are active (i.e., pleiotropic cooperators with phenotype $z_{u}^{c},z_{v}^{c},z_{p}^{c}=1$). The mutation matrix for this model is then given by

$$P^{c}=\left[\begin{array}{cccccccc} {\left(1-\nu \mu \right)}^{3} & {\left(1-\nu \mu \right)}^{2}\nu \mu & {\left(1-\nu \mu \right)}^{2}\nu \mu & (1-\nu \mu )\nu^{2}\mu^{2} & {\left(1-\nu \mu \right)}^{2}\nu \mu & (1-\nu \mu )\nu^{2}\mu^{2} & (1-\nu \mu )\nu^{2}\mu^{2} & \nu^{3}\mu^{3}\\ {\left(1-\nu \mu \right)}^{2}\mu & {\left(1-\nu \mu \right)}^{2}(1-\mu ) & (1-\nu \mu )\nu \mu^{2} & (1-\nu \mu )(1-\mu )\nu \mu & (1-\nu \mu )\nu \mu^{2} & (1-\nu \mu )(1-\mu )\nu \mu & \nu^{2}\mu^{3} & (1-\mu )\nu^{2}\mu^{2}\\ {\left(1-\nu \mu \right)}^{2}\mu & (1-\nu \mu )\nu \mu^{2} & {\left(1-\nu \mu \right)}^{2}(1-\mu ) & (1-\nu \mu )(1-\mu )\nu \mu & (1-\nu \mu )\nu \mu^{2} & \nu^{2}\mu^{3} & (1-\mu )(1-\nu \mu )\nu \mu & (1-\mu )\nu^{2}\mu^{2}\\ (1-\nu \mu )\mu^{2} & (1-\nu \mu )(1-\mu )\mu & (1-\nu \mu )(1-\mu )\mu & (1-\nu \mu ){\left(1-\mu \right)}^{2} & \nu \mu^{3} & (1-\mu )\nu \mu^{2} & (1-\mu )\nu \mu^{2} & {\left(1-\mu \right)}^{2}\nu \mu \\ {\left(1-\nu \mu \right)}^{2}\mu & (1-\nu \mu )\nu \mu^{2} & (1-\nu \mu )\nu \mu^{2} & \nu^{2}\mu^{3} & {\left(1-\nu \mu \right)}^{2}(1-\mu ) & (1-\nu \mu )(1-\mu )\nu \mu & (1-\nu \mu )(1-\mu )\nu \mu & (1-\mu )\nu^{2}\mu^{2}\\ (1-\nu \mu )\mu^{2} & (1-\nu \mu )(1-\mu )\mu & \nu \mu^{3} & (1-\mu )\nu \mu^{2} & (1-\nu \mu )(1-\mu )\mu & (1-\nu \mu ){\left(1-\mu \right)}^{2} & (1-\mu )\nu \mu^{2} & {\left(1-\mu \right)}^{2}\nu \mu \\ (1-\nu \mu )\mu^{2} & \nu \mu^{3} & (1-\nu \mu )(1-\mu )\mu & (1-\mu )\nu \mu^{2} & (1-\nu \mu )(1-\mu )\mu & (1-\mu )\nu \mu^{2} & (1-\nu \mu ){\left(1-\mu \right)}^{2} & {\left(1-\mu \right)}^{2}\nu \mu \\ 2\mu -\mu^{2} & 0 & 0 & 0 & 0 & 0 & {\left(1-\mu \right)}^{2}\mu & 1-(2\mu -\mu^{2})-{\left(1-\mu \right)}^{2}\mu \end{array}\right]$$ (16)

where the only difference between *P*^*c*^ and *Q*^*c*^ is in the final row.

### Multicellular groups

Groups in our model express their own phenotypic traits of interest that emerge from interactions of cells within a group. We usually assume that groups are founded by a single cell, in which case there are 8 group genotypes in our model. In a variant of our model, however, we assume that groups are founded by 2 cells. In this scenario, there are 8(8−1)/2+8 = 36 group genotypes. We assume that both the cooperative and private traits of cells are essential components of the ability of the groups to function as a cohesive whole in order to maximise survival and reproduction of the group. The functionality of *k*-type groups aged *y* is denoted $z_{fk}^{g}(y)\in [0,1]$ and defined as

$$z_{fk}^{g}(y)={\bar{z}}_{uk}^{c}(y){\bar{z}}_{vk}^{c}(y),$$ (17)

where there is a multiplicative effect of the average expression of the cooperative trait, ${\bar{z}}_{uk}^{c}(y)$, and average expression of the private trait, ${\bar{z}}_{vk}^{c}(y)$, among cells within the group at age *y*. It is important to note that whereas cell-level traits are all fixed within their lifetime, the functionality trait $z_{fk}^{g}(y)$ is expected to change throughout the lifetime of a group as it ages. We usually anticipate that within-group mutation–selection dynamics among cells will degrade group functionality as a function of age. In 1 variant of our model, we add in a cost of pleiotropy on the functioning of groups by defining

$$z_{fk}^{g}(y)={\bar{z}}_{uk}^{c}(y){\bar{z}}_{vk}^{c}(y)(1-\zeta {\bar{z}}_{pk}^{c}(y)),$$ (18)

where the component $1-\zeta {\bar{z}}_{pk}^{c}(y)$ allows us to explore what happens to the evolution of pleiotropy under the assumption that it carries a cost, *ζ*.

Whereas the expected growth rate of cells is implicitly defined as 1 cell division per unit time, we assume that groups have an expected life span *λ*>1, which gives an expected group reproduction rate of *ρ* = 1/*λ*. The larger *λ* is, the more cell divisions a group will experience within its life span. We also consider that group reproduction might depend on reproductive maturity, achieved at a fraction of the expected life span, *α*∈[0, 1]. For example, if *α* = 0.5 and *λ* = 50, then the age of group reproductive maturity will be 25 and groups will only be able to reproduce after that point. The reproductive maturity status of a *k*-type group aged *y* is denoted $z_{rk}^{g}(y)\in \{0,1\}$ and defined by

$$z_{rk}^{g}(y)=\left\{ \begin{array}{c} 0,y<\alpha \lambda \\ 1,y\geq \alpha \lambda . \end{array}\right.$$ (19)

The functionality trait and reproductive maturity trait influence the birth and death rates of groups within the group population. Specifically, the between-group birth and death rates of *k*-type groups aged *y* at time *t* are given by

$$b_{k}^{g}(t,y)=\frac{\rho (1-s^{g}+s^{g}z_{rk}^{g}(y))}{\left(1-s^{g}+s^{g}{\bar{z}}_{r}^{g}(t)\right)},$$ (20)

and

$$d_{k}^{g}(t,y)=\frac{\rho (1-s^{g}z_{fk}^{g}(y))}{\left(1-s^{g}{\bar{z}}_{f}^{g}(t)\right)},$$ (21)

respectively, where *s*^*g*^ is the strength of selection on group traits, ${\bar{z}}_{r}^{g}(t)$ is the fraction of multicellular groups in the group population that have reached reproductive maturity at time *t*, and ${\bar{z}}_{f}^{g}(t)$ is the average group functionality in the population at time *t*. Note that groups with a higher level of functionality gain a survival advantage over groups with a lower level of functionality. Thus, between-group selection favours maximal expression of both cooperative and private traits among cells.

An ancestral group will sometimes produce a descendant group with a different set of founding cell genotypes than it started life with. By default we assume that a descendant group is formed by choosing a founding cell at random from the ancestral group at the time at which it reproduced. Under these circumstances, the conditional transition probability that a *k*-type ancestral group produces an *l*-type descendant group, given that it has reproduced, is given by

$$h_{kl}^{g}(y,0)=\sum_{i\in I}x_{ki}^{c}(y)h_{il}^{c},$$ (22)

where $x_{kl}^{c}(y)=n_{kl}^{c}(y)/N_{k}^{c}(y)$ is the relative frequency of *l*-type cells in a *k*-type group aged *y* and $h_{kl}^{c}$ is the conditional transition probability that a *k*-type cell mutates to an *l*-type cell during the production of the founding cell of a new group. Of course, $h_{kk}^{g}(y,0)$ is nonzero and so the most likely scenario is that a cell does not mutate. Note that $n_{kl}^{c}(y)$ and $N_{k}^{c}(y)$ are the absolute abundance of *l*-type cells in a *k*-type group and the size of a *k*-type group aged *y*, respectively.

In our default scenario, the production of mutant descendant groups tends to become more likely as a group ages, because mutation–selection dynamics among cells degrade its clonality. In a later extension of the model, we consider the alternative possibility that groups might have a germ line. To model this scenario, we assume that with a probability *γ*, groups are founded by a cell with the same genotype as the ancestral group (except for rare mutations in germ line cells), and with probability 1−*γ*, they are founded by cells chosen at random with probability proportional to their frequency, as in our default scenario. The conditional transition probability that a *k*-type ancestral group produces an *l*-type descendant group, given that it has reproduced, in this scenario is given by

$$h_{kl}^{g}(y,0)=\gamma h_{kl}^{c}+(1-\gamma )\sum_{i\in I}x_{ki}^{c}(y)h_{il}^{c}.$$ (23)

## Supporting information

S1 Text Revisiting the analysis of dos Santos and colleagues [29]. This Supporting information goes through a previous study on the evolution of pleiotropy and concludes that a different modelling approach is needed.
(DOCX)

S1 FigWithin-group evolutionary dynamics of groups founded by cells with genotype *i* = 7.Within-group mutation selection dynamics are shown for a group founded by a cell with genotype *g*_7_, which actively expresses cooperation, $z_{u}^{c}=1$, a private trait, $z_{v}^{c}=1$, but no pleiotropy, $z_{p}^{c}=0$. Growth of the group as its age, *y*, increases, is logistic, with a carrying capacity *K* = 200. Dynamics are shown from left to right for 3 different strengths of pleiotropy, *ϕ*. The vertical dashed line in (A-C) represents the point at which mutant cell lineages make up 25% of the group. Note that the strength of pleiotropy has no effect on the within-group dynamics. (A) Changes in genotype abundances, *n*^*c*^(*y*). (B) Changes in genotype relative frequencies, *x*^*c*^(*y*). (C) Changes in the average levels of cooperation, private trait expression, and pleiotropy, ${\bar{z}}^{c}(y)$. (D) Differential fitness effects of loss-of-function mutations within the group. For example, the blue bar indicates the fitness effect of a loss-of-function mutation in the cooperative trait. Parameters: *s*^*c*^ = 0.95; *K* = 200; *μ* = 0.0001; *ν* = 0.01. The code required to generate this figure can be found at https://github.com/euler-mab/pleiotropy and https://zenodo.org/record/6367788#.YjSBVurP2Uk.(DOCX)

S2 FigWithin-group evolutionary dynamics of groups founded by cells with genotype *i* = 6.Within-group mutation selection dynamics are shown for a group founded by a cell with genotype *g*_6_, which actively expresses cooperation, $z_{u}^{c}=1$, pleiotropy, $z_{p}^{c}=1$, but no private trait, $z_{v}^{c}=0$. Growth of the group as its age, *y*, increases, is logistic, with a carrying capacity *K* = 200. Dynamics are shown from left to right for 3 different strengths of pleiotropy, *ϕ*. The vertical dashed line in (A-C) represents the point at which mutant cell lineages make up 25% of the group. Note that the strength of pleiotropy has no effect on the within-group dynamics. (A) Changes in genotype abundances, *n*^*c*^(*y*). (B) Changes in genotype relative frequencies, *x*^*c*^(*y*). (C) Changes in the average levels of cooperation, private trait expression, and pleiotropy, ${\bar{z}}^{c}(y)$. (D) Differential fitness effects of loss-of-function mutations within the group. Parameters: *s*^*c*^ = 0.95; *K* = 200; *μ* = 0.0001; *ν* = 0.01. The code required to generate this figure can be found at https://github.com/euler-mab/pleiotropy and https://zenodo.org/record/6367788#.YjSBVurP2Uk.(DOCX)

S3 FigWithin-group evolutionary dynamics of groups founded by cells with genotype *i* = 5.Within-group mutation selection dynamics are shown for a group founded by a cell with genotype *g*_5_, which actively expresses cooperation, $z_{u}^{c}=1$, but no private trait, $z_{v}^{c}=0$, and no pleiotropy, $z_{p}^{c}=0$. Growth of the group as its age, *y*, increases, is logistic, with a carrying capacity *K* = 200. Dynamics are shown from left to right for 3 different strengths of pleiotropy, *ϕ*. The vertical dashed line in (A-C) represents the point at which mutant cell lineages make up 25% of the group. Note that the strength of pleiotropy has no effect on the within-group dynamics. (A) Changes in genotype abundances, *n*^*c*^(*y*). (B) Changes in genotype relative frequencies, *x*^*c*^(*y*). (C) Changes in the average levels of cooperation, private trait expression, and pleiotropy, ${\bar{z}}^{c}(y)$. (D) Differential fitness effects of loss-of-function mutations within the group. Parameters: *s*^*c*^ = 0.95; *K* = 200; *μ* = 0.0001; *ν* = 0.01. The code required to generate this figure can be found at https://github.com/euler-mab/pleiotropy and https://zenodo.org/record/6367788#.YjSBVurP2Uk.(DOCX)

S4 FigWithin-group evolutionary dynamics of groups founded by cells with genotype *i* = 4.Within-group mutation selection dynamics are shown for a group founded by a cell with genotype *g*_4_, which actively expresses a private trait, $z_{v}^{c}=1$, and pleiotropy, $z_{p}^{c}=1$, but no cooperation, $z_{u}^{c}=0$. Growth of the group as its age, *y*, increases, is logistic, with a carrying capacity *K* = 200. Dynamics are shown from left to right for 3 different strengths of pleiotropy, *ϕ*. The vertical dashed line in (A-C) represents the point at which mutant cell lineages make up 25% of the group. Note that the strength of pleiotropy has no effect on the within-group dynamics. (A) Changes in genotype abundances, *n*^*c*^(*y*). (B) Changes in genotype relative frequencies, *x*^*c*^(*y*). (C) Changes in the average levels of cooperation, private trait expression, and pleiotropy, ${\bar{z}}^{c}(y)$. (D) Differential fitness effects of loss-of-function mutations within the group. Parameters: *s*^*c*^ = 0.95; *K* = 200; *μ* = 0.0001; *ν* = 0.01. The code required to generate this figure can be found at https://github.com/euler-mab/pleiotropy and https://zenodo.org/record/6367788#.YjSBVurP2Uk.(DOCX)

S5 FigWithin-group evolutionary dynamics of groups founded by cells with genotype *i* = 3.Within-group mutation selection dynamics are shown for a group founded by a cell with genotype *g*_4_, which actively expresses a private trait, $z_{v}^{c}=1$, but no cooperation, $z_{u}^{c}=0$, or pleiotropy, $z_{p}^{c}=0$. Growth of the group as its age, *y*, increases, is logistic, with a carrying capacity *K* =200. Dynamics are shown from left to right for 3 different strengths of pleiotropy, *ϕ*. The vertical dashed line in (A-C) represents the point at which mutant cell lineages make up 25% of the group. Note that the strength of pleiotropy has no effect on the within-group dynamics. (A) Changes in genotype abundances, *n*^*c*^(*y*). (B) Changes in genotype relative frequencies, *x*^*c*^(*y*). (C) Changes in the average levels of cooperation, private trait expression, and pleiotropy, ${\bar{z}}^{c}(y)$. (D) Differential fitness effects of loss-of-function mutations within the group. Parameters: *s*^*c*^ = 0.95; *K* = 200; *μ* = 0.0001; *ν* = 0.01. The code required to generate this figure can be found at https://github.com/euler-mab/pleiotropy and https://zenodo.org/record/6367788#.YjSBVurP2Uk.(DOCX)

S6 FigWithin-group evolutionary dynamics of groups founded by cells with genotype *i* = 2.Within-group mutation selection dynamics are shown for a group founded by a cell with genotype *g*_2_, which actively expresses pleiotropy, $z_{p}^{c}=1$, but no cooperation, $z_{u}^{c}=0$, a no private trait, $z_{v}^{c}=0$. Growth of the group as its age, *y*, increases, is logistic, with a carrying capacity *K* = 200. Dynamics are shown from left to right for 3 different strengths of pleiotropy, *ϕ*. The vertical dashed line in (A-C) represents the point at which mutant cell lineages make up 25% of the group. Note that the strength of pleiotropy has no effect on the within-group dynamics. (A) Changes in genotype abundances, *n*^*c*^(*y*). (B) Changes in genotype relative frequencies, *x*^*c*^(*y*). (C) Changes in the average levels of cooperation, private trait expression, and pleiotropy, ${\bar{z}}^{c}(y)$. (D) Differential fitness effects of loss-of-function mutations within the group. Parameters: *s*^*c*^ = 0.95; *K* = 200; *μ* = 0.0001; *ν* = 0.01. The code required to generate this figure can be found at https://github.com/euler-mab/pleiotropy and https://zenodo.org/record/6367788#.YjSBVurP2Uk.(DOCX)

S7 FigWithin-group evolutionary dynamics of groups founded by cells with genotype *i* = 1.Within-group mutation selection dynamics are shown for a group founded by a cell with genotype *g*_1_, which does not actively expresses any of cooperation, $z_{u}^{c}=0$, a private trait, $z_{v}^{c}=0$, or pleiotropy, $z_{p}^{c}=0$. Growth of the group as its age, *y*, increases, is logistic, with a carrying capacity *K* = 200. Dynamics are shown from left to right for 3 different strengths of pleiotropy, *ϕ*. The vertical dashed line in (A-C) represents the point at which mutant cell lineages make up 25% of the group. Note that the strength of pleiotropy has no effect on the within-group dynamics. (A) Changes in genotype abundances, *n*^*c*^(*y*). (B) Changes in genotype relative frequencies, *x*^*c*^(*y*). (C) Changes in the average levels of cooperation, private trait expression, and pleiotropy, ${\bar{z}}^{c}(y)$. The differential fitness effects of loss-of-function mutations are not shown, because *g*_1_ cells have no traits to lose. Parameters: *s*^*c*^ = 0.95; *K* = 200; *μ* = 0.0001; *ν* = 0.01. The code required to generate this figure can be found at https://github.com/euler-mab/pleiotropy and https://zenodo.org/record/6367788#.YjSBVurP2Uk.(DOCX)

S8 FigLong-term evolutionary dynamics to illustrate a case where the evolution of cooperation depends entirely on pleiotropy.Dynamics are shown for the global population of cells over time, *t*, which encompasses many generations of cell groups. These dynamics, therefore, encompass both within-group and between-group selection dynamics. We show 3 strengths of pleiotropy, *ϕ*, to capture 3 qualitatively different scenarios. Stronger pleiotropy is associated with more rapid and complete evolution of both pleiotropy and cooperation. (A) Changes in global genotype relative frequencies, *x*^*c*^(*t*). (B) Changes in the global average levels of cooperation, private trait expression, and pleiotropy, ${\bar{z}}^{c}(t)$. In the absence of pleiotropy *ϕ* = 0, cooperation fails to evolve. (C) Average change in traits over a group lifetime, measured as the difference between the average trait values among groups aged *y* to those expected from their founding cell at birth. Parameters: *s*^*c*^ = *s*^*g*^ = 0.95; *K* = 500; *μ* = 0.0001; *ν* = 0.01; *λ* = 25. The code required to generate this figure can be found at https://github.com/euler-mab/pleiotropy and https://zenodo.org/record/6367788#.YjSBVurP2Uk.(DOCX)

S9 Fig Private trait evolution does not favour pleiotropy and vice versa. We explored a model in which the cooperative trait was replaced by another private trait. Heatmaps show average trait values of 2 private traits and pleiotropy among the global population of cells (across all groups) at steady state in our revised model. Results are shown for 3 group sizes (increasing from top to bottom). Both private traits evolve to fixation under all parameter values, but pleiotropy is never favoured. The dotted line marks the boundary between pleiotropy having no effect (control case) and pleiotropy having an effect on the outcome of mutations. Parameters: *s*^*c*^ = 0.95; *K* = 200; *μ* = 0.0001; *ν* = 0.01. The code required to generate this figure can be found at https://github.com/euler-mab/pleiotropy and https://zenodo.org/record/6367788#.YjSBVurP2Uk.
(DOCX)

S10 FigWithin-group evolutionary dynamics of groups founded by 8 cells with all 8 genotypes represented.Within-group mutation selection dynamics are shown for a group founded by cells with all possible genotypes and phenotypes represented. Growth of the group as its age, *y*, increases, is logistic, with a carrying capacity *K* = 200. Dynamics are shown from left to right for 3 different strengths of pleiotropy, *ϕ*. Note that the strength of pleiotropy has no effect on the within-group dynamics. (A) Changes in genotype abundances, *n*^*c*^(*y*). (B) Changes in genotype relative frequencies, *x*^*c*^(*y*). (C) Changes in the average levels of cooperation, private trait expression, and pleiotropy, ${\bar{z}}^{c}(y)$. (D) Differential fitness effects of loss-of-function mutations within the group. Parameters: *s*^*c*^ = 0.95; *K* = 200; *μ* = 0.0001; *ν* = 0.01. The code required to generate this figure can be found at https://github.com/euler-mab/pleiotropy and https://zenodo.org/record/6367788#.YjSBVurP2Uk.(DOCX)

S11 Fig Pleiotropy stabilises cooperation when groups are founded by 2 cells rather than one. We explored a model in which 2 cells are selected uniformly at random from the ancestor group to found each descendant group (rather than a single cell, which we assume in the main paper). This assumption lowers the expected relatedness in groups at the point at which they form. Heatmaps show average trait values among the global population of cells (across all groups) at steady state in our model. Results are shown for 3 group sizes (increasing from top to bottom). As when groups are founded by a single cell, pleiotropy is favoured when the strength of pleiotropy, *ϕ*, is higher. The overall levels of cooperation are lower when groups are founded by 2 cells rather than one, but the evolution of pleiotropy still promotes the evolution of cooperation. The dotted line marks the boundary between pleiotropy having no effect (control case) and pleiotropy having an effect on the outcome of mutations. Parameters: *s*^*c*^ = *s*^*g*^ = 0.95; *K* = 200; *μ* = 0.0001; *ν* = 0.01. The code required to generate this figure can be found at https://github.com/euler-mab/pleiotropy and https://zenodo.org/record/6367788#.YjSBVurP2Uk.
(DOCX)

S12 FigPleiotropy evolves to stabilise cooperation even when costly.We explored a model in which pleiotropy carries a cost on multicellular function. We assumed multicellular group function is reduced by a factor $1-\zeta {\bar{z}}_{p}^{c}$, where *ζ* is the cost of pleiotropy, and ${\bar{z}}_{p}^{c}$ is the average pleiotropy in a group. Heatmaps show average trait values among the global population of cells (across all groups) at steady state in our model. Results are shown for 3 levels of cost (increasing from top to bottom). Pleiotropy evolves even when it carries a 1%, 2%, or 5% cost to group functionality. When costly, it is especially favoured when groups are longer lived and therefore require mechanisms to limit the spread of noncooperative mutant lineages. The dotted line marks the boundary between pleiotropy having no effect (control case) and pleiotropy having an effect on the outcome of mutations. Parameters: *s*^*c*^ = *s*^*g*^ = 0.95; *K* = 200; *μ* = 0.0001; *ν* = 0.01; *K* = 200. The code required to generate this figure can be found at https://github.com/euler-mab/pleiotropy and https://zenodo.org/record/6367788#.YjSBVurP2Uk.(DOCX)

S13 Fig Pleiotropy evolves to stabilise cooperation even when group selection is weak. We explored a model in which the strength of group selection, *s*^*g*^, is varied. Heatmaps show average trait values among the global population of cells (across all groups) at steady state in our model. Results are shown for 3 strengths of group selection (increasing from top to bottom). Stronger group selection favours both cooperation and pleiotropy, but strong pleiotropy can help cooperation evolve even when group selection is weak, (*s*^*g*^ = 0.05). The dotted line marks the boundary between pleiotropy having no effect (control case) and pleiotropy having an effect on the outcome of mutations. Parameters: *s*^*c*^ = 0.95; *K* = 200; *μ* = 0.0001; *ν* = 0.01; *K* = 200. The code required to generate this figure can be found at https://github.com/euler-mab/pleiotropy and https://zenodo.org/record/6367788#.YjSBVurP2Uk.
(DOCX)

S14 Fig The rate of gain-of-function mutations has little impact on the dynamics of pleiotropy evolution. We varied the relative rate of gain-of-function mutations to loss-of-function mutations, *ν*. Heatmaps show average trait values among the global population of cells (across all groups) at steady state in our model. Results are shown for 3 gain-of-function rates (increasing from top to bottom). The gain-of-function ratio had a marginal effect on the evolution of pleiotropy, favouring slightly higher rates when *ν* is higher. The dotted line marks the boundary between pleiotropy having no effect (control case) and pleiotropy having an effect on the outcome of mutations. Parameters: *s*^*c*^ = *s*^*g*^ = 0.95; *K* = 200; *μ* = 0.0001; *K* = 200. The code required to generate this figure can be found at https://github.com/euler-mab/pleiotropy and https://zenodo.org/record/6367788#.YjSBVurP2Uk.
(DOCX)

S15 Fig Strong pleiotropy can help rescue cooperation even in the face of high mutation rates. We varied the loss-of-function mutation rate, *μ*. Heatmaps show average trait values among the global population of cells (across all groups) at steady state in our model. Results are shown for 3 loss-of-function rates (increasing from top to bottom). Higher mutation rates disfavoured the evolution of cooperation, but pleiotropy still evolved at higher strengths of pleiotropy and for lower group sizes. The evolution of pleiotropy was associated with a stabilisation of cooperation even at higher mutation rates. The dotted line marks the boundary between pleiotropy having no effect (control case) and pleiotropy having an effect on the outcome of mutations. Parameters: *s*^*c*^ = *s*^*g*^ = 0.95; *K* = 200; *ν* = 0.01. The code required to generate this figure can be found at https://github.com/euler-mab/pleiotropy and https://zenodo.org/record/6367788#.YjSBVurP2Uk.
(DOCX)

S16 Fig Lower mutation rates increases cooperation but pleiotropy still evolves. We varied the loss-of-function mutation rate, *μ*, using values 10-fold lower than the typical value used in the main text. Heatmaps show average trait values among the global population of cells (across all groups) at steady state in our model. Results are shown for 3 loss-of-function rates (increasing from top to bottom). With 10-fold lower mutation rates, we found that cooperation breaks down only for longer group life spans, *λ*. When cooperation is threatened by breakdown, however, we still found that pleiotropy evolves to rescue it. The dotted line marks the boundary between pleiotropy having no effect (control case) and pleiotropy having an effect on the outcome of mutations. Parameters: *s*^*c*^ = *s*^*g*^ = 0.95; *K* = 200; *ν* = 0.01; *c* = 0. The code required to generate this figure can be found at https://github.com/euler-mab/pleiotropy and https://zenodo.org/record/6367788#.YjSBVurP2Uk.
(DOCX)

S17 Fig Lower mutation rates increase cooperation but pleiotropy still evolves, even when pleiotropy is costly. We varied the gain-of-function mutation rate, *μ*, using values 10-fold lower than the typical value used in the main text, and assumed that the evolution of pleiotropy decreases group function by 2%. Heatmaps show average trait values among the global population of cells (across all groups) at steady state in our model. Results are shown for 3 loss-of-function rates (increasing from top to bottom). With 10-fold lower mutation rates and a cost, we found that pleiotropy evolves most frequently when the mutation rate is higher and cooperation is under the greatest threat. The dotted line marks the boundary between pleiotropy having no effect (control case) and pleiotropy having an effect on the outcome of mutations. Parameters: *s*^*c*^ = *s*^*g*^ = 0.95; *K* = 200; *ν* = 0.01; *ζ* = 0.02. The code used to produce this figure may be found at https://github.com/euler-mab/pleiotropy.
(DOCX)

S18 Fig Reproduction at maturity hinders cooperation and has mixed effects on the evolution of pleiotropy. We varied the age fraction of the expected life span at which groups reach reproductive maturity, *α*, and assumed that the evolution of pleiotropy decreases group function by 2%. Heatmaps show average trait values among the global population of cells (across all groups) at steady state in our model. Results are shown for 3 reproductive maturity parameters (increasing the age at which maturity is reached from top to bottom). Increasing the age of reproductive maturity has a marginal positive effect on the evolution of pleiotropy for shorter life spans, but a marginal negative effect on cooperation. The dotted line marks the boundary between pleiotropy having no effect (control case) and pleiotropy having an effect on the outcome of mutations. Parameters: *s*^*c*^ = *s*^*g*^ = 0.95; *K* = 200; *ν* = 0.01; *ζ* = 0.02. The code required to generate this figure can be found at https://github.com/euler-mab/pleiotropy and https://zenodo.org/record/6367788#.YjSBVurP2Uk.
(DOCX)

S19 Fig When mutation-driven breakdown of cooperation is a problem, pleiotropy stabilises cooperation. We modified the individual-based model of dos Santos and colleagues to compare what happens when pleiotropy cannot evolve (left) as compared to when it can evolve (right). We varied the length of the within-group growth phase *k* during which spontaneous mutants can arise and invade within groups, and the relatedness *r* at the point at which groups form. We follow the evolution of a private trait, cooperative trait, and pleiotropy trait. (A) Evolutionary dynamics of all 3 traits for a within-group growth phase of *k* = 30 and *r* = 1. (B) Steady-state levels of all 3 traits under a when the length of the within-group growth phase is varied (x-axis) for *r* = 1. (C) Steady-state levels of all 3 traits when relatedness at the point at which groups form is varied for a within-group growth phase of *k* = 30. Other parameters: *b* = 0.11, *c* = 0.1, *g* = 0.5, mutation rate *μ* = 0.001, number of groups *n*_*g*_ = 1000. All plots are averages of 10 replicates. The code required to generate this figure can be found at https://github.com/euler-mab/pleiotropy and https://zenodo.org/record/6367788#.YjSBVurP2Uk.
(DOCX)

S20 Fig Pleiotropy evolves to stabilise cooperation even when the loss-of-function mutation rate is 10× greater than the gain-of-function rate. We performed simulations using the mutation model used by dos Santos and colleagues in the production of their S5 Fig. This again reveals conditions where pleiotropy evolves and increases cooperation as it does. We varied the length of the within-group growth phase *k* during which spontaneous mutants can arise and invade within groups, and the relatedness *r* at the point at which groups form. We follow the evolution of a private trait, cooperative trait, and pleiotropy trait. (A) Evolutionary dynamics of all 3 traits for a within-group growth phase of *k* = 30 and *r* = 1. (B) Steady-state levels of all 3 traits under a when the length of the within-group growth phase is varied (x-axis) for *r* = 1. (C) Steady-state levels of all 3 traits when relatedness at the point at which groups form is varied for a within-group growth phase of *k* = 30. Other parameters: *b* = 0.11, *c* = 0.1, *g* = 0.5, mutation rate *μ* = 0.001, number of groups *n*_*g*_ = 1000. All plots are averages of 10 replicates. The code required to generate this figure can be found at https://github.com/euler-mab/pleiotropy and https://zenodo.org/record/6367788#.YjSBVurP2Uk.
(DOCX)

S21 Fig Pleiotropy evolves to stabilise cooperation even when the loss-of-function mutation rate is 100× greater than the gain-of-function rate. We performed simulations using the mutation model used by dos Santos and colleagues in the production of their S6 Fig. This again reveals conditions where pleiotropy evolves and increases cooperation as it does. We varied the length of the within-group growth phase *k* during which spontaneous mutants can arise and invade within groups, and the relatedness *r* at the point at which groups form. We follow the evolution of a private trait, cooperative trait, and pleiotropy trait. (A) Evolutionary dynamics of all 3 traits for a within-group growth phase of *k* = 30 and *r* = 1. (B) Steady-state levels of all 3 traits under a when the length of the within-group growth phase is varied (x-axis) for *r* = 1. (C) Steady-state levels of all 3 traits when relatedness at the point at which groups form is varied for a within-group growth phase of *k* = 30. Other parameters: *b* = 0.11, *c* = 0.1, *g* = 0.5, mutation rate *μ* = 0.001, number of groups *n*_*g*_ = 1000. All plots are averages of 10 replicates. The code required to generate this figure can be found at https://github.com/euler-mab/pleiotropy and https://zenodo.org/record/6367788#.YjSBVurP2Uk.
(DOCX)
