# Supplementary material for: Pleiotropic constraints promote the evolution of cooperation in cellular groups
Source: PLoS Biol. 2022 Jun 3;20(6):e3001626. doi: 10.1371/journal.pbio.3001626 (PMC9166655; doi:10.1371/journal.pbio.3001626)
Supplement: S1 Text — This Supporting information goes through a previous study on the evolution of pleiotropy and concludes that a different modelling approach is needed. (DOCX) [file pbio.3001626.s001.docx]

## S1 Text – revisiting the analysis of Santos et al (2018).

**Summary**

A recent theory paper by dos Santos et al (2018) sought to challenge the hypothesis that pleiotropy can help to stabilise cooperation [1]. Specifically, the authors claimed that pleiotropy does not evolve to stabilise cooperation except under unrealistic conditions, such as when genetic architecture cannot evolve. In this supplement, we study the two modelling approaches used – analytical and individual-based modelling – and show that both fail to capture a key piece of biology that drives pleiotropy evolution: the emergence of cheater mutants within groups that breakdown cooperation. The analytical approach does not allow for this process at all, which means that the benefits of pleiotropy were missed. The agent-based model does, in principle, allow for such within-group evolution but is only studied for parameters where within-group evolution has little impact on cooperation. As result, the authors find that pleiotropy can slow down the rate at which cheaters emerge within cooperative groups, but claim that pleiotropy does not help to stabilise cooperation on evolutionary timescales. By studying the case when within-group evolution threatens cooperation, we show that pleiotropy both rapidly evolves and increases the level of cooperation as it evolves. However, assumptions built into the dos Santos, et al model, including unbounded exponential growth within groups, means it is challenging to explore this effect further. For this reason, we developed the alternative model presented in our main text.

**Background to the dos Santos et al model**

The key to understanding cooperative evolution lies in the identification of mechanisms that limit the potential benefits of non-cooperative strategies, including so-called cheater mutants [2,3]. As discussed in the main text, it has been suggested that one such mechanism is pleiotropy, in which two or more phenotypes are affected by a single genetic locus [4–9]. Specifically, when a cooperative trait is pleiotropically linked to a private (personally beneficial) trait, a mutation that ablates cooperation now brings with it personal costs via the concurrent loss of the private trait. This genotype to phenotype map will limit the possible mutations that produce viable non-cooperative strategies and, it has been argued, increase the level of cooperation in a given system.

However, on some time scale, mutations are expected that would circumvent the pleiotropy [1,10], which means that it is important to also understand how pleiotropy itself is maintained over evolutionary time. One possibility is that the pleiotropy evolves neutrally or is under positive selection for a reason independent of cooperation [11], such as to ensure the correct regulation of a number of traits, and this may help to limit the emergence of cheater mutants in a multicellular group as a by-product [6,10]. A second possibility is that the pleiotropy arose as a result of cooperation being enforced in a focal individual by another individual, where natural selection favours enforcing cooperation in a way that is costly to escape [12]. For example, hosts mechanisms that select against non-cooperative symbionts can generate a pleiotropic link between symbiont cooperation and survival in the host [13]. Finally, there is the possibility that the collective benefits of cooperation drive the evolution of pleiotropy to ensure that cheater mutants do not arise and spread. This last explanation is expected to be most important in cellular groups, which can reach large enough sizes for the de-novo emergence of cheaters to be a problem [12,14].

These hypotheses have been long discussed, particularly in reference to microbial cooperation and multicellularity, based on the discovery of pleiotropic constraints in a number of empirical studies [4,6,8,9] and an agent-based model [7]. However, until the dos Santos et al study, the evolution of pleiotropy had not been studied using the tools of social evolution theory. Their paper served an important function, therefore, in subjecting the pleiotropy hypothesis to detailed study, and indeed challenging the idea that pleiotropy is important in cooperative evolution [1]. The authors showed that pleiotropy only evolves in conditions in which kin selection is also at play and, from this, it was concluded pleiotropy does not contribute to the evolution of cooperation. Instead, it was argued that it is only kin selection that really matters for cooperation, not pleiotropy. As we will discuss, we agree that the evolution of pleiotropy in their model (and indeed our model in the main text) rests upon kin selection. However, we do not find that one can infer from this that pleiotropy “*Pleiotropy does not help stabilise cooperation over evolutionary time*…[1]”. As we show here and in the main text, in the presence of kin selection, pleiotropy can be critical for stabilising the evolution of cooperation.

The reason that dos Santos et al reached such a different conclusion to us is due to their modelling choices. In particular, their approaches suffer from a number of issues that prevented them from capturing the effects of evolution within cellular groups. Fixing these issues reveals how pleiotropy can promote cooperation. Specifically, here we show that: i) allowing within-group evolution in the analytical model of dos Santos et al leads to the prediction that pleiotropic cooperation can be favoured over non-pleiotropic cooperation; and ii) biologically-motivated changes to the parameters in their individual-based model shows that pleiotropy evolves and, importantly, increases cooperation as it does so.

## ****Analytical model****

Dos Santos et al begin with an analytical model of a population of haploid individuals subdivided into a large number of groups, each of size $N$. They assume that there is a single generation of within-group growth in which individuals reproduce and die before their offspring disperse to compete globally for the opportunity to found a new group. The assumption of a single generation of within-group growth is made to keep things simple. However, this assumption is an issue because it misses a key piece of the biology of cellular systems. As dos Santos et al discuss in their opening, non-cooperative mutants can arise as cellular groups grow that threaten cooperation, and pleiotropy has the potential to reduce the viability of these mutants within groups. However, if there is no within-group evolution, this process is excluded and the associated evolutionary benefit of pleiotropy is missed.

Turning to the specifics, in their baseline model the authors assume there is no pleiotropy with other traits. Individuals produce a public good that provides a benefit $b/N$to everyone in the same group (including the focal) but comes at a personal cost $c$. From this, fecundity functions for a focal defector $f_{D}$ and a focal cooperator $f_{C}$ are given as

|  | $f_{D}=1+x_{D}\left( N-1 \right)\frac{b}{N},$ | (1) |
| --- | --- | --- |

and,

|  | $f_{C}=1-c+\frac{b}{N}+x_{C}\left( N-1 \right)\frac{b}{N},$ | (2) |
| --- | --- | --- |

where $x_{D}$ and $x_{C}$ are the expected frequencies of cooperators among the social partners of a focal defector and cooperator, respectively. The difference between these two variables is denoted by $r$ and is consistent with the definition of relatedness from kin selection theory, calculated as the regression coefficient from the regression of social partner traits on focal individual traits across the population [15]. Under these assumptions, the condition under which cooperation is favoured can be written as a form of Hamilton’s rule as

|  | $\underset{r}{\underbrace{\left( x_{C}-x_{D} \right)}}\underset{\tilde{b}}{\underbrace{\left( N-1 \right)\frac{b}{N}}}+\underset{-\tilde{c}}{\underbrace{\left( \frac{b}{N}-c \right)}}>0,$ | (3) |
| --- | --- | --- |

where ${r=x}_{C}-x_{D}$ is relatedness, $\tilde{b}=\left( N-1 \right)b/N$ is the net benefit of cooperation, and $-\tilde{c}=b/N-c$ is its net cost. However, the prediction of Hamilton’s rule here relies crucially on there being no within-group growth period in which cooperation can break down. The predicted scope for cooperation, therefore, can be thought of as an upper bound one can expect given the type of population structure in the model and the mechanisms of dispersal. When the evolution of non-cooperators within groups is allowed, this process is expected to reduce cooperation below this upper bound.

Next, dos Santos et al introduce pleiotropy. Specifically, they consider a scenario in which cooperation is linked pleiotropically to a private-good trait that is important for survival and reproduction. As a result of not having the private trait (being a ‘private-good nonproducer’) individuals suffer a fecundity cost, $d$. With this, the authors then implicitly define new fecundity functions for private-good nonproducers and pleiotropic cooperators, as

|  | $f_{PN}=f_{D}-d,$ | (4) |
| --- | --- | --- |

and,

|  | $f_{PC}=f_{C}$ | (5) |
| --- | --- | --- |

respectively. These definitions are critical as they implicitly assume here that pleiotropy has no impact on the social environment. That is, equations (4) and (5) rest on the assumption that the expected frequencies of cooperators among the social partners of a focal pleiotropic cooperator and a non-pleiotropic cooperator are the same, i.e. $x_{PC}=x_{C}$.

This assumption only holds true if there is no within-group growth period or zero relatedness, neither of which are realistic assumptions for most cellular groups (main text). With positive relatedness and several rounds of within-group growth, there is expected to be differences in the frequencies of cooperators among the social partners of a focal pleiotropic cooperator and a non-pleiotropic cooperator. Specifically, we expect pleiotropic cooperators will give rise to private-good nonproducers, which have low fitness ($d>0$) and will invade the group poorly. By contrast, non-pleiotropic cooperators will give rise to defectors which are expected to compete well in groups and increase in frequency much more than private-good nonproducers. The result is that pleiotropic cooperators will have a more favourable social environment than non-pleiotropic cooperators ($x_{PC}>x_{C}$) and therefore generally have a higher fecundity than them ($f_{PC}>f_{C}$). This simple argument predicts that pleiotropy can be favoured whenever there are costs associated with losing a private trait. However, to see this mechanism of pleiotropy in action, one needs a model that explicitly captures between-group variation in the rate of mutation-driven breakdown of cooperation within groups. The second model in the dos Santos et al paper goes some way to capturing the process, but again fails to do so, for reasons we discuss next.

## ****Individual-based model****

Dos Santos et al use an individual-based (or agent-based) model that is a variant of the classic haystack model of Maynard-Smith [16]. As in their analytical model, there are two lifecycle stages in this model, a within-group growth phase, and a between-group dispersal phase. During the within-group growth phase, individuals compete locally within groups in a public goods game. Selection tends to favour genotypes that do not cooperate, because these genotypes reap the benefits of cooperation without contributing to its costs. During the dispersal phase, individuals from all groups enter a mixed global pool and a small subset of founders is selected uniformly at random to found a new set of groups. Here, selection tends to favour cooperation, because groups with more cooperators contribute more individuals to the global pool.

Their individual-based model is significantly better than their analytical model at capturing the biology of cellular groups because, in principle, it permits the mutation-driven breakdown of cooperation within groups. It also enables the potential for between-group variation in mechanisms that repress competition within groups (i.e. pleiotropy), provided that group selection is strong enough. However, dos Santos et al do not focus on the potential importance of these dynamics, but rather what happens when relatedness is varied at the point at which groups form. With this analysis, they make the point that – without relatedness – pleiotropy will not evolve in their model. As discussed above, we agree with this point. However, relatedness is often positive in cellular groups (above, main text). In these scenarios, dos Santos et al find that pleiotropy *can* evolve, but still conclude that it is unimportant for cooperation on evolutionary timescales. Why do they again reach a different conclusion to us? Here, we revisit their code and identify areas where dos Santos et al make assumptions which fail to capture underlying biology relevant for the evolution of pleiotropy. When we adjust these assumptions, we find pleiotropy is better able to increase the evolved levels of cooperation.

First, dos Santos et al assume that the growth phase period is short in length. This means that mutation-driven breakdown of cooperation has limited impact. That is, the evolved level of cooperation in their models almost always matches the upper bound provided by Hamilton’s rule (see their Fig 2A). As discussed, this assumption contrasts with the empirical observations that the emergence of cheaters can be a problem, both for microbial groups and multicellular organisms so long as the growth phase is long enough. For example, in experiments with the bacterium *P. aeruginosa*, spontaneous cheater mutants were not a significant problems between 0-15 days of growth, but had invaded to a frequency of ~25% in colonies after 20 days of growth, and >40% in colonies after 25 days of growth [6]. Similarly, cancer is seen in many multicellular organisms and rises with the number of cell divisions in different tissues [17]. To study how their model behaves when mutation within groups does cause problems for cooperation, we explored a range of longer growth phase parameters $k\in\{10, 15, 20,25,30\}$ in contrast to their single growth phase parameter $k=10$.

Second, dos Santos et al model a small number of groups. This means that there is significant stochasticity in the model. To decrease such stochasticity, we changed the default number of groups from $n_{g}=100$ to $n_{g}=1000$. dos Santos et al also assume that pleiotropic cooperators are already present in the population at time $t=0$. As a more conservative assumption, we exclude pleiotropic cooperators from the initial state to ask whether pleiotropy can evolve via mutation from non-pleiotropic genotypes. We find that pleiotropy evolves under these conditions and increases the level of cooperation as it does so. In S19A Fig (below) we show the evolutionary dynamics of the private trait, cooperative trait, and pleiotropy, in a scenario in which pleiotropy cannot evolve (left) versus one in which it can evolve (right).

Whereas dos Santos et al used a within-group growth period of $k=10$, and found that pleiotropy typically does not improve the evolved levels of cooperation (see their Fig 2), we find a stronger impact of pleiotropy on cooperation for extended growth periods (S19B Fig). Note that, natural selection for cooperation is modest in these simulations and only evolves for very high relatedness (S19C Fig). However, once there is some level of cooperation that can be threatened by within-group processes, we find that pleiotropy will evolve to help stabilise it. This finding is robust to changes in the assumptions about the relative rate of loss-of-function and gain-of-function mutations (see S20 and S21 Fig). Whenever groups persist long enough for mutation to threaten cooperation, the potential benefits of pleiotropy are clear. It is important to note that in this scenario, when pleiotropy does arise, it is working alongside kin selection to promote cooperation. Hence, pleiotropy is not an alternative to kin selection but can work synergistically alongside it to greatly enhance cooperation.

We find, therefore, that there can be stronger effects of pleiotropy on cooperation in the model of dos Santos et al if one makes reasonable changes in assumptions. This finding on its own does not show that pleiotropy is expected to be important across a wider range of parameters than those we have shown here. However, there are structural issues with the dos Santos et al model that prevent us from going further. For example, we found that the assumption of unbounded exponential growth within groups meant that performing simulations with higher benefits, $b$, and higher values of the growth-phase parameter $k$ were computationally intractable. More generally, although the haystack model has often been used to highlight the tension between within group and between group selection, it is our contention that the variant developed by dos Santos et al does not capture cellular systems well. Assumptions of unrestricted exponential growth, non-overlapping cellular generations, high mutation rates are all potentially problematic. In the main text, therefore, we develop a more explicit model of the evolution of cellular groups that allows us to freely change these assumptions. This model suggests that many cellular groups, including multicellular organisms, have been shaped by the evolution of pleiotropic constraints that limit the breakdown of cooperation.

# References for S1 Text

1. dos Santos M, Ghoul M, West SA. Pleiotropy, cooperation, and the social evolution of genetic architecture. PLoS Biol. 2018;16: 1–25. doi:10.1371/journal.pbio.2006671

2. West SA, Griffin AS, Gardner A. Evolutionary Explanations for Cooperation. Current Biology. 2007. pp. 661–672. doi:10.1016/j.cub.2007.06.004

3. Ghoul M, Griffin AS, West SA. Toward an evolutionary definition of cheating. Evolution. 2014.

4. Foster KR, Shaulsky G, Strassmann JE, Queller DC, Thompson CRL. Pleiotropy as a mechanism to stabilize cooperation. Nature. 2004;431: 693–6. doi:10.1038/nature02894

5. Evan G, Lowe S, Cepero E. Intrinsic tumour suppression. Nature. 2004;432: 1–9.

6. Dandekar AA, Chugani S, Greenberg EP. Bacterial quorum sensing and metabolic incentives to cooperate. Science (80- ). 2012;338: 264–266. doi:10.1126/science.1227289

7. Frénoy A, Taddei F, Misevic D. Genetic architecture promotes the evolution and maintenance of cooperation. PLoS Comput Biol. 2013;9: e1003339.

8. Sathe S, Mathew A, Agnoli K, Eberl L, Kümmerli R. Genetic architecture constrains exploitation of siderophore cooperation in the bacterium Burkholderia cenocepacia. Evol Lett. 2019;3: 610–622.

9. Wang M, Schaefer AL, Dandekar AA, Greenberg EP. Quorum sensing and policing of Pseudomonas aeruginosa social cheaters. Proc Natl Acad Sci U S A. 2015;112: 2187–2191. doi:10.1073/pnas.1500704112

10. Mitri S, Foster KR. Pleiotropy and the low cost of individual traits promote cooperation. Evolution (N Y). 2016;70: 488–494. doi:10.1111/evo.12851

11. Wagner GP, Zhang J. The pleiotropic structure of the genotype-phenotype map: The evolvability of complex organisms. Nat Rev Genet. 2011;12: 204–213. doi:10.1038/nrg2949

12. Ågren JA, Davies NG, Foster KR. Enforcement is central to the evolution of cooperation. Nat Ecol Evol. 2019;3. doi:10.1038/s41559-019-0907-1

13. Foster KR, Parkinson K, Thompson CRL. What can microbial genetics teach sociobiology? Trends Genet. 2007;23: 74–80. doi:10.1016/j.tig.2006.12.003

14. Foster KR. The sociobiology of molecular systems. Nat Rev Genet. 2011;12: 193–203. doi:10.1038/nrg2903

15. Bijma P. Multilevel selection 4: Modeling the relationship of indirect genetic effects and group size. Genetics. 2010;186: 1029–1031. doi:10.1534/genetics.110.120485

16. Maynard-Smith J. Group selection and kin selection. Nature. 1964;201: 1145–1147.

17. Tomasetti C, Vogelstein B. Variation in cancer risk among tissues can be explained by the number of stem cell divisions. Science (80- ). 2015;347: 78–81. doi:10.1126/science.1260825
