# Supplementary material for: Pleiotropic constraints promote the evolution of cooperation in cellular groups
Source: PLoS Biol. 2022 Jun 3;20(6):e3001626. doi: 10.1371/journal.pbio.3001626 (PMC9166655; doi:10.1371/journal.pbio.3001626)
Supplement: S1 Fig — Within-group mutation selection dynamics are shown for a group founded by a cell with genotype g7, which actively expresses cooperation, zuc=1, a private trait, zvc=1, but no pleiotropy, zpc=0. Growth of the group as its age, y, increases, is logistic, with a carrying capacity K = 200. Dynamics are shown from left to right for 3 different strengths of pleiotropy, ϕ. The vertical dashed line in (A-C) represents the point at which mutant cell lineages make up 25% of the group. Note that the strength of pleiotropy has no effect on the within-group dynamics. (A) Changes in genotype abundances, nc(y). (B) Changes in genotype relative frequencies, xc(y). (C) Changes in the average levels of cooperation, private trait expression, and pleiotropy, z¯c(y). (D) Differential fitness effects of loss-of-function mutations within the group. For example, the blue bar indicates the fitness effect of a loss-of-function mutation in the cooperative trait. Parameters: sc = 0.95; K = 200; μ = 0.0001; ν = 0.01. The code required to generate this figure can be found at https://github.com/euler-mab/pleiotropy and https://zenodo.org/record/6367788#.YjSBVurP2Uk. (DOCX) [file pbio.3001626.s002.docx]

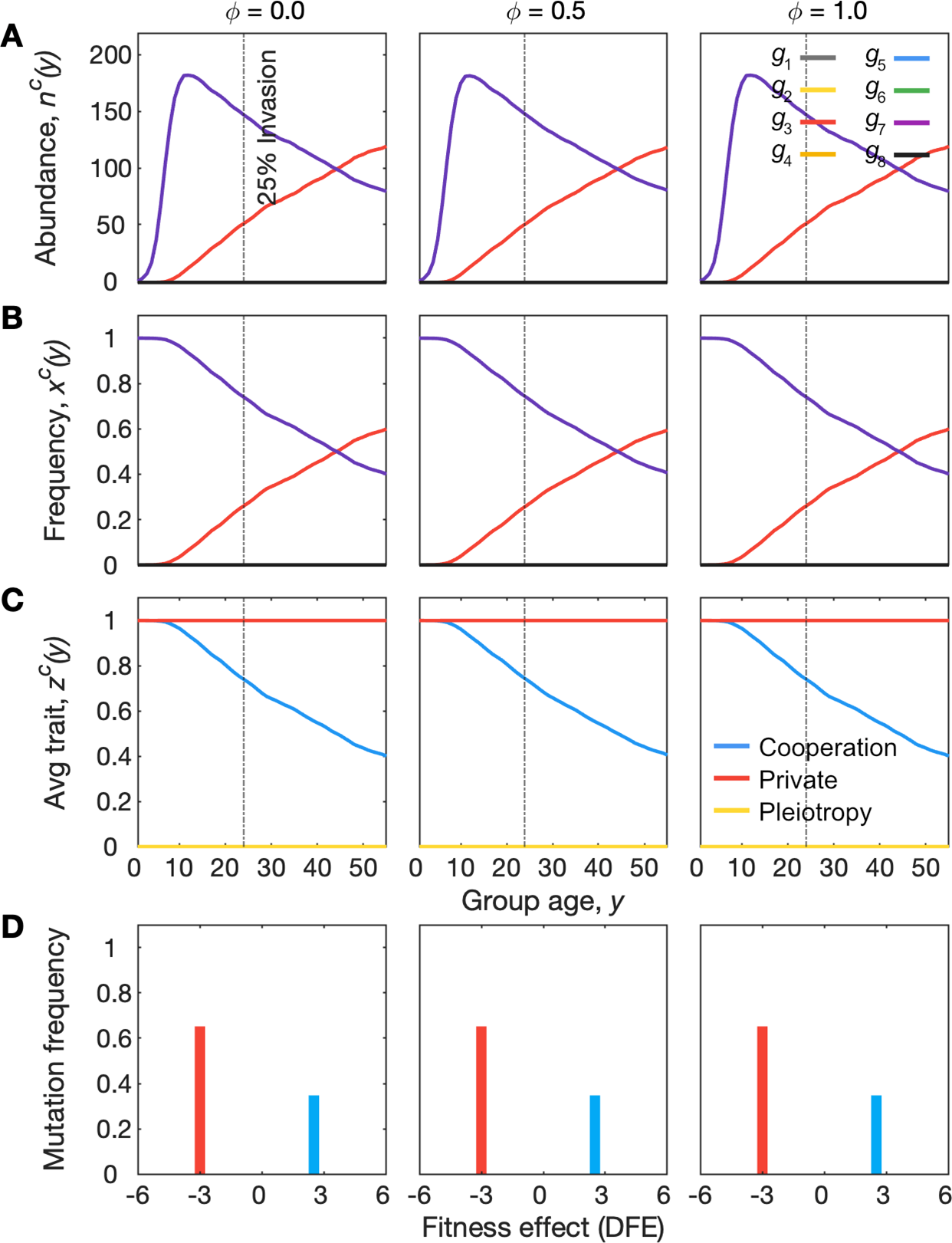


**S1 Fig. Within-group evolutionary dynamics of groups founded by cells with genotype** $\boldsymbol{i=}$**7.** Within-group mutation selection dynamics are shown for a group founded by a cell with genotype $g_{7}$, which actively expresses cooperation, $z_{u}^{c}=1$, a private trait, $z_{v}^{c}=1$, but no pleiotropy, $z_{p}^{c}=0$. Growth of the group as its age, $y$, increases, is logistic, with a carrying capacity $K=200$. Dynamics are shown from left to right for three different strengths of pleiotropy, $\phi$. The vertical dashed line in (A-C) represents the point at which mutant cell lineages make up 25% of the group. Note that the strength of pleiotropy has no effect on the within-group dynamics. (A) Changes in genotype abundances, $n^{c}(y)$. (B) Changes in genotype relative frequencies, $x^{c}(y)$. (C) Changes in the average levels of cooperation, private trait expression, and pleiotropy, $\bar{z}^{c}(y)$. (D) Differential fitness effects of loss-of-function mutations within the group. For example, the blue bar indicates the fitness effect of a loss-of-function mutation in the cooperative trait. Parameters: $s^{c}=0.95$; $K=200$; $\mu=0.0001$; $\nu=0.01$. The code required to generate this Figure can be found at https://github.com/euler-mab/pleiotropy and https://zenodo.org/record/6367788#.YjSBVurP2Uk.
