# Supplementary material for: Pleiotropic constraints promote the evolution of cooperation in cellular groups
Source: PLoS Biol. 2022 Jun 3;20(6):e3001626. doi: 10.1371/journal.pbio.3001626 (PMC9166655; doi:10.1371/journal.pbio.3001626)
Supplement: S8 Fig — Dynamics are shown for the global population of cells over time, t, which encompasses many generations of cell groups. These dynamics, therefore, encompass both within-group and between-group selection dynamics. We show 3 strengths of pleiotropy, ϕ, to capture 3 qualitatively different scenarios. Stronger pleiotropy is associated with more rapid and complete evolution of both pleiotropy and cooperation. (A) Changes in global genotype relative frequencies, xc(t). (B) Changes in the global average levels of cooperation, private trait expression, and pleiotropy, z¯c(t). In the absence of pleiotropy ϕ = 0, cooperation fails to evolve. (C) Average change in traits over a group lifetime, measured as the difference between the average trait values among groups aged y to those expected from their founding cell at birth. Parameters: sc = sg = 0.95; K = 500; μ = 0.0001; ν = 0.01; λ = 25. The code required to generate this figure can be found at https://github.com/euler-mab/pleiotropy and https://zenodo.org/record/6367788#.YjSBVurP2Uk. (DOCX) [file pbio.3001626.s009.docx]

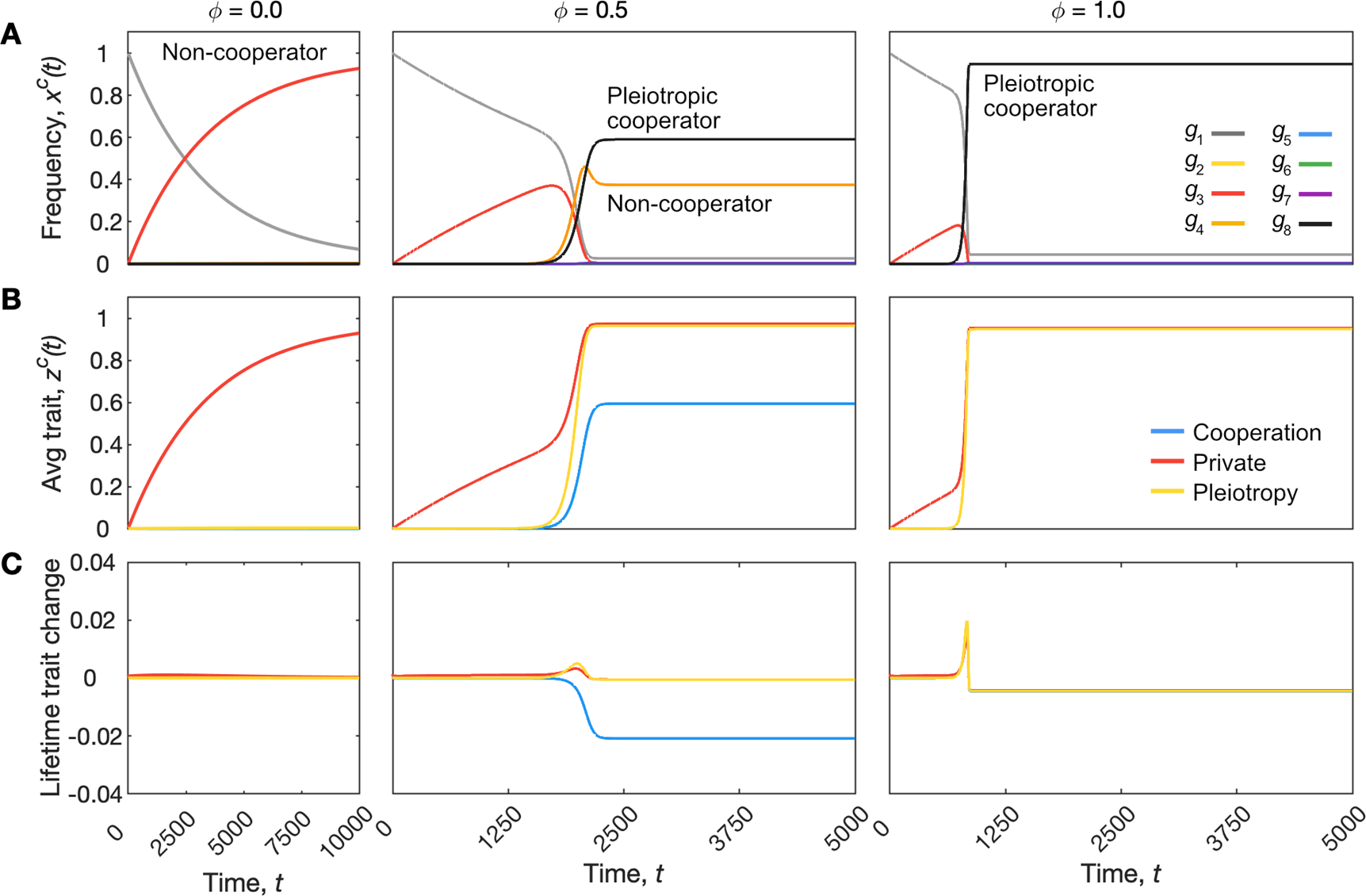


**S8 Fig. Long-term evolutionary dynamics to illustrate a case where the evolution of cooperation depends entirely on pleiotropy.** Dynamics are shown for the global population of cells over time, $t$, which encompasses many generations of cell groups. These dynamics, therefore, encompass both within-group and between-group selection dynamics. We show three strengths of pleiotropy, $\phi$, to capture three qualitatively-different scenarios. Stronger pleiotropy is associated with more rapid and complete evolution of both pleiotropy and cooperation. (A) Changes in global genotype relative frequencies, $x^{c}(t)$. (B) Changes in the global average levels of cooperation, private trait expression, and pleiotropy, $\bar{z}^{c}(t)$. In the absence of pleiotropy $\phi$= 0, cooperation fails to evolve. (C) Average change in traits over a group lifetime, measured as the difference between the average trait values among groups aged $y$ to those expected from their founding cell at birth. Parameters: $s^{c}=s^{g}=0.95$; $K=500$; $\mu=0.0001$; $\nu=0.01$; $\lambda=25$. The code required to generate this Figure can be found at https://github.com/euler-mab/pleiotropy and https://zenodo.org/record/6367788#.YjSBVurP2Uk.
