# Supplementary material for: Pleiotropic constraints promote the evolution of cooperation in cellular groups
Source: PLoS Biol. 2022 Jun 3;20(6):e3001626. doi: 10.1371/journal.pbio.3001626 (PMC9166655; doi:10.1371/journal.pbio.3001626)
Supplement: S9 Fig — We explored a model in which the cooperative trait was replaced by another private trait. Heatmaps show average trait values of 2 private traits and pleiotropy among the global population of cells (across all groups) at steady state in our revised model. Results are shown for 3 group sizes (increasing from top to bottom). Both private traits evolve to fixation under all parameter values, but pleiotropy is never favoured. The dotted line marks the boundary between pleiotropy having no effect (control case) and pleiotropy having an effect on the outcome of mutations. Parameters: sc = 0.95; K = 200; μ = 0.0001; ν = 0.01. The code required to generate this figure can be found at https://github.com/euler-mab/pleiotropy and https://zenodo.org/record/6367788#.YjSBVurP2Uk. (DOCX) [file pbio.3001626.s010.docx]

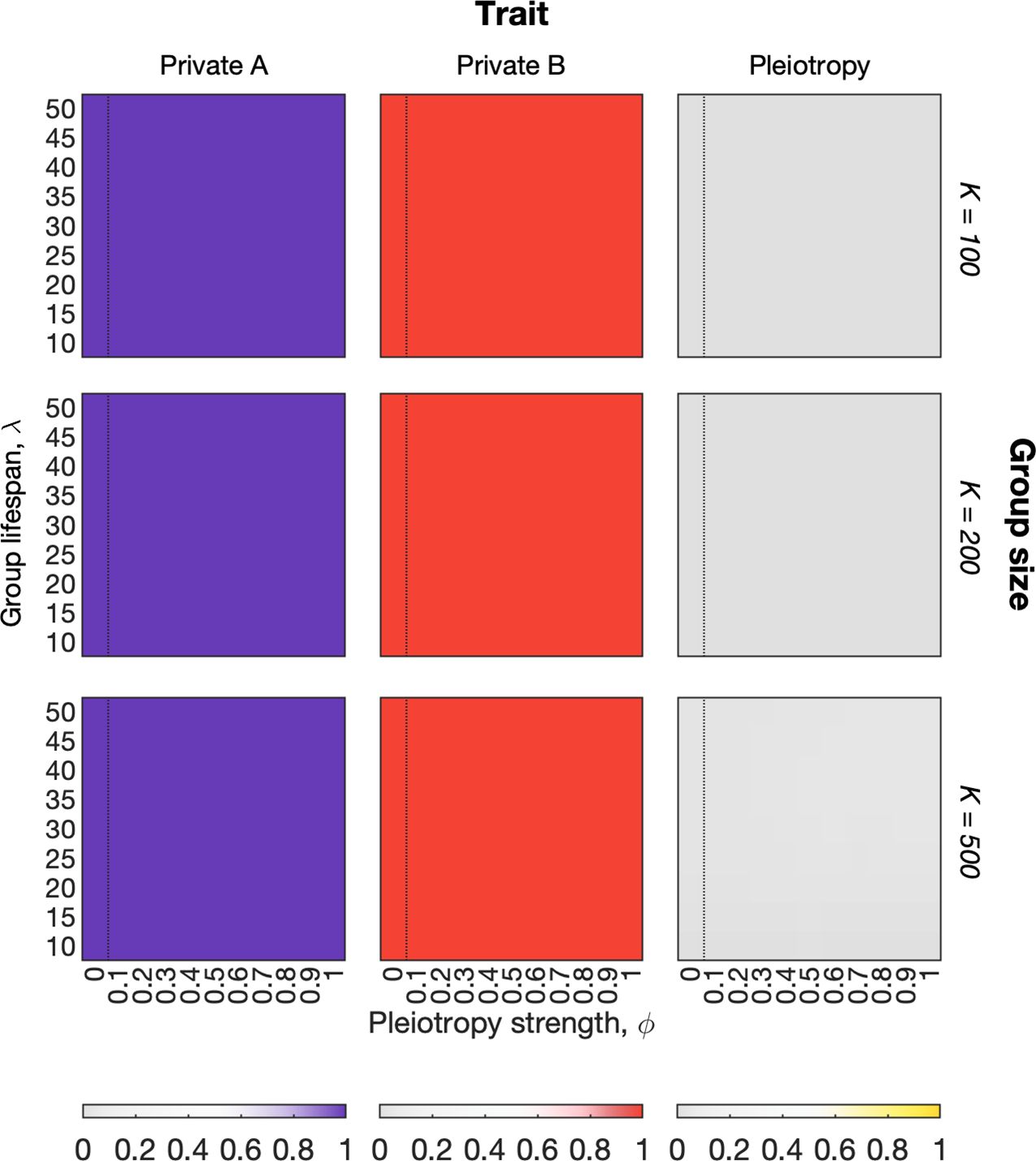


**S9 Fig. Private trait evolution does not favour pleiotropy, and vice versa.** We explored a model in which the cooperative trait was replaced by another private trait. Heatmaps show average trait values of two private traits and pleiotropy among the global population of cells (across all groups) at steady state in our revised model. Results are shown for three group sizes (increasing from top to bottom). Both private traits evolve to fixation under all parameter values, but pleiotropy is never favoured. The dotted line marks the boundary between pleiotropy having no effect (control case) and pleiotropy having an effect on the outcome of mutations. Parameters: $s^{c}=0.95$; $K=200$; $\mu=0.0001$; $\nu=0.01$. The code required to generate this Figure can be found at https://github.com/euler-mab/pleiotropy and https://zenodo.org/record/6367788#.YjSBVurP2Uk..
