# Supplementary material for: Pleiotropic constraints promote the evolution of cooperation in cellular groups
Source: PLoS Biol. 2022 Jun 3;20(6):e3001626. doi: 10.1371/journal.pbio.3001626 (PMC9166655; doi:10.1371/journal.pbio.3001626)
Supplement: S11 Fig — We explored a model in which 2 cells are selected uniformly at random from the ancestor group to found each descendant group (rather than a single cell, which we assume in the main paper). This assumption lowers the expected relatedness in groups at the point at which they form. Heatmaps show average trait values among the global population of cells (across all groups) at steady state in our model. Results are shown for 3 group sizes (increasing from top to bottom). As when groups are founded by a single cell, pleiotropy is favoured when the strength of pleiotropy, ϕ, is higher. The overall levels of cooperation are lower when groups are founded by 2 cells rather than one, but the evolution of pleiotropy still promotes the evolution of cooperation. The dotted line marks the boundary between pleiotropy having no effect (control case) and pleiotropy having an effect on the outcome of mutations. Parameters: sc = sg = 0.95; K = 200; μ = 0.0001; ν = 0.01. The code required to generate this figure can be found at https://github.com/euler-mab/pleiotropy and https://zenodo.org/record/6367788#.YjSBVurP2Uk. (DOCX) [file pbio.3001626.s012.docx]

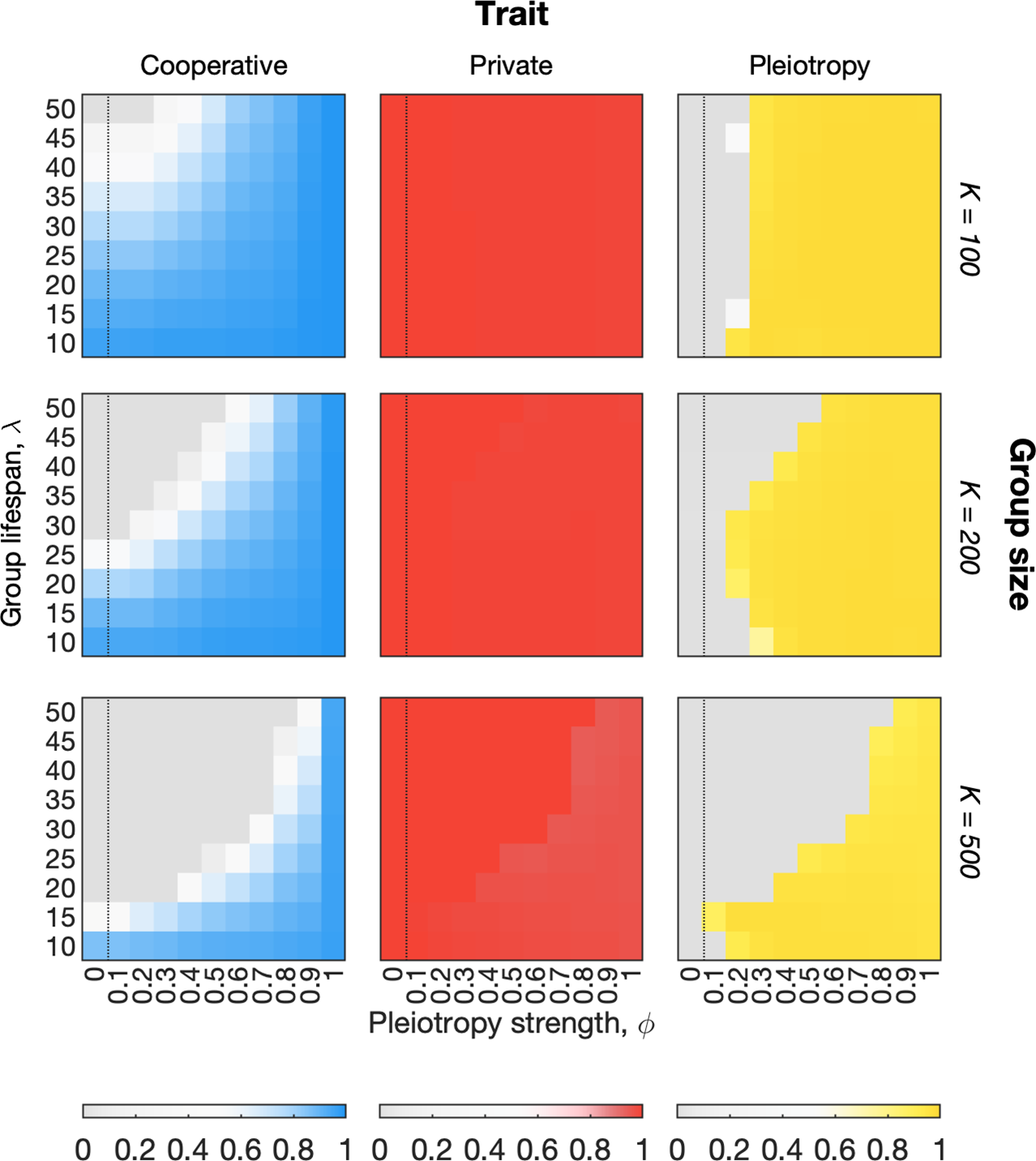


**S11 Fig. Pleiotropy stabilises cooperation when groups are founded by two cells rather than one.** We explored a model in which two cells are selected uniformly at random from the ancestor group to found each descendant group (rather than a single cell, which we assume in the main paper). This assumption lowers the expected relatedness in groups at the point at which they form. Heatmaps show average trait values among the global population of cells (across all groups) at steady state in our model. Results are shown for three group sizes (increasing from top to bottom). As when groups are founded by a single cell, pleiotropy is favoured when the strength of pleiotropy, $\phi$, is higher. The overall levels of cooperation are lower when groups are founded by two cells rather than one, but the evolution of pleiotropy still promotes the evolution of cooperation. The dotted line marks the boundary between pleiotropy having no effect (control case) and pleiotropy having an effect on the outcome of mutations. Parameters: $s^{c}=s^{g}=0.95$; $K=200$; $\mu=0.0001$; $\nu=0.01$. The code required to generate this Figure can be found at https://github.com/euler-mab/pleiotropy and https://zenodo.org/record/6367788#.YjSBVurP2Uk.
