# Supplementary material for: Pleiotropic constraints promote the evolution of cooperation in cellular groups
Source: PLoS Biol. 2022 Jun 3;20(6):e3001626. doi: 10.1371/journal.pbio.3001626 (PMC9166655; doi:10.1371/journal.pbio.3001626)
Supplement: S12 Fig — We explored a model in which pleiotropy carries a cost on multicellular function. We assumed multicellular group function is reduced by a factor 1−ζz¯pc, where ζ is the cost of pleiotropy, and z¯pc is the average pleiotropy in a group. Heatmaps show average trait values among the global population of cells (across all groups) at steady state in our model. Results are shown for 3 levels of cost (increasing from top to bottom). Pleiotropy evolves even when it carries a 1%, 2%, or 5% cost to group functionality. When costly, it is especially favoured when groups are longer lived and therefore require mechanisms to limit the spread of noncooperative mutant lineages. The dotted line marks the boundary between pleiotropy having no effect (control case) and pleiotropy having an effect on the outcome of mutations. Parameters: sc = sg = 0.95; K = 200; μ = 0.0001; ν = 0.01; K = 200. The code required to generate this figure can be found at https://github.com/euler-mab/pleiotropy and https://zenodo.org/record/6367788#.YjSBVurP2Uk. (DOCX) [file pbio.3001626.s013.docx]

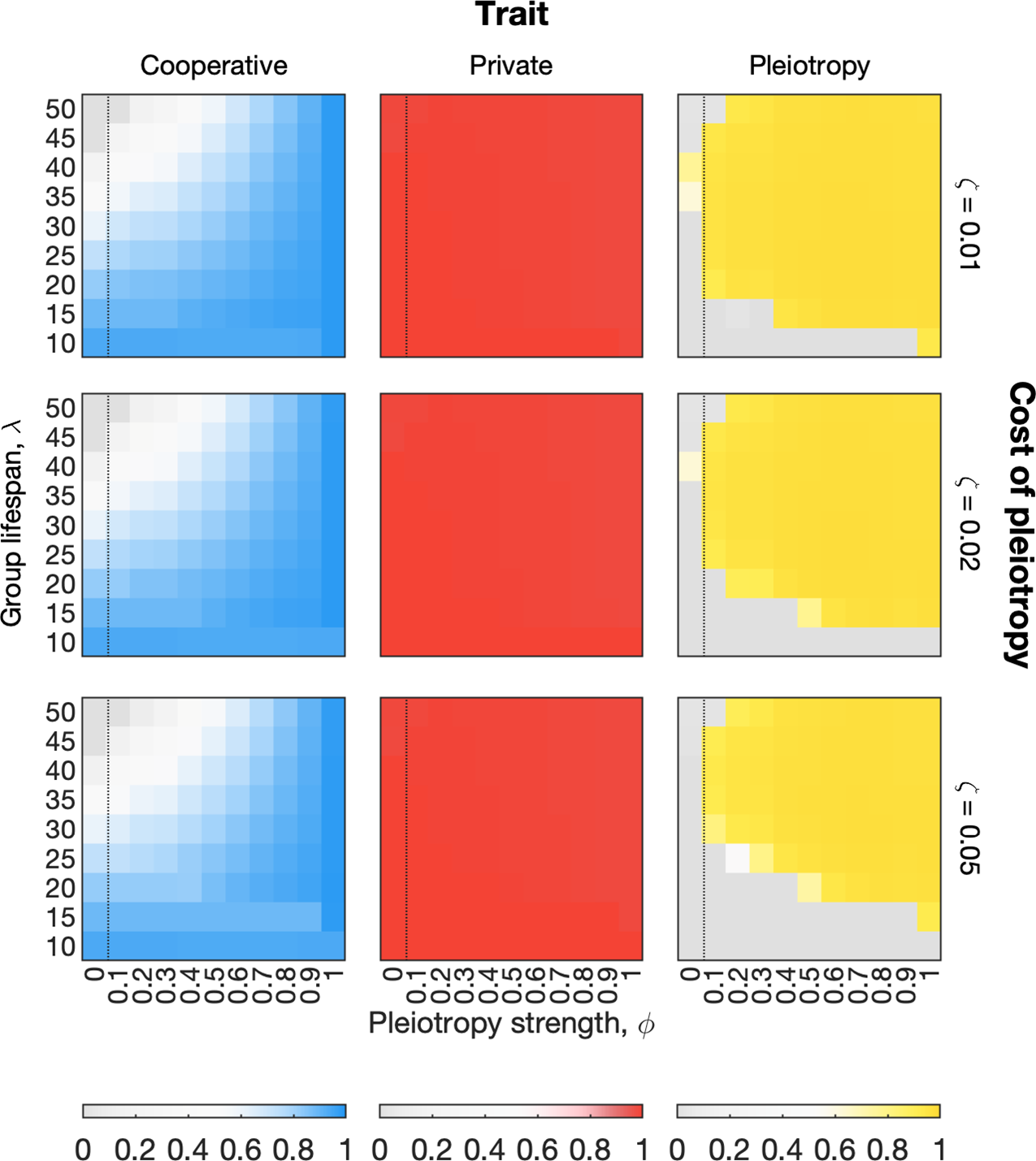


**S12 Fig. Pleiotropy evolves to stabilise cooperation even when costly.** We explored a model in which pleiotropy carries a cost on multicellular function. We assumed multicellular group function is reduced by a factor $1-\zeta\bar{z}_{p}^{c}$, where $\zeta$ is the cost of pleiotropy, and $\bar{z}_{p}^{c}$ is the average pleiotropy in a group. Heatmaps show average trait values among the global population of cells (across all groups) at steady state in our model. Results are shown for three levels of cost (increasing from top to bottom). Pleiotropy evolves even when it carries a 1%, 2% or 5% cost to group functionality. When costly, it is especially favoured when groups are longer-lived and therefore require mechanisms to limit the spread of non-cooperative mutant lineages. The dotted line marks the boundary between pleiotropy having no effect (control case) and pleiotropy having an effect on the outcome of mutations. Parameters: $s^{c}=s^{g}=0.95$; $K=200$; $\mu=0.0001$; $\nu=0.01; K=200$. The code required to generate this Figure can be found at https://github.com/euler-mab/pleiotropy and https://zenodo.org/record/6367788#.YjSBVurP2Uk.
