# Supplementary material for: Pleiotropic constraints promote the evolution of cooperation in cellular groups
Source: PLoS Biol. 2022 Jun 3;20(6):e3001626. doi: 10.1371/journal.pbio.3001626 (PMC9166655; doi:10.1371/journal.pbio.3001626)
Supplement: S13 Fig — We explored a model in which the strength of group selection, sg, is varied. Heatmaps show average trait values among the global population of cells (across all groups) at steady state in our model. Results are shown for 3 strengths of group selection (increasing from top to bottom). Stronger group selection favours both cooperation and pleiotropy, but strong pleiotropy can help cooperation evolve even when group selection is weak, (sg = 0.05). The dotted line marks the boundary between pleiotropy having no effect (control case) and pleiotropy having an effect on the outcome of mutations. Parameters: sc = 0.95; K = 200; μ = 0.0001; ν = 0.01; K = 200. The code required to generate this figure can be found at https://github.com/euler-mab/pleiotropy and https://zenodo.org/record/6367788#.YjSBVurP2Uk. (DOCX) [file pbio.3001626.s014.docx]

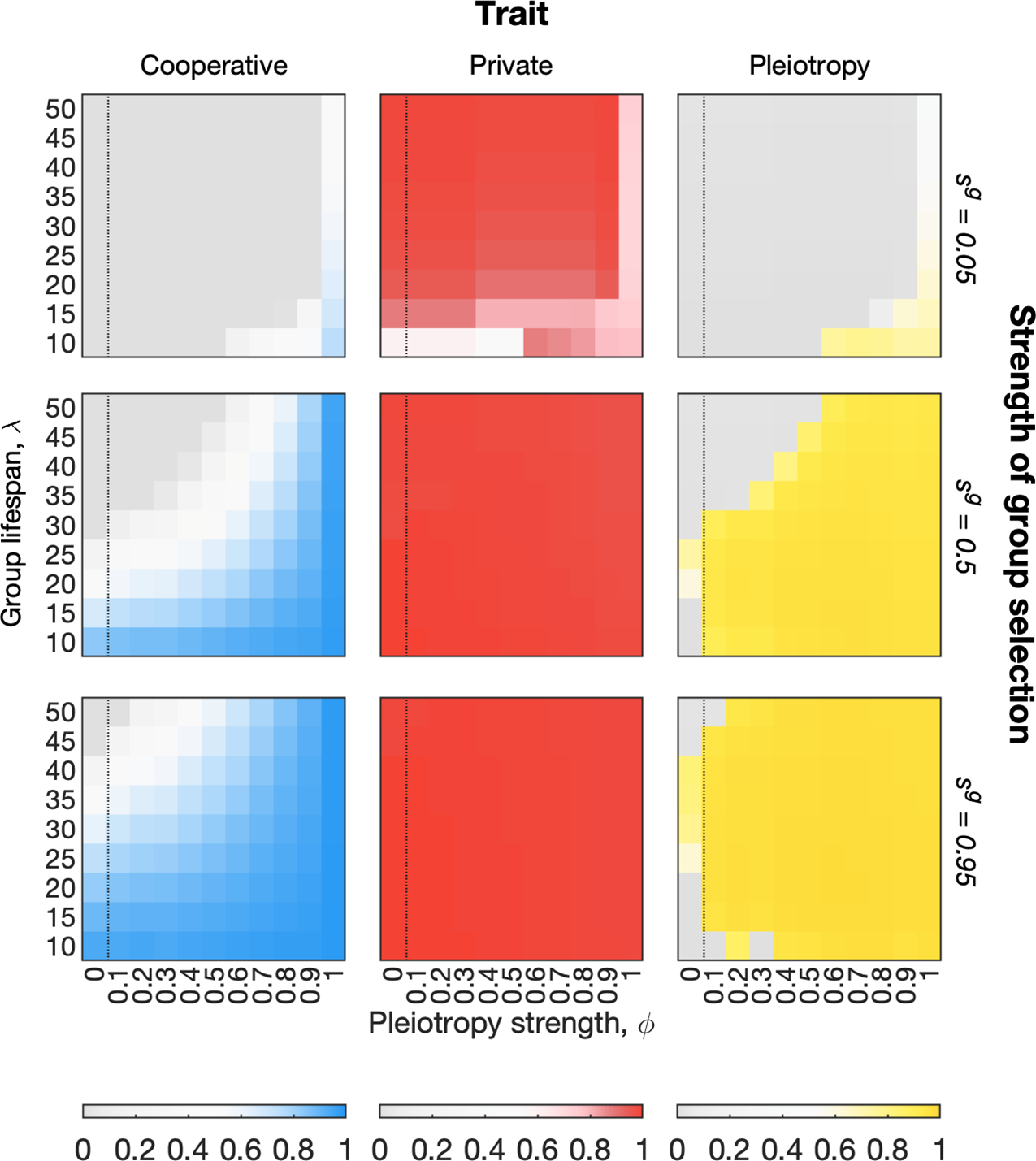


**S13 Fig. Pleiotropy evolves to stabilise cooperation even when group selection is weak.** We explored a model in which the strength of group selection, $s^{g}$, is varied. Heatmaps show average trait values among the global population of cells (across all groups) at steady state in our model. Results are shown for three strengths of group selection (increasing from top to bottom). Stronger group selection favours both cooperation and pleiotropy, but strong pleiotropy can help cooperation evolve even when group selection is weak, ($s^{g}=0.05)$. The dotted line marks the boundary between pleiotropy having no effect (control case) and pleiotropy having an effect on the outcome of mutations. Parameters: $s^{c}=0.95$; $K=200$; $\mu=0.0001$; $\nu=0.01; K=200$. The code required to generate this Figure can be found at https://github.com/euler-mab/pleiotropy and https://zenodo.org/record/6367788#.YjSBVurP2Uk.
