# Supplementary material for: Pleiotropic constraints promote the evolution of cooperation in cellular groups
Source: PLoS Biol. 2022 Jun 3;20(6):e3001626. doi: 10.1371/journal.pbio.3001626 (PMC9166655; doi:10.1371/journal.pbio.3001626)
Supplement: S14 Fig — We varied the relative rate of gain-of-function mutations to loss-of-function mutations, ν. Heatmaps show average trait values among the global population of cells (across all groups) at steady state in our model. Results are shown for 3 gain-of-function rates (increasing from top to bottom). The gain-of-function ratio had a marginal effect on the evolution of pleiotropy, favouring slightly higher rates when ν is higher. The dotted line marks the boundary between pleiotropy having no effect (control case) and pleiotropy having an effect on the outcome of mutations. Parameters: sc = sg = 0.95; K = 200; μ = 0.0001; K = 200. The code required to generate this figure can be found at https://github.com/euler-mab/pleiotropy and https://zenodo.org/record/6367788#.YjSBVurP2Uk. (DOCX) [file pbio.3001626.s015.docx]

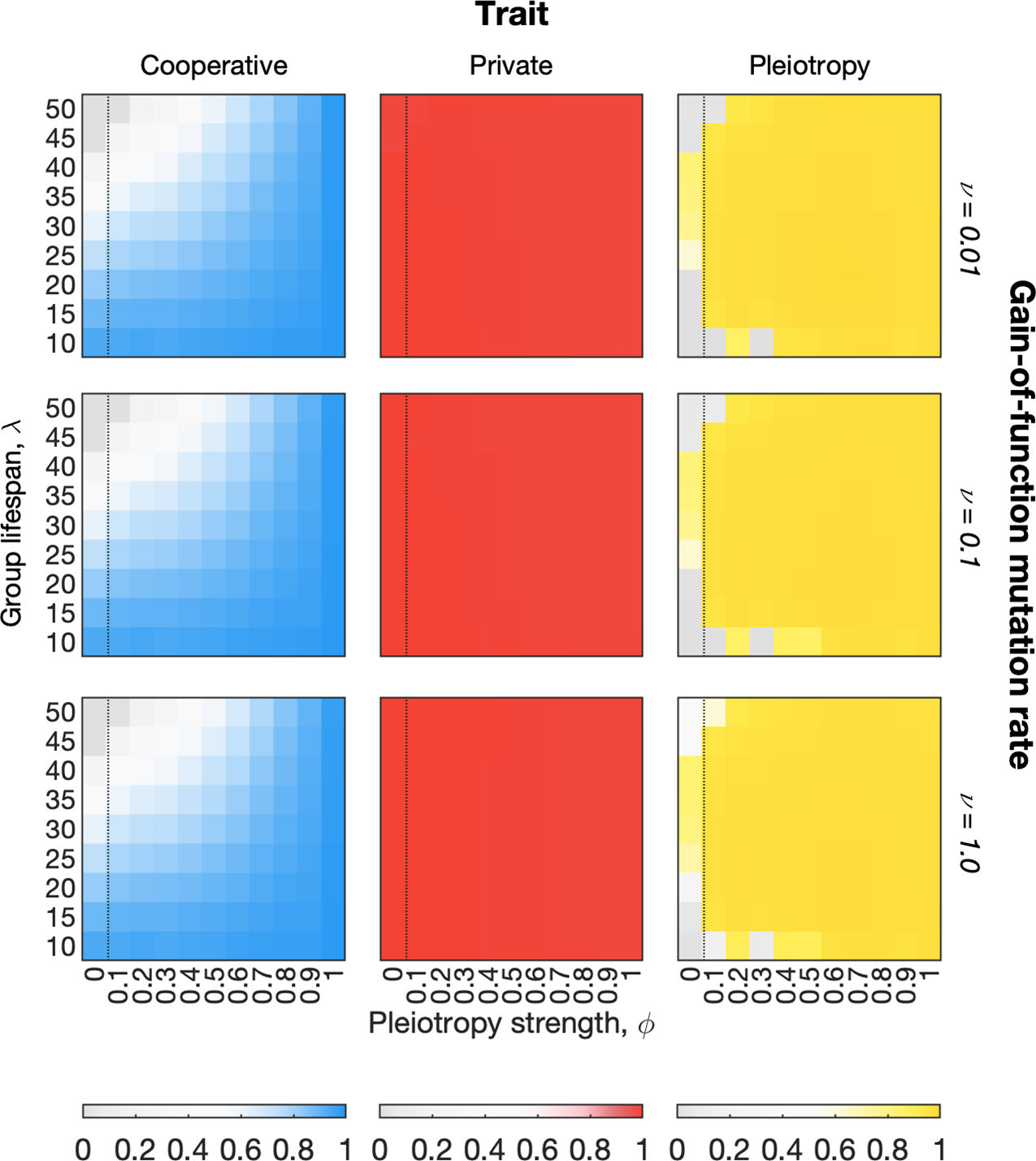


**S14 Fig. The rate of gain-of-function mutations has little impact on the dynamics of pleiotropy evolution.** We varied the relative rate of gain-of-function mutations to loss-of-function mutations, $\nu$. Heatmaps show average trait values among the global population of cells (across all groups) at steady state in our model. Results are shown for three gain-of-function rates (increasing from top to bottom). The gain-of-function ratio had a marginal effect on the evolution of pleiotropy, favouring slightly higher rates when $\nu$ is higher. The dotted line marks the boundary between pleiotropy having no effect (control case) and pleiotropy having an effect on the outcome of mutations. Parameters: $s^{c}=s^{g}=0.95$; $K=200$; $\mu=0.0001$; $K=200$. The code required to generate this Figure can be found at https://github.com/euler-mab/pleiotropy and https://zenodo.org/record/6367788#.YjSBVurP2Uk.
