# Supplementary material for: Pleiotropic constraints promote the evolution of cooperation in cellular groups
Source: PLoS Biol. 2022 Jun 3;20(6):e3001626. doi: 10.1371/journal.pbio.3001626 (PMC9166655; doi:10.1371/journal.pbio.3001626)
Supplement: S15 Fig — We varied the loss-of-function mutation rate, μ. Heatmaps show average trait values among the global population of cells (across all groups) at steady state in our model. Results are shown for 3 loss-of-function rates (increasing from top to bottom). Higher mutation rates disfavoured the evolution of cooperation, but pleiotropy still evolved at higher strengths of pleiotropy and for lower group sizes. The evolution of pleiotropy was associated with a stabilisation of cooperation even at higher mutation rates. The dotted line marks the boundary between pleiotropy having no effect (control case) and pleiotropy having an effect on the outcome of mutations. Parameters: sc = sg = 0.95; K = 200; ν = 0.01. The code required to generate this figure can be found at https://github.com/euler-mab/pleiotropy and https://zenodo.org/record/6367788#.YjSBVurP2Uk. (DOCX) [file pbio.3001626.s016.docx]

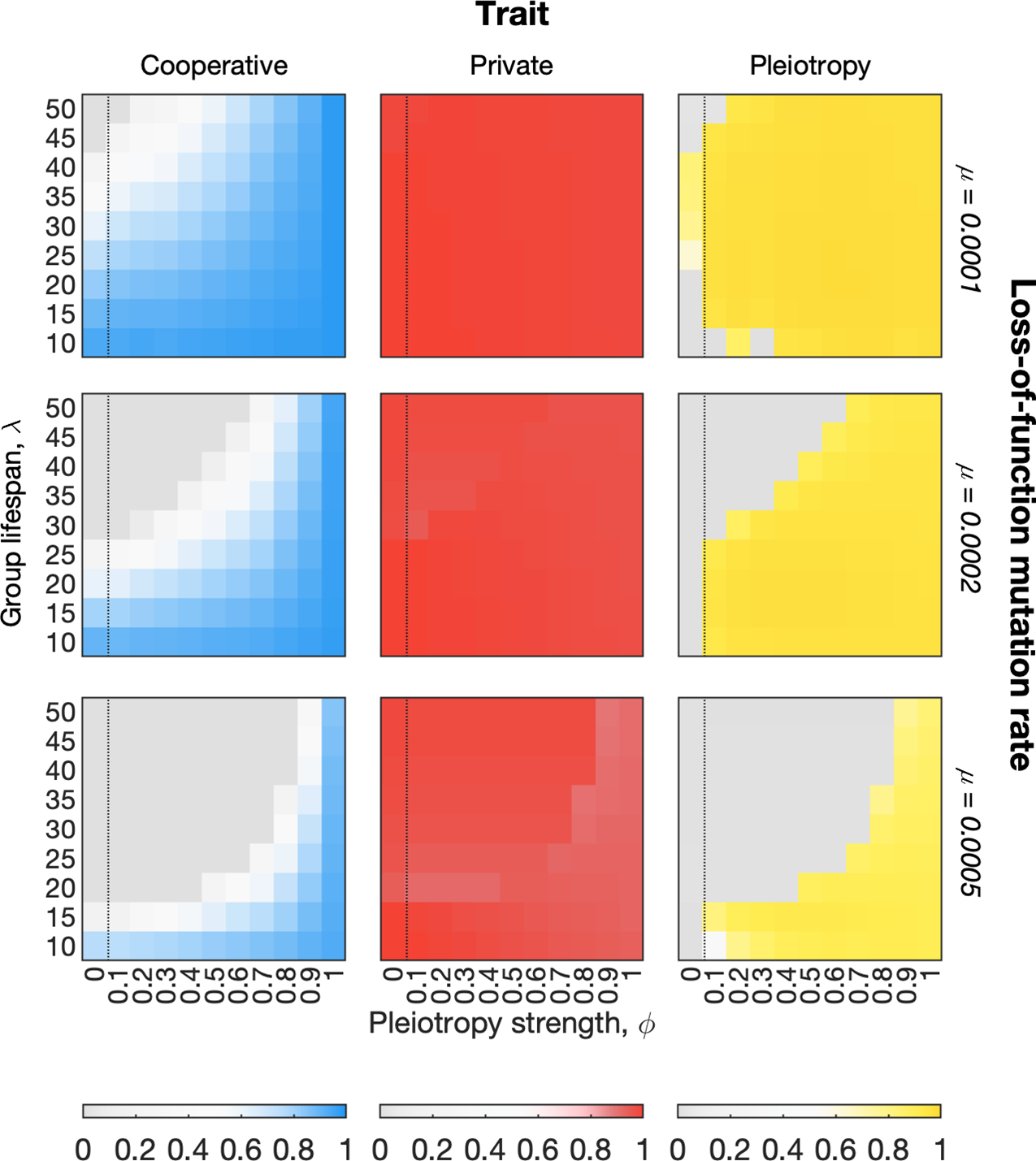


**S15 Fig. Strong pleiotropy can help rescue cooperation even in the face of high mutation rates.** We varied the loss-of-function mutation rate, $\mu$. Heatmaps show average trait values among the global population of cells (across all groups) at steady state in our model. Results are shown for three loss-of-function rates (increasing from top to bottom). Higher mutation rates disfavoured the evolution of cooperation, but pleiotropy still evolved at higher strengths of pleiotropy and for lower group sizes. The evolution of pleiotropy was associated with a stabilisation of cooperation even at higher mutation rates. The dotted line marks the boundary between pleiotropy having no effect (control case) and pleiotropy having an effect on the outcome of mutations. Parameters: $s^{c}=s^{g}=0.95$; $K=200$; $\nu=0.01$. The code required to generate this Figure can be found at https://github.com/euler-mab/pleiotropy and https://zenodo.org/record/6367788#.YjSBVurP2Uk.
