# Supplementary material for: Pleiotropic constraints promote the evolution of cooperation in cellular groups
Source: PLoS Biol. 2022 Jun 3;20(6):e3001626. doi: 10.1371/journal.pbio.3001626 (PMC9166655; doi:10.1371/journal.pbio.3001626)
Supplement: S16 Fig — We varied the loss-of-function mutation rate, μ, using values 10-fold lower than the typical value used in the main text. Heatmaps show average trait values among the global population of cells (across all groups) at steady state in our model. Results are shown for 3 loss-of-function rates (increasing from top to bottom). With 10-fold lower mutation rates, we found that cooperation breaks down only for longer group life spans, λ. When cooperation is threatened by breakdown, however, we still found that pleiotropy evolves to rescue it. The dotted line marks the boundary between pleiotropy having no effect (control case) and pleiotropy having an effect on the outcome of mutations. Parameters: sc = sg = 0.95; K = 200; ν = 0.01; c = 0. The code required to generate this figure can be found at https://github.com/euler-mab/pleiotropy and https://zenodo.org/record/6367788#.YjSBVurP2Uk. (DOCX) [file pbio.3001626.s017.docx]

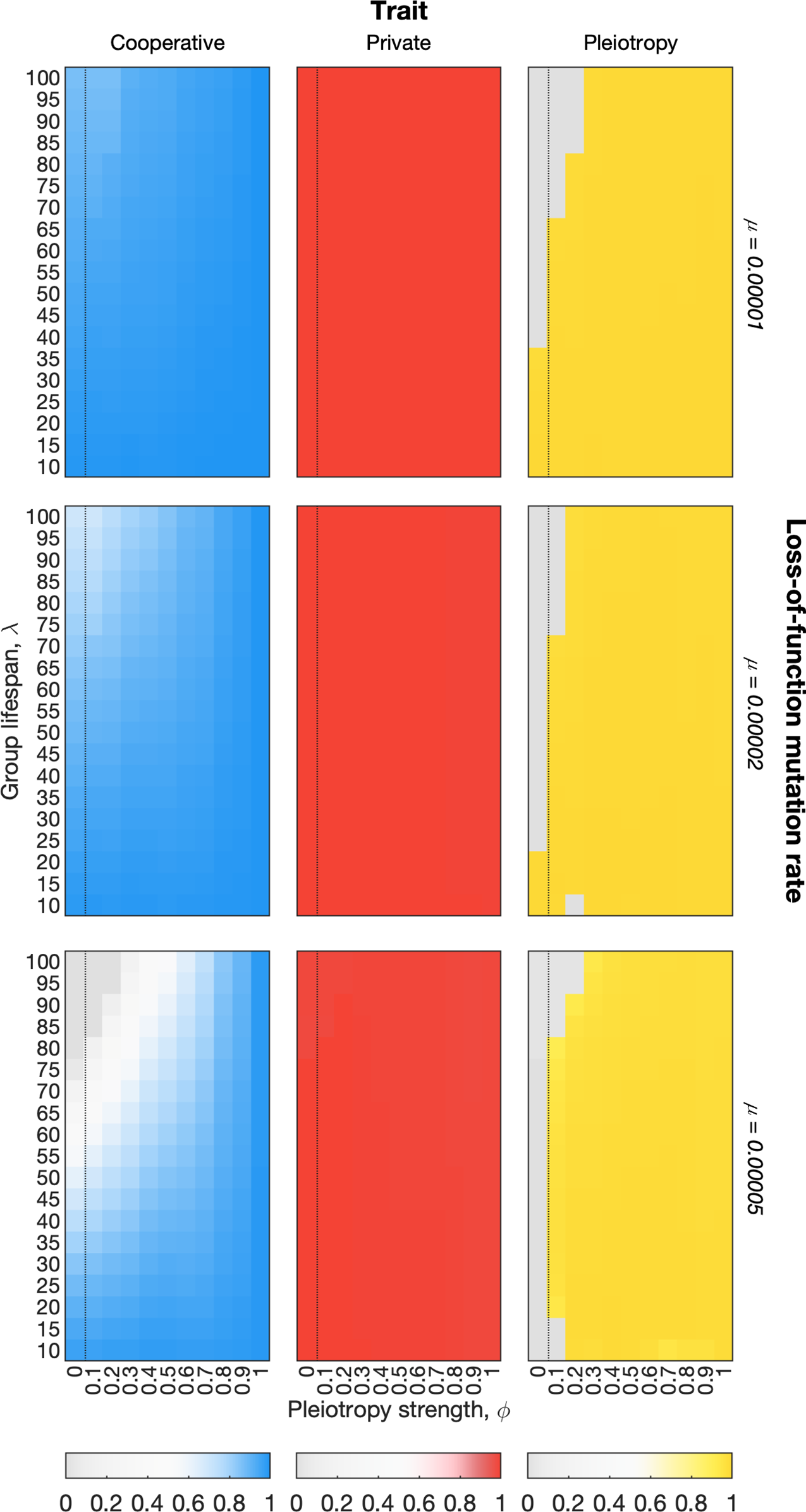


**S16 Fig. Lower mutation rates increases cooperation but pleiotropy still evolves.** We varied the loss-of-function mutation rate, $\mu$, using values ten-fold lower than the typical value used in the main text. Heatmaps show average trait values among the global population of cells (across all groups) at steady state in our model. Results are shown for three loss-of-function rates (increasing from top to bottom). With ten-fold lower mutation rates, we found that cooperation breaks down only for longer group lifespans, $\lambda$. When cooperation is threatened by breakdown, however, we still found that pleiotropy evolves to rescue it. The dotted line marks the boundary between pleiotropy having no effect (control case) and pleiotropy having an effect on the outcome of mutations. Parameters: $s^{c}=s^{g}=0.95$; $K=200$; $\nu=0.01;c=0$. The code required to generate this Figure can be found at https://github.com/euler-mab/pleiotropy and https://zenodo.org/record/6367788#.YjSBVurP2Uk.
