# Supplementary material for: Pleiotropic constraints promote the evolution of cooperation in cellular groups
Source: PLoS Biol. 2022 Jun 3;20(6):e3001626. doi: 10.1371/journal.pbio.3001626 (PMC9166655; doi:10.1371/journal.pbio.3001626)
Supplement: S17 Fig — We varied the gain-of-function mutation rate, μ, using values 10-fold lower than the typical value used in the main text, and assumed that the evolution of pleiotropy decreases group function by 2%. Heatmaps show average trait values among the global population of cells (across all groups) at steady state in our model. Results are shown for 3 loss-of-function rates (increasing from top to bottom). With 10-fold lower mutation rates and a cost, we found that pleiotropy evolves most frequently when the mutation rate is higher and cooperation is under the greatest threat. The dotted line marks the boundary between pleiotropy having no effect (control case) and pleiotropy having an effect on the outcome of mutations. Parameters: sc = sg = 0.95; K = 200; ν = 0.01; ζ = 0.02. The code used to produce this figure may be found at https://github.com/euler-mab/pleiotropy. (DOCX) [file pbio.3001626.s018.docx]

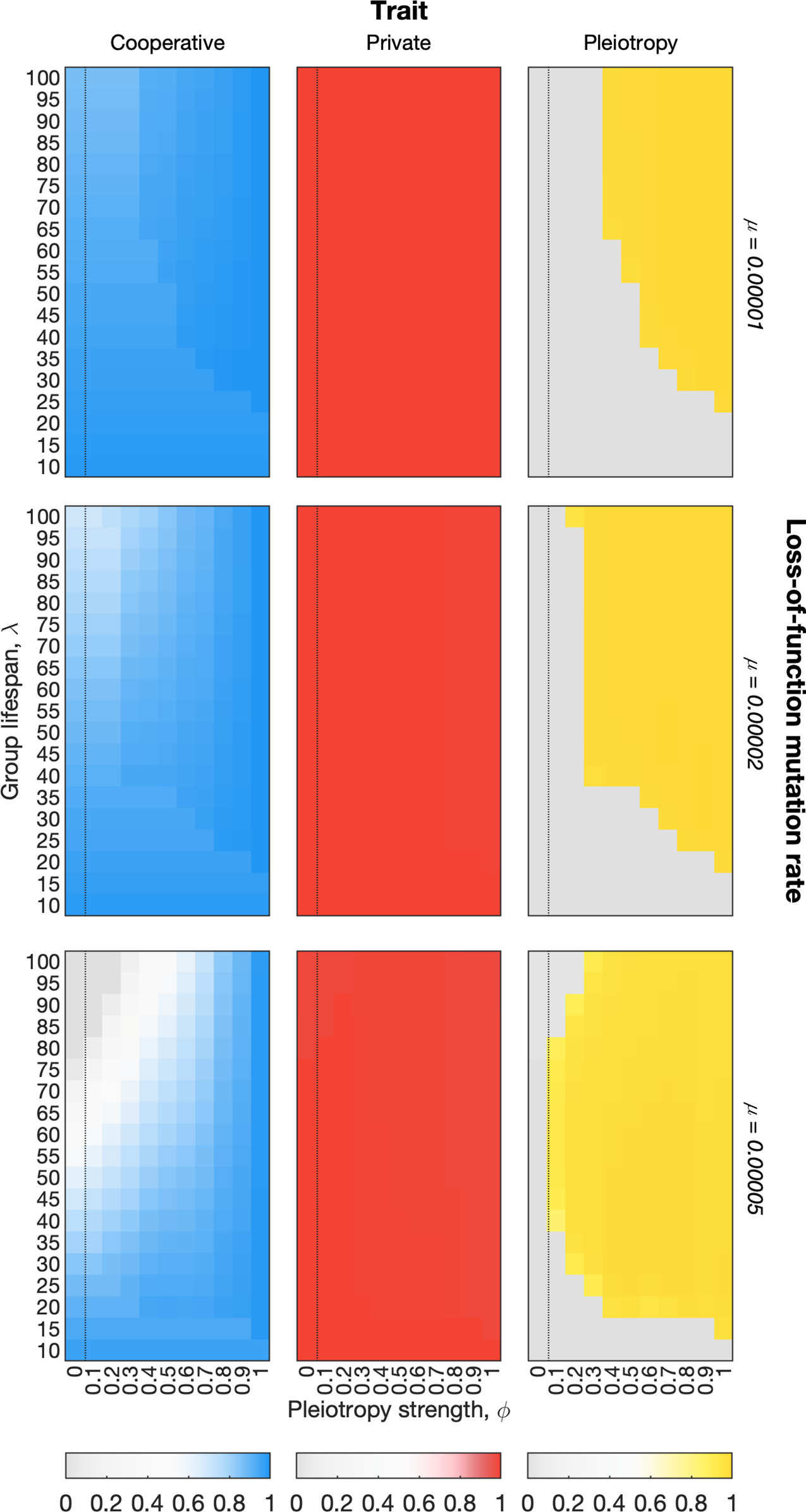


**S17 Fig. Lower mutation rates increase cooperation but pleiotropy still evolves, even when pleiotropy is costly.** We varied the gain-of-function mutation rate, $\mu$, using values ten-fold lower than the typical value used in the main text, and assumed that the evolution of pleiotropy decreases group function by 2%. Heatmaps show average trait values among the global population of cells (across all groups) at steady state in our model. Results are shown for three loss-of-function rates (increasing from top to bottom). With ten-fold lower mutation rates and a cost, we found that pleiotropy evolves most frequently when the mutation rate is higher and cooperation is under the greatest threat. The dotted line marks the boundary between pleiotropy having no effect (control case) and pleiotropy having an effect on the outcome of mutations. Parameters: $s^{c}=s^{g}=0.95$; $K=200$; $\nu=0.01;\zeta=0.02$. The code used to produce this figure may be found at https://github.com/euler-mab/pleiotropy.
