# Supplementary material for: Pleiotropic constraints promote the evolution of cooperation in cellular groups
Source: PLoS Biol. 2022 Jun 3;20(6):e3001626. doi: 10.1371/journal.pbio.3001626 (PMC9166655; doi:10.1371/journal.pbio.3001626)
Supplement: S18 Fig — We varied the age fraction of the expected life span at which groups reach reproductive maturity, α, and assumed that the evolution of pleiotropy decreases group function by 2%. Heatmaps show average trait values among the global population of cells (across all groups) at steady state in our model. Results are shown for 3 reproductive maturity parameters (increasing the age at which maturity is reached from top to bottom). Increasing the age of reproductive maturity has a marginal positive effect on the evolution of pleiotropy for shorter life spans, but a marginal negative effect on cooperation. The dotted line marks the boundary between pleiotropy having no effect (control case) and pleiotropy having an effect on the outcome of mutations. Parameters: sc = sg = 0.95; K = 200; ν = 0.01; ζ = 0.02. The code required to generate this figure can be found at https://github.com/euler-mab/pleiotropy and https://zenodo.org/record/6367788#.YjSBVurP2Uk. (DOCX) [file pbio.3001626.s019.docx]

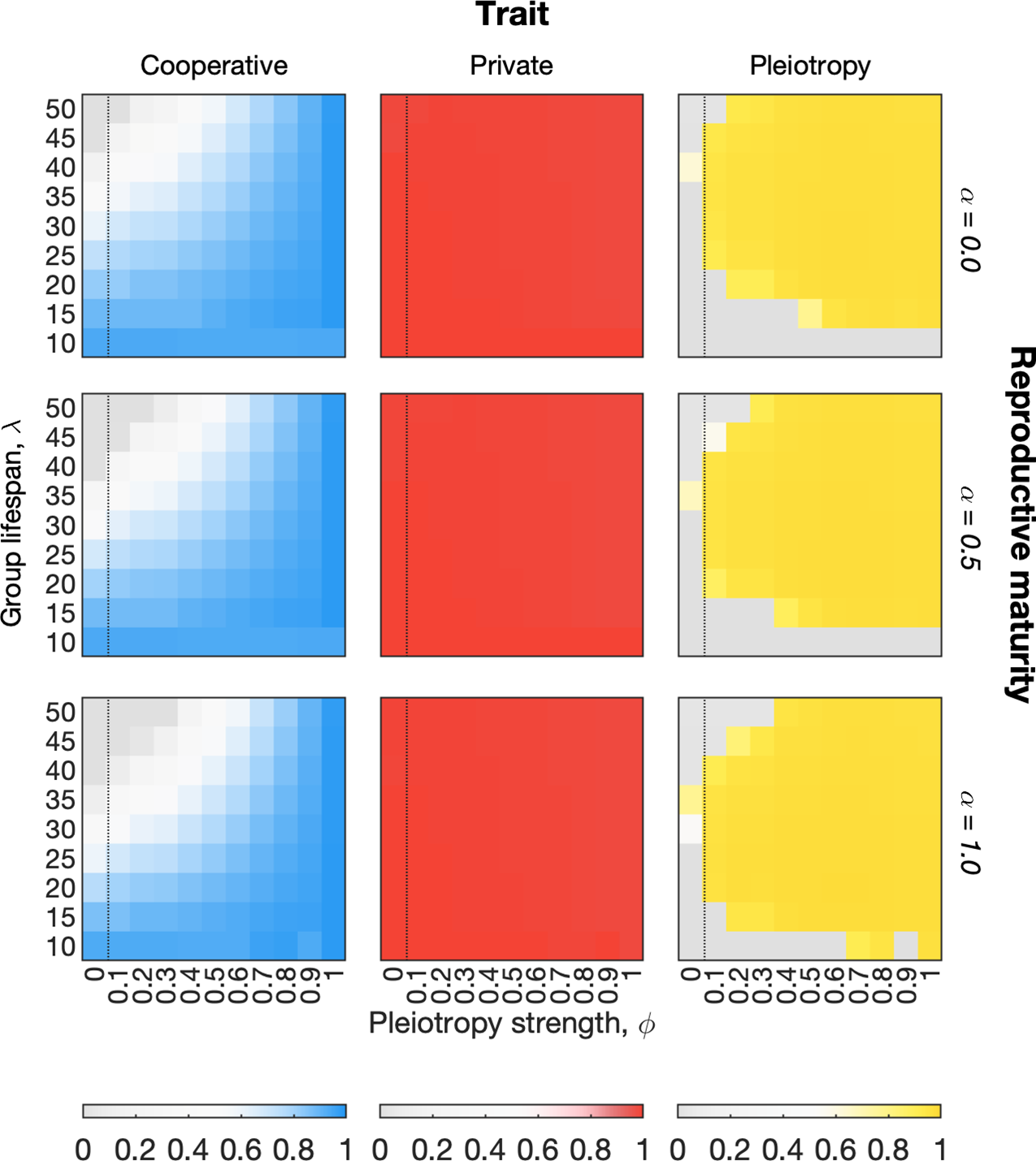


**S18 Fig. Reproduction at maturity hinders cooperation and has mixed effects on the evolution of pleiotropy.** We varied the age fraction of the expected lifespan at which groups reach reproductive maturity, $\alpha$, and assumed that the evolution of pleiotropy decreases group function by 2%. Heatmaps show average trait values among the global population of cells (across all groups) at steady state in our model. Results are shown for three reproductive maturity parameters (increasing the age at which maturity is reached from top to bottom). Increasing the age of reproductive maturity has a marginal positive effect on the evolution of pleiotropy for shorter lifespans, but a marginal negative effect on cooperation. The dotted line marks the boundary between pleiotropy having no effect (control case) and pleiotropy having an effect on the outcome of mutations. Parameters: $s^{c}=s^{g}=0.95$; $K=200$; $\nu=0.01;\zeta=0.02$. The code required to generate this Figure can be found at https://github.com/euler-mab/pleiotropy and https://zenodo.org/record/6367788#.YjSBVurP2Uk.
