# Supplementary material for: Pleiotropic constraints promote the evolution of cooperation in cellular groups
Source: PLoS Biol. 2022 Jun 3;20(6):e3001626. doi: 10.1371/journal.pbio.3001626 (PMC9166655; doi:10.1371/journal.pbio.3001626)
Supplement: S19 Fig — We modified the individual-based model of dos Santos and colleagues to compare what happens when pleiotropy cannot evolve (left) as compared to when it can evolve (right). We varied the length of the within-group growth phase k during which spontaneous mutants can arise and invade within groups, and the relatedness r at the point at which groups form. We follow the evolution of a private trait, cooperative trait, and pleiotropy trait. (A) Evolutionary dynamics of all 3 traits for a within-group growth phase of k = 30 and r = 1. (B) Steady-state levels of all 3 traits under a when the length of the within-group growth phase is varied (x-axis) for r = 1. (C) Steady-state levels of all 3 traits when relatedness at the point at which groups form is varied for a within-group growth phase of k = 30. Other parameters: b = 0.11, c = 0.1, g = 0.5, mutation rate μ = 0.001, number of groups ng = 1000. All plots are averages of 10 replicates. The code required to generate this figure can be found at https://github.com/euler-mab/pleiotropy and https://zenodo.org/record/6367788#.YjSBVurP2Uk. (DOCX) [file pbio.3001626.s020.docx]

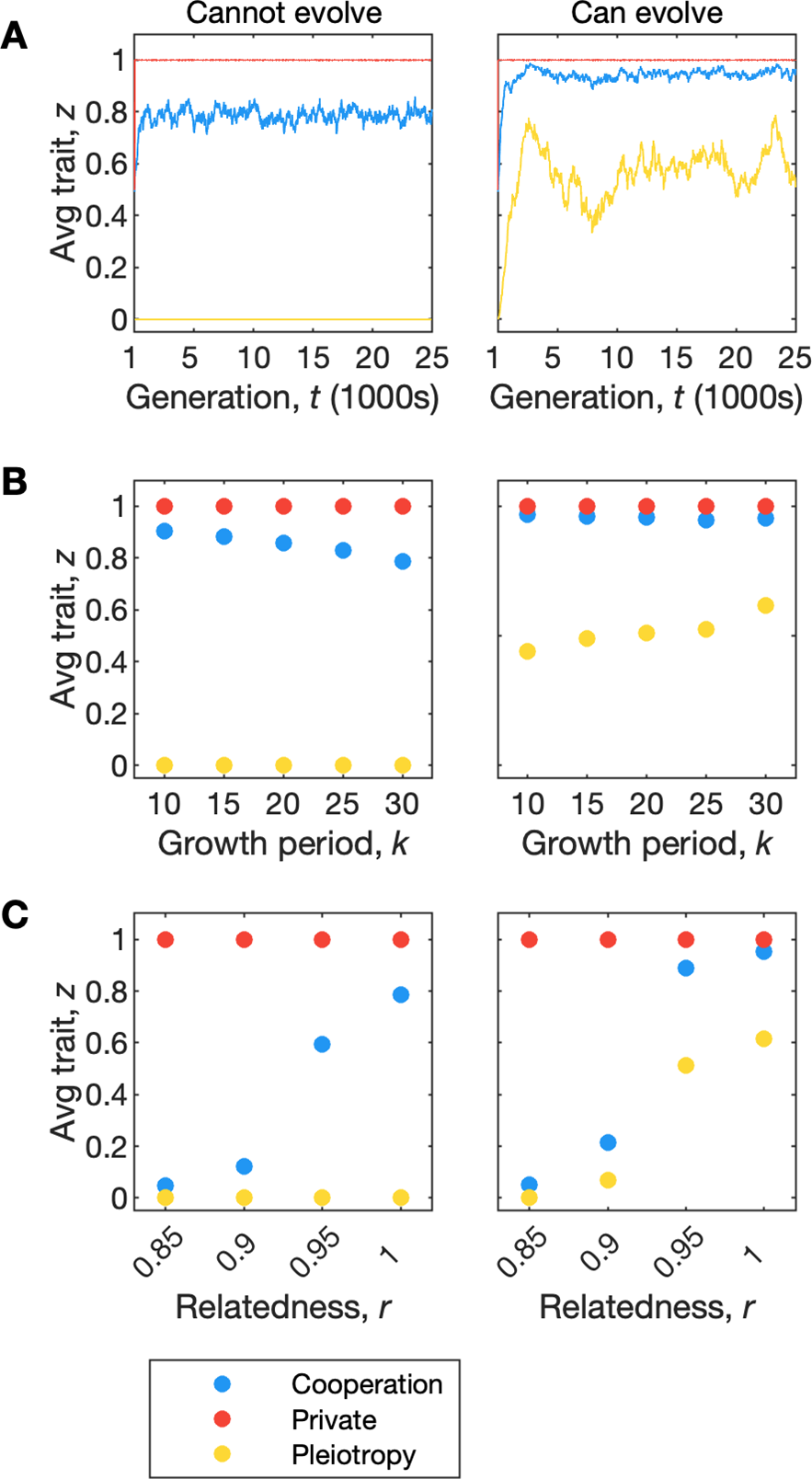


**S19 Fig. When mutation-driven breakdown of cooperation is a problem, pleiotropy stabilises cooperation.** We modified the individual-based model of dos Santos et al to compare what happens when pleiotropy cannot evolve (left) as compared to when it can evolve (right). We varied the length of the within-group growth phase $k$ during which spontaneous mutants can arise and invade within groups, and the relatedness $r$ at the point at which groups form. We follow the evolution of a private trait, cooperative trait, and pleiotropy trait. (A) Evolutionary dynamics of all three traits for a within-group growth phase of $k = 30$ and $r = 1$. (B) Steady-state levels of all three traits under a when the length of the within-group growth phase is varied (x-axis) for $r = 1$. (C) Steady-state levels of all three traits when relatedness at the point at which groups form is varied for a within-group growth phase of $k = 30$. Other parameters: $b=0.11, c=0.1, g=0.5$, mutation rate $\mu= 0.001$, number of groups $n_{g}=1000$. All plots are averages of 10 replicates. The code required to generate this Figure can be found at https://github.com/euler-mab/pleiotropy and https://zenodo.org/record/6367788#.YjSBVurP2Uk.
