# Supplementary material for: Pleiotropic constraints promote the evolution of cooperation in cellular groups
Source: PLoS Biol. 2022 Jun 3;20(6):e3001626. doi: 10.1371/journal.pbio.3001626 (PMC9166655; doi:10.1371/journal.pbio.3001626)
Supplement: S20 Fig — We performed simulations using the mutation model used by dos Santos and colleagues in the production of their S5 Fig. This again reveals conditions where pleiotropy evolves and increases cooperation as it does. We varied the length of the within-group growth phase k during which spontaneous mutants can arise and invade within groups, and the relatedness r at the point at which groups form. We follow the evolution of a private trait, cooperative trait, and pleiotropy trait. (A) Evolutionary dynamics of all 3 traits for a within-group growth phase of k = 30 and r = 1. (B) Steady-state levels of all 3 traits under a when the length of the within-group growth phase is varied (x-axis) for r = 1. (C) Steady-state levels of all 3 traits when relatedness at the point at which groups form is varied for a within-group growth phase of k = 30. Other parameters: b = 0.11, c = 0.1, g = 0.5, mutation rate μ = 0.001, number of groups ng = 1000. All plots are averages of 10 replicates. The code required to generate this figure can be found at https://github.com/euler-mab/pleiotropy and https://zenodo.org/record/6367788#.YjSBVurP2Uk. (DOCX) [file pbio.3001626.s021.docx]

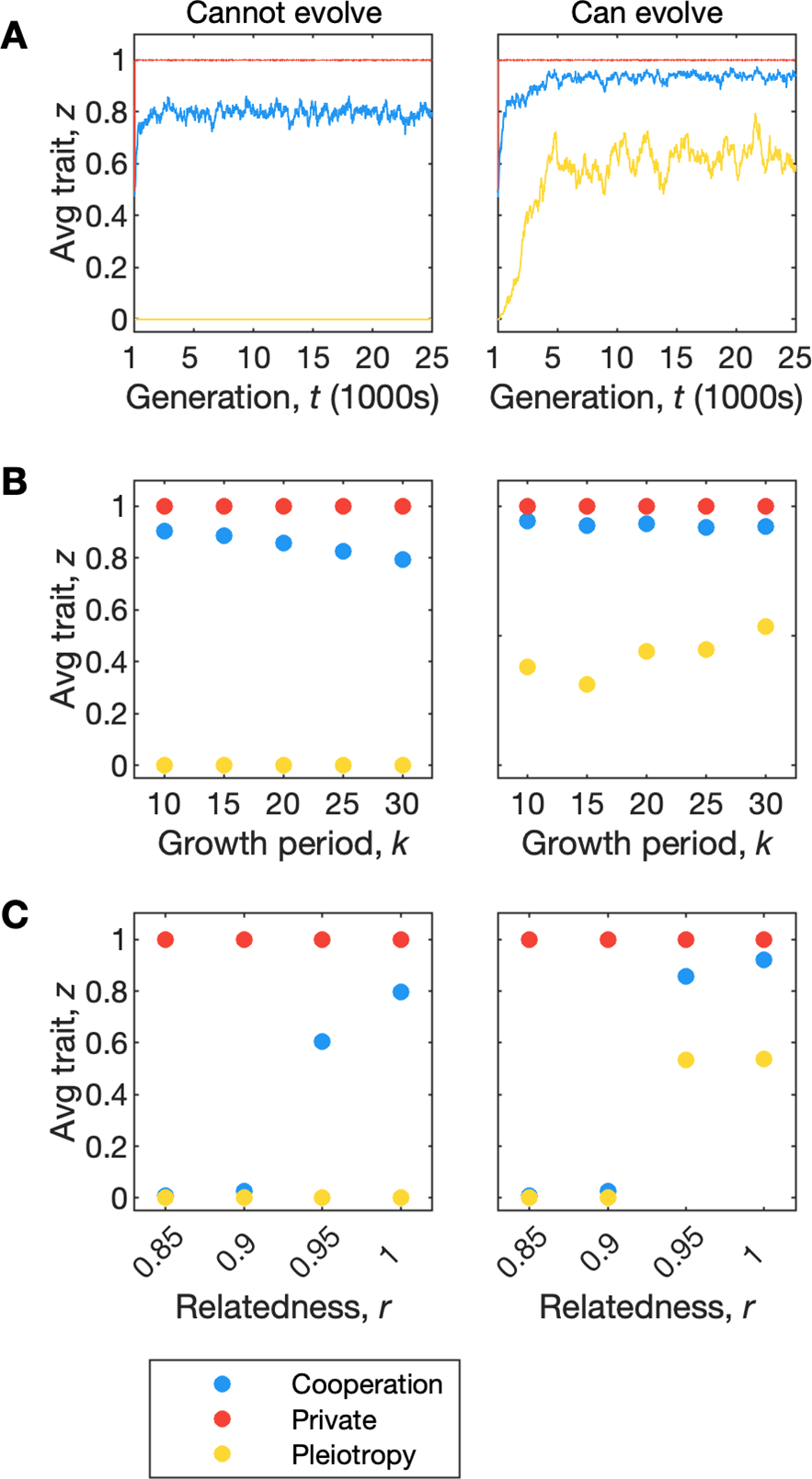


**S20 Fig. Pleiotropy evolves to stabilise cooperation even when the loss-of-function mutation rate is 10x greater than the gain-of-function rate.** We performed simulations using the mutation model used by dos Santos et al in the production of their S5 Fig. This again reveals conditions where pleiotropy evolves and increases cooperation as it does. We varied the length of the within-group growth phase $k$ during which spontaneous mutants can arise and invade within groups, and the relatedness $r$ at the point at which groups form. We follow the evolution of a private trait, cooperative trait, and pleiotropy trait. (A) Evolutionary dynamics of all three traits for a within-group growth phase of $k = 30$ and $r = 1$. (B) Steady-state levels of all three traits under a when the length of the within-group growth phase is varied (x-axis) for $r = 1$. (C) Steady-state levels of all three traits when relatedness at the point at which groups form is varied for a within-group growth phase of $k = 30$. Other parameters: $b=0.11, c=0.1, g=0.5$, mutation rate $\mu= 0.001$, number of groups $n_{g}=1000$. All plots are averages of 10 replicates. The code required to generate this Figure can be found at https://github.com/euler-mab/pleiotropy and https://zenodo.org/record/6367788#.YjSBVurP2Uk.
